# Conjugation as a Tool in Therapeutics: Role of Amino Acids/Peptides-Bioactive (Including Heterocycles) Hybrid Molecules in Treating Infectious Diseases

**DOI:** 10.3390/antibiotics12030532

**Published:** 2023-03-07

**Authors:** Rohith Gattu, Sanjay S. Ramesh, Siddaram Nadigar, Channe Gowda D, Suhas Ramesh

**Affiliations:** 1 Postgraduate Department of Chemistry, JSS College of Arts, Commerce and Science, Ooty Road, Mysuru 570025, Karnataka, India; 2 Department of Studies in Chemistry, Manasagangotri, University of Mysore, Mysuru 570005, Karnataka, India

**Keywords:** conjugation chemistry, amino acids, peptides, bioactive molecules, heterocycles

## Abstract

Peptide-based drugs are gaining significant momentum in the modern drug discovery, which is witnessed by the approval of new drugs by the FDA in recent years. On the other hand, small molecules-based drugs are an integral part of drug development since the past several decades. Peptide-containing drugs are placed between small molecules and the biologics. Both the peptides as well as the small molecules (mainly heterocycles) pose several drawbacks as therapeutics despite their success in curing many diseases. This gap may be bridged by utilising the so called ‘conjugation chemistry’, in which both the partners are linked to one another through a stable chemical bond, and the resulting conjugates are found to possess attracting benefits, thus eliminating the stigma associated with the individual partners. Over the past decades, the field of molecular hybridisation has emerged to afford us new and efficient molecular architectures that have shown high promise in medicinal chemistry. Taking advantage of this and also considering our experience in this field, we present herein a review concerning the molecules obtained by the conjugation of peptides (amino acids) to small molecules (heterocycles as well as bioactive compounds). More than 125 examples of the conjugates citing nearly 100 references published during the period 2000 to 2022 having therapeutic applications in curing infectious diseases have been covered.

## 1. Introduction

### 1.1. Medicinal Chemistry and Its Significance

Science, and the way the researchers think, are rapidly changing. Modern medicinal chemistry may be said to have its roots in the early 19th century with the development of the side-chain concept of drug action by Berlin immunologist, Ehrlich, in 1885, followed by the conception of the term ‘chemotherapy’, defining it as ‘the chemical entities exhibiting selective toxicities against particular infectious agent’ [1,2]. The term ‘medicinal chemistry’ refers to a mature, interdependent science that unites both applied (medicine) and fundamental (chemistry) sciences. It includes the identification, discovery, development and interpretation of the molecular mechanisms that govern the biological activity of the substances [3]. Medicinal chemistry still continues to play a vital role in drug research and development with the help of developed novel techniques and enhanced knowledge from various branches of related science [4]. In order to maximise the effectiveness and minimise the side effects, medicinal chemists play a key role in driving the drug discovery project by drawing on their knowledge of modern organic chemistry, disease biology, in vitro and in vivo pharmacological screening and pharmacokinetic characteristics [5].

**Figure 1:**
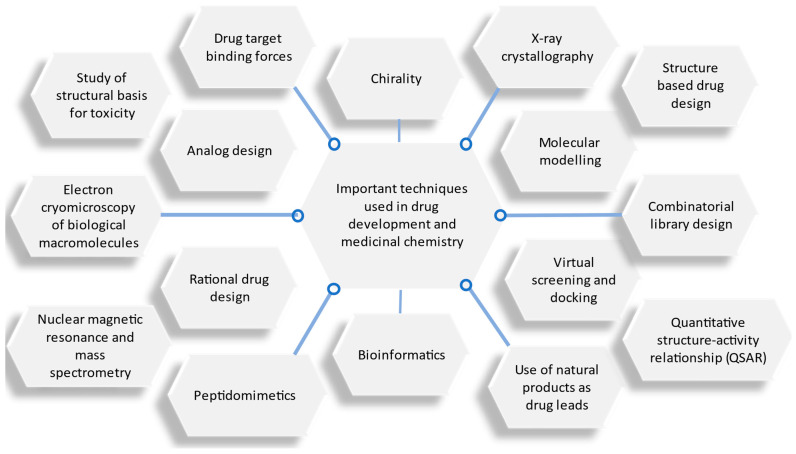
Various techniques used in drug discovery.

Over the past 25 years, the role of the medicinal chemist has undergone profound transformation. In the early years of drug development viz. the 1950s, medicinal chemists mainly relied on the information from in vivo testing, whereas from the late 1980s till date, the development of novel technologies such as high-throughput in vitro screening, combinatorial technology, large compound libraries, structure-based drug design and defined molecular targets have altered the previous straightforward landscape (Figure 1) [6].

The primary goals of medicinal chemistry include: (i) to identify and discover lead compounds and chemical probes for the least understood biological targets, (ii) to demonstrate the target druggability and (iii) to address factors that affect the effectiveness or failure of drugs [7,8]. Most significantly, medicinal chemistry allows the recognition of clinical candidates and provides new strategies focused at strengthening the scope and quality of hit and lead finding stages that, although often ignored, are essential to minimise attrition in drug discovery. The lack of effectiveness and safety is indeed the major cause for drug failures, which can be linked with the target and/or the chemical framework of the lead compound series [9,10,11]. Therefore, the selection of right lead to explore is the essence which can have an impact on the rate of success in modern drug discovery. Over the past 30 years, various approaches have been proposed to determine and to explore the lead series, such as parallel chemical investigations based on diversity-oriented synthesis (DOS) and combinatorial chemistry. Despite the advancements being made, finding new leads continues to be a challenging task that demands a significant amount of cost and time. According to estimates, an average of 20 hit-to-lead investigations, 15 lead optimisation programmes and a cost of USD 600 million are required for the approval of a single drug [12]. The information regarding the biological targets, its consequences on disease mechanisms and potential medicinal applications were limited until 1980 [6]. Due to the lack of in vitro screening capabilities, compounds were designed and synthesised individually in gram quantities to satisfy the requirements for testing materials in animal models. Given the limited tools and techniques available at that time, these syntheses were often dangerous, time-consuming and poorly efficient. The output revealed that very few products were developed on a weekly basis with limited compound libraries for lead optimisation. In order to overcome these drawbacks, many achievements, innovations and serendipitous findings were contributed by researchers with creativity and intuition. The modern day drug discovery involves the technology-driven process which was originated from a chemistry-inspired/pharmacology-driven method. The advent of computational modelling, machine learning (ML), artificial intelligence (AI) and high-throughput screening (HTS) has enabled the swift design and evaluation of an enormous number of compounds [13,14]. Nowadays, ML and AI are equally contributing in medicinal chemistry.

### 1.2. Infections and Their Deleterious Effects

The human population presently suffers and presumptively has suffered for thousands of years by the infectious diseases identical or similar to the diseases of other wild primate populations. However, the most significant infectious diseases of current food-producing human populations also comprises various diseases which could have been originated only within the last 11,000 years, following the advancements in agriculture [15,16]. The global rise in the multidrug-resistant diseases, along with a constant decline in the discovery of novel antibiotics, emphasises the need for novel therapeutics to control the infections [17]. In the beginning of the 20th century, infectious diseases were the primary cause of death worldwide [18]. There are over 1400 varied infectious agents which have been reported to induce disease in human beings [19,20,21]. The average lifespan used to be 47, in major part due to increased infant and childhood mortality by infection. An infant born in the early 1900s had nearly a 10% risk of dying among the ages of one and four, mostly due to diarrhoeal and pneumonia disease [22].

Surprisingly, by 1900, the majority of the developed world had witnessed a significant improvement in mortality and morbidity from infectious diseases. Between 1700 and 1900, the average lifespan in developed countries improved from 17 to 52 years of age [23], and the fatality from tuberculosis dropped by 80% [24]. The developing world had not possessed similar accomplishments with infectious diseases: there, the infections continued to be the primary factor for mortality and morbidity. In 1998, the World Health Organisation evaluated that infectious diseases induced over 13 million deaths, which was about 25% of 54 million deaths worldwide [25]. Among the most common reasons for mortality were the three diseases that have been so normal in the advanced world since the beginning of the 20th century: tuberculosis (1.5 million), diarrhoeal disease (2.2 million) and pneumonia (3.5 million). Other significant infections causes for mortality were measles (1.0 million), malaria (~1.1 million) and AIDS (2.3 million). The enhanced life expectancy and declined mortality by infectious diseases were ascribed to a number of factors, such as reducing host susceptibility and preventing disease transmission, improved housing and nutrition, better food and water, and enhanced sanitation and hygiene [26].

Although the number of infectious disease fatalities was already dropping, the development of antimicrobial drugs in the mid-twentieth century hastened this trend even further [27]. This decline coincided with the birth of an era of antibiotics. During 1900 and 1980, the number of deaths caused by infectious diseases dropped from 797 to 36 per 100,000 [28]. By the end of the 20th century, deaths caused by infectious diseases had been substituted by chronic diseases, such as heart diseases, stroke and cancer. The technological and societal developments that had an impact on infectious diseases in the advanced world had the least degree of influence in the developing world [26].

By the end of the 20th century, there were concerning trends in both the advanced and developing worlds. Novel infectious diseases and microorganisms were being reformed. The infectious diseases are also being reformed as the cause of chronic illnesses. Novel infectious agents, such as Marburg/Ebola virus/corona virus, possess the capabilities for swift international spread. Infections which were once controlled in many regions of the world such as malaria, yellow fever, dengue fever, tuberculosis and cholera are re-emerging. Resistivity towards antimicrobial therapeutics is becoming a severe global issue. Even in the modern world, the infectious disease death rate is growing [28].

### 1.3. Amino Acids/Peptides-Based Drugs—A Renaissance in Modern Drug Discovery

Amino acids have played a crucial role in therapeutics since the beginning of modern drug discovery, being present in many natural products viz. peptides (insulin) and antibiotics (vancomycin and bacitracin) among others. Amino acids are the fundamental integral parts of modern medicinal chemistry and are progressively becoming the prominent entities in novel drugs due to the three expanding trends, the urge to ‘escape from flatland’, the developing acceptance of peptides and modified peptides as therapeutics [29,30], and the growing commercial accessibility and ease of the synthesis of a broad range of amino acids with distinct side chains. There is presently no other readily accessible building block which consists of two orthogonal functional groups which are able to be altered by appropriate chemistry, such as amidation, alkylation and acylation, having one or two supplemental diversity elements directly linked to the similar chiral centre, displaying all elements in a compressed chiral configuration [31]. On the other hand, peptides are the naturally occurring substances that are found in all living organisms, including plants and animals. However, endogenous peptides exert antimicrobial and antibiotic properties and they also have enough potential to cure a variety of diseases [32]. Peptides have been studied for nearly 40 decades and a wide range of biological activities have been reported so far. In certain cases, a specified peptide exhibits more than one activity and is called a promiscuous peptide. Amino acids/peptides play essential roles in most physiological and biological functions, being involved in respiration, metabolism, digestion, immune defence, reproduction, sensitivity to pain, etc. [29,33]. Additionally, peptides also display remarkable effectiveness, tolerability and safety. Due to their intrinsic properties, peptides are an ideal starting point for developing new therapeutic agents [34].

Peptide drugs are the significant key players in pharmaceuticals with more than 50 available commercial drugs and an estimated market value of USD 13 billion as of 2010 [35,36]. This is a comparatively small portion of the global pharmaceutical industry, despite the wide range of biological functions exhibited by this class of compounds. This discrepancy originates by a few inherent restrictions of the peptides in drug development process [35,37,38,39], including a very short lifetime in the stomach, plasma and intestine, which generally result in lesser pharmacokinetic profiles. The high potency, specificity and very small levels of toxicity of some peptides, along with advancements in drug transportation, have directed to a renaissance in the field of peptide-based drugs [40,41,42]. Currently, there are dozens of peptide medications in clinical development in a wide range of therapeutic areas [43]. Peptides exhibit excellent pharmacological promise as therapeutic agents and diagnostics in numerous clinical areas, such as oncology, obstetrics, urology, endocrinology, etc., and as effective excipients in drug transportation systems to overcome cellular membrane barriers and tissues. From the most primitive microorganism to humans, peptides are essential to life. In human beings, peptides play a variety of roles, which include mucosal defence, neuromodulation and hormone regulation, etc. [44].

**Figure 2:**
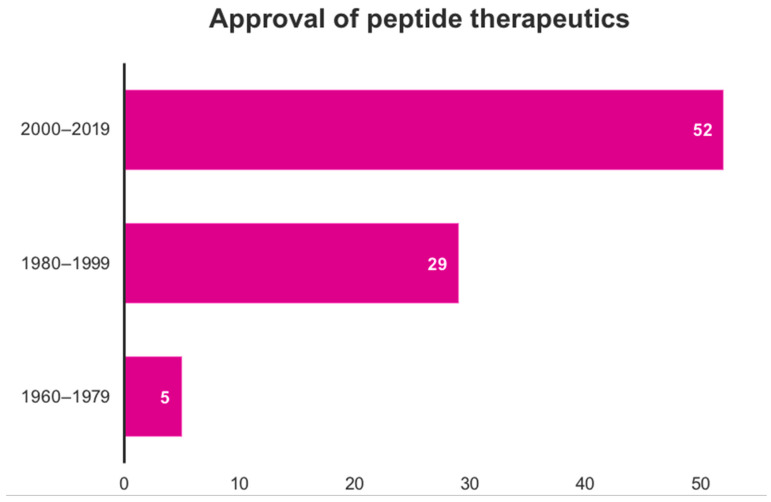
Increasing trend in peptide-based drugs.

**Figure 3:**
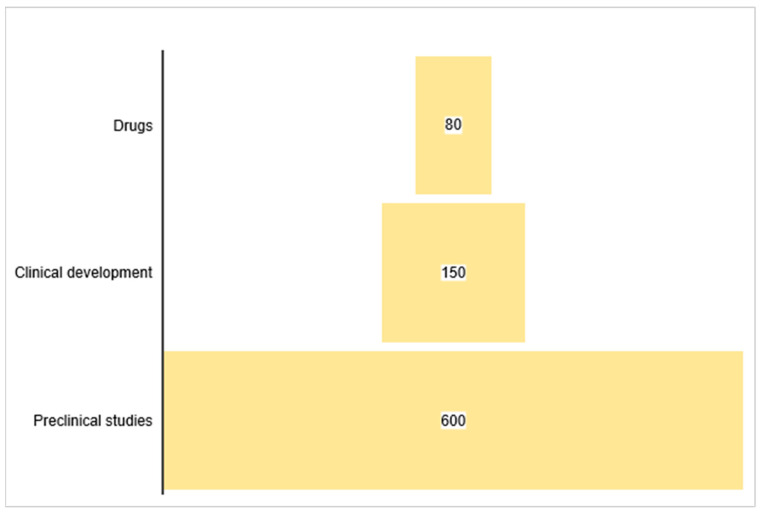
Total number of peptide-based drugs in different stages of drug discovery and development.

The first clinical use of insulin, which was derived from animal pancreas, established the discipline of peptide therapies in 1922 that revolutionised the therapy of type 1 diabetes. Several decades have passed since the entry of synthetically manufactured peptide hormones into the market, such as vasopressin and oxytocin. Industrial groups such as Charles Huguenin at Sandoz and Robert Schwyzer at Ciba stepped into the field and increased financial interest in peptides as therapeutics. Earlier, peptide synthesis by solution-phase chemistry needed months to years of effort, and it took the innovation of solid-phase peptide synthesis (SPPS) by 1963 [45], in association with the advancement of purification techniques such as HPLC, to entice considerable interest by the pharmaceutical industries. These advancements took place at the time of the golden age of small-molecule pharmaceuticals, i.e., from 1970 to 1980, during which the approval of 20 commercial orally available novel drugs per year was the benchmark [46]. However, the usage of peptides as the subtype-selective probes, which helps in the receptor studies and their continued existence as the lead molecules, constituted the fundamentals for the development of a second wave of peptide medication in the late 1980s, backed by biotechnology companies and venture funds. The lucrative success of human insulin synthesised by recombinant technology, which was approved in 1982, and synthetic gonadotropin-releasing hormones such as leuprolide and goserelin, which was approved in 1985 and 1989, proved that the existence of the peptide drug market, promotion in the drug transportation technology, formulation and synthesis, strengthened the investment as well as research. Therefore, the total number of peptides preceding into the clinical trials from 2000 to 2010 were nearly twice than that of the 1990s [47]. As of now, there are nearly 80 peptide drugs available in the global market (Figure 2), and exploration into novel peptide therapeutics is advancing with a steady pace, along with over 150 peptides in the clinical development and 400–600 peptides going through preclinical studies (Figure 3) [29,48].

**Figure 4:**
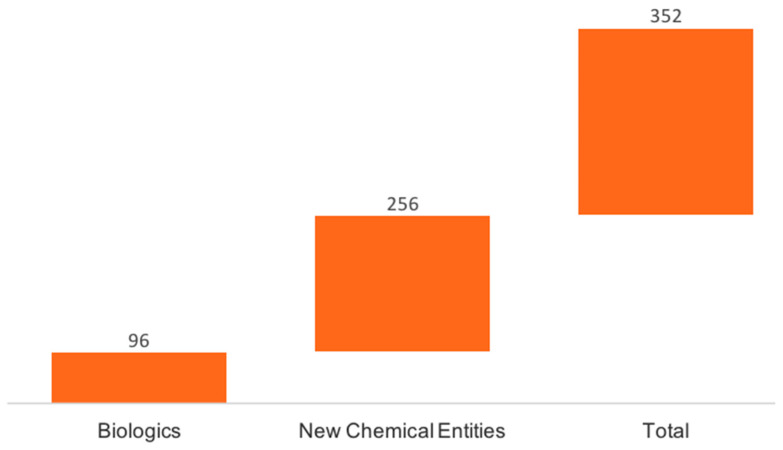
The US FDA approved drugs since 2014.

The field of peptide drug discovery has shown a great deal of dynamism in recent times, with numerous academic groups researching on this topic and the invention of novel peptide-based companies, along with the consolidation of the peptide market by the so-called big pharma [48,49,50,51]. Since 2014, the US FDA (Food and Drug Administration Department) has approved a total of 352 novel drugs, among which 96 are biologics and 256 are new chemical entities (Figure 4) [52].

**Figure 5:**
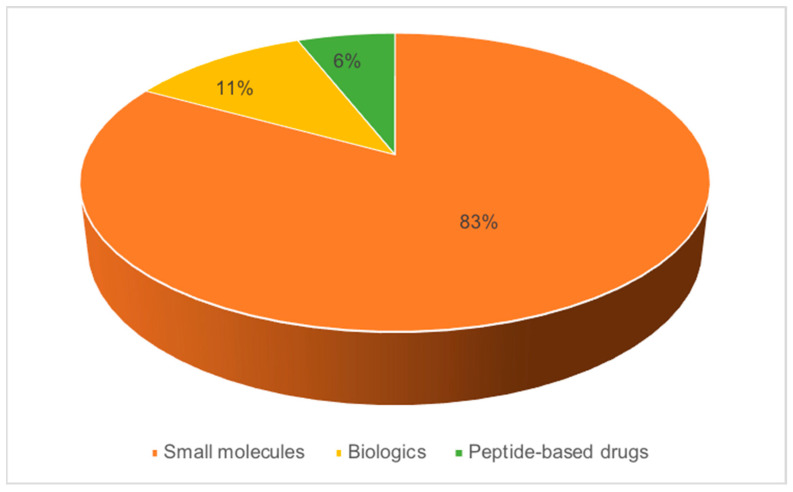
2022-based global pharmaceutical market.

**Figure 6:**
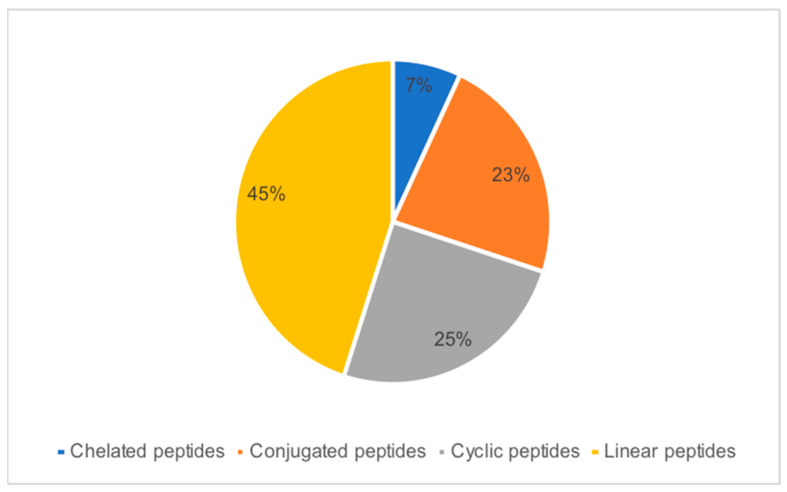
Different categories of peptide-based drugs.

Between biologics and small-molecules, peptide drugs possess an exceptional pharmaceutical space and make up approximately 6% of the total pharmaceutical market worldwide (Figure 5). Further, we can observe an average of 7.7% growth rate in the approval of peptide drugs over the course of the past six decades (Figure 2) in world peptide therapeutics industry [53]. The most usual targeted indications of peptide drugs are related to oncology, metabolism and endocrinology. Along with the above mentioned applications, peptide drugs also play a significant role in biomaterial science and drug delivery and also possess widespread applications in the medicinal domain. In this context, peptide-installed therapeutics have great priority in the pharmaceutical field [54,55]. Moreover, in order to treat various diseases, different kinds of peptides are doing rounds in the market, namely chelated peptides (7%), cyclic peptides (25%), linear peptides (45%) and conjugated peptides (23%) (Figure 6).

### 1.4. Peptidomimetics: A Promising Approach for Peptides-Based Therapeutics

**Figure 7:**
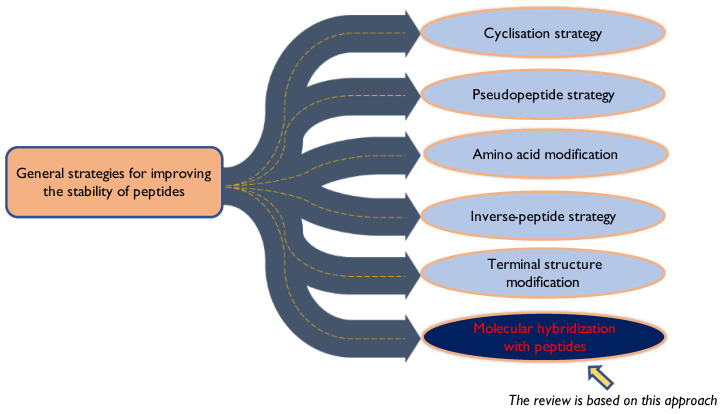
Different strategies used in peptidomimetics.

Peptides typically display certain limitations that inhibit their future clinical uses, such as low distribution, poor absorption, rapid degradation by proteases and multiple off-target interactions. Hence, peptidomimetics with better receptor-binding affinity, good bioavailability and increased metabolic stability have gained a great deal of attention [56,57]. The transformation of peptides to peptidomimetics having drug-like properties is a strong and well-established route in the advancement of novel anti-infectious drugs, aiming for both old and new biological targets. The new medicinal chemistry strategies for the development of novel peptidomimetics include structural modification strategies, such as terminal structure modification, pseudopeptide strategy, amino acid modification, inverse-peptide strategy, cyclisation strategy and molecular hybridisation with peptides (Figure 7) [58].

#### 1.4.1. Structural Modification Strategies for Developing Peptidomimetics

Peptidomimetics are an essential class of compounds and are the key elements (pharmacophores) modified to mimic natural proteins or peptides in 3D space. They withhold the capability to interact with targets, thereby generating a biological effect [57,59]. The various approaches available for turning peptides into peptidomimetics are briefly discussed herein.

#### 1.4.2. Terminal Structure Modification

The free C- and/or N-terminal peptides are naturally vulnerable to all kinds of protease enzymes by restricting their in vivo curative potential and finally limiting their usage [56,57,60]. The approach of utilising a terminal capping group capable of terminating helices is a successful strategy to minimise the peptide bond sensitiveness towards proteolytic cleavage, thereby enhancing the affinity [61].

#### 1.4.3. Pseudopeptide Strategy

The pseudopeptide approach involves the substitution of amide bonds with their respective isosteres at particular points which are not involved directly in the molecule’s biological function. It is among the most popularly used strategies for the improvement of in vivo proteolytic stability of peptides [62]. A wide range of various amide bond isosteres such as triazoles, thioamides, phosphonamidites, sulphonamides, hydroxy ethylamine and hydroxy ethylene have been proposed [63].

#### 1.4.4. Amino Acid Modification

It involves the substitution of L by D-amino acids. D-amino acids are unusual in humans and there are very few enzymes which are capable of hydrolyzing their amide bonds [33,62]. The inclusion of unnatural amino acids into peptidomimetics or peptide analogues is another kind of amino acid modification which enhances the resistance towards aminopeptidase degradation [31,64,65]. Additionally, unnatural amino acids exhibit enhanced oral bioavailability in comparison to the natural amino acids [66].

#### 1.4.5. Inverse-Peptide Strategy

The retro-inversion of amide bonds, viz. retroisosterism, is a popular strategy for the protection of amide-containing compounds from quick degradation [57]. The inverse-peptide strategy involves the retro-inversion from N→C to C→N, which improves in vitro stability by not altering the amide bond topology and geometry [60].

#### 1.4.6. Cyclisation Strategy

Cyclic peptides benefit from enhanced target affinity, increased cell permeability and resistance to the hydrolytic exercise of peptidases in comparison with their non-cyclic counterparts [67,68,69]. Moreover, the cyclic peptides are capable of inhibiting certain demanding targets such as protein–protein interactions. Therefore, developing conformationally reduced, yet powerful peptidomimetic molecules through the cyclisation of linear peptides is currently an interesting approach [56,57].

#### 1.4.7. Molecular Hybridisation with Peptides

Molecular hybrids along with peptides are an important category of unique chemical substances containing peptides and molecules [70]. Along with the positives of overcoming peptides’ limitations while retaining the molecules’ drug-like characteristics, the peptide-based molecular hybridisation has been proposed as a promising technique. The hybrid molecules can be developed by structural integration (fusing or merging) or by linking approach (presence of a linker moiety) [58].

### 1.5. Bioactive Molecules—Much Acclaimed Structures in Drug Development

Bioactive molecules are very important in the nutraceuticals and pharmaceutical industries, and they exhibit different biological activities, such as cardioprotective, neuroprotective, antidiabetic, antifungal, antimicrobial, anticancer, antioxidant, anti-inflammatory, antitubercular, antithyroid, anthelmintic, rodenticidal, insecticidal, herbicidal, plant growth regulator properties, etc. [71,72,73,74,75,76]. Bioactive molecules, including proteins and small molecules, play an important role in controlling the micro environment in vivo [77]. Bioactive molecules are very effective against microbial as well as parasitic infections [78]. Today, most of the bioactive molecules have been marketed chemically or recombinantly [79].

#### Heterocycles—A Most Promising and Diverse Class of Bioactive Molecules

Heterocyclic compounds are common structural entities in marketed pharmaceuticals as well as in medicinal chemistry and drug discovery processes. The structures of more than 80% of the superior small molecule pharmaceuticals by US retail sales had at least single heterocyclic fragment in their frameworks [80]. Heterocyclic components are present in the skeletal frameworks of all top ten brand name small molecules.

The sole reason behind such great prevalence is that the presence of heteroatoms as a part of the ring(s) in pharmaceutical molecules is highly apparent. The research process which leads to the recognition of an efficient therapeutic treatment is principally based on imitating the nature by ‘fooling’ it in an extremely subtle manner. Since heterocycles are the core elements of a broad range of natural products such as alkaloids, vitamins, carbohydrates, amino acids and nucleic acids, medicinal chemistry attempts often advance around imitating such morphological structural units. However, heterocycles perform a much greater function in the present repertoire of medicinal chemistry. Properties of certain drugs can be modulated by a strategic incorporation of heterocyclic moiety with molecules, including lipophilicity, selectivity and potency through bioisosteric replacements, aqueous solubility and polarity [81]. Thus, heterocycles play a pivotal role in the design of therapeutic agents [80,81].

Despite the extensive literature concentrated on the functionalisation and synthesis of heterocycles, there remains a significant need for further development in this domain [82] as the small molecules-based drugs suffer from serious limitations, such as high toxicity, poor selectivity, high drug accumulation in tissues and poor pharmacokinetic properties, among others. This warrants the need for novel chemical molecules that possess the properties to overcome the aforementioned drawbacks [83].

### 1.6. The Conjugation of Bioactive Molecules to Peptides—A Promising Tool in Medicinal Chemistry

**Figure 8:**
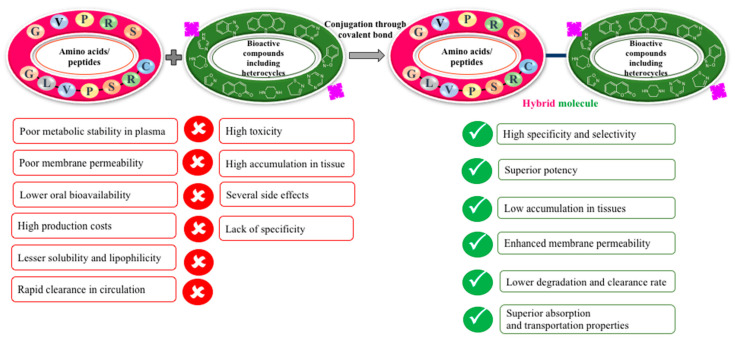
Pros and cons of amino acids/peptides and heterocycles in therapeutic development and the effect of their conjugation.

**Figure 9:**
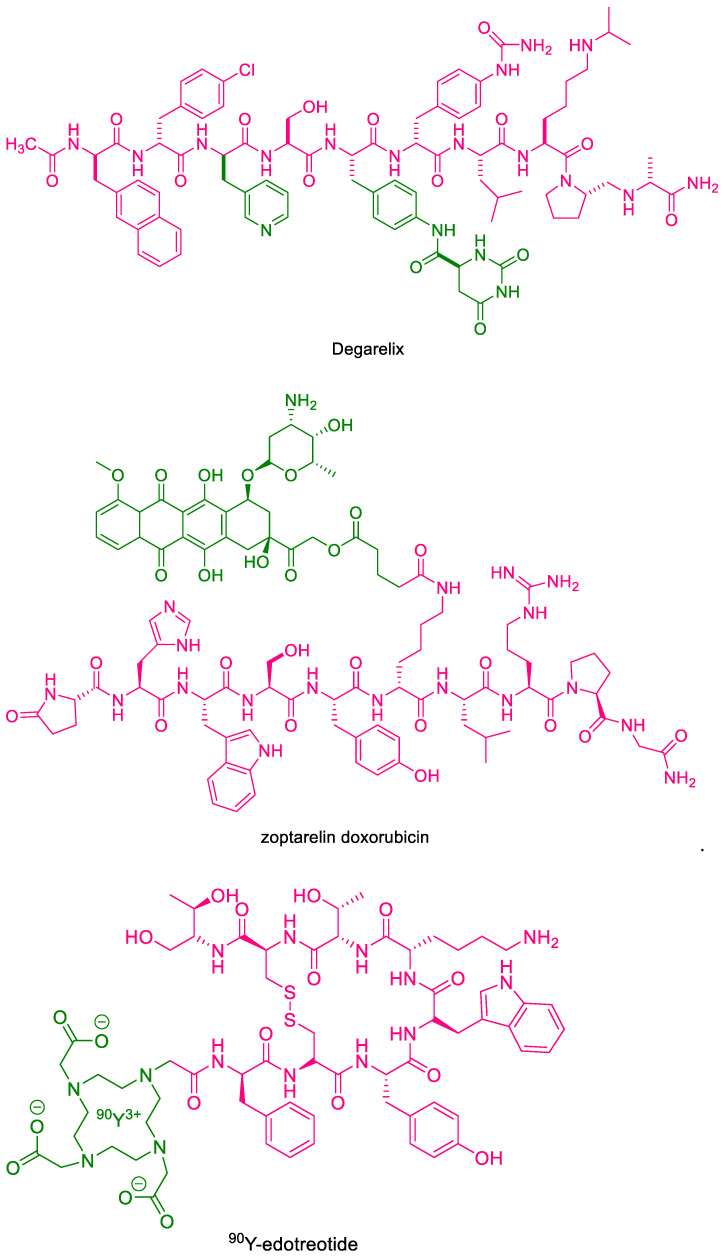
Some of the drugs in the market resulting from hybridisation of small molecules to peptides.

In the area of biomedical research, the conjugation of small bioactive molecules to amino acids/peptides is a most promising and successful method for developing new leads with greater potency. The literature also shows various spectacular evidences regarding the significance of amino acid/peptide conjugation for enhancing the selectivity, stability, high permeability and solubility of the bioactive molecule (Figure 8) [84,85,86]. These results highlight the key role played by the conjugation of amino acids/peptides with bioactive scaffolds in biomedical research [87,88,89,90,91]. As a testimony to this, there are some drugs in the market to cure certain diseases that serve as excellent examples of small molecules that have been conjugated to peptides (Figure 9).

**Figure 10:**
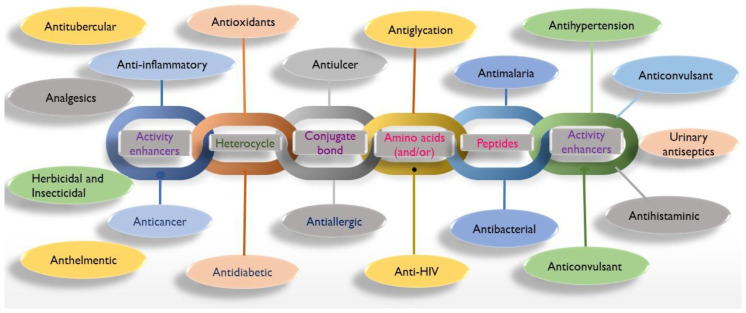
Different therapeutic applications of the conjugates.

Hybrid chemical compounds formed by the conjugation of two or more bioactive molecules have shown a wide variety of applications in biology, microelectronics, as well as material sciences [92]. Further, the conjugation of heterocyclics/bioactive molecules with amino acids/peptides results in the formation of conjugates with greater biological activities (Figure 10) and facilitates the smooth passage across cell membranes to be unleashed into the cells’ cytosol, subsequently showing greater activity. From previous records, it was found that there are several examples of amino acids/peptides conjugated to bioactive scaffolds containing lipopeptides, glycopeptides, heterocyclic-conjugated peptides and peptide nucleic acids, as well as prodrugs that showed a broad spectrum of biological activities [92,93,94,95,96,97].

**Figure 11:**
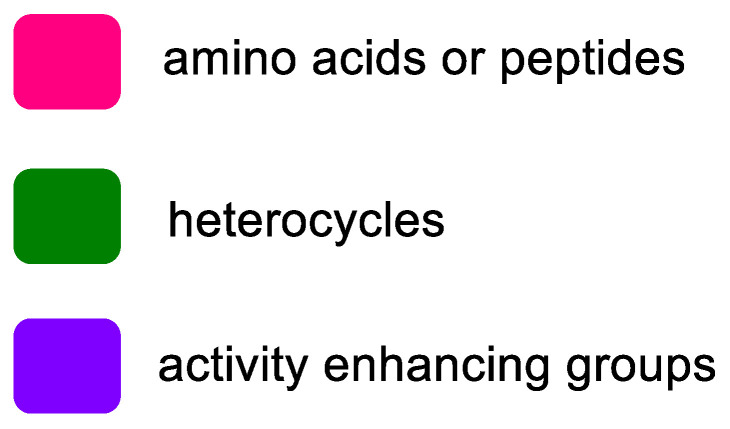
Colour coding used in this article.

In light of the above, and also considering our rich experience in developing novel therapeutics based on the conjugation of amino acids/peptides to heterocycles, we decided to present a review on the role of ‘conjugation chemistry’ in the development of therapeutics for treating infections. Based on the literature survey, it is very much apparent that conjugates of amino acids or peptides with small molecules will definitely result in sound ‘lead’ or ‘hit’ molecules in the pharmaceutical field in the near future [98]. For the sake of convenience, we have used the following colour coding throughout the article while presenting the structure(s) of the conjugates: pink colour indicates amino acids/peptides, green represents bioactive molecules (including heterocycles) and purple shows the presence of activity enhancing groups (Figure 11).

The review is divided mainly into two sections, namely conjugates of heterocycles and conjugates of bioactive molecules. Examples of the conjugates in both the sections have been arranged in chronological order according to the year of publication. In each case, we have explained the conjugates with respect to the structure/synthesis/biology/SAR and, wherever applicable, the mechanistic details as well. In this sense, we believe that this review will largely benefit the chemists and also biologists who are working in the field of therapeutics development in general and peptide chemists in particular. Thus, there may be a ray of hope for the future of the development of therapeutics for treating various infectious diseases. The foregoing sections deal with the conjugates for treating different infectious diseases.

## 2. Conjugated or Hybrid Molecules

### 2.1. Amino Acids and/or Peptides Conjugated Heterocycles

**Figure 12:**
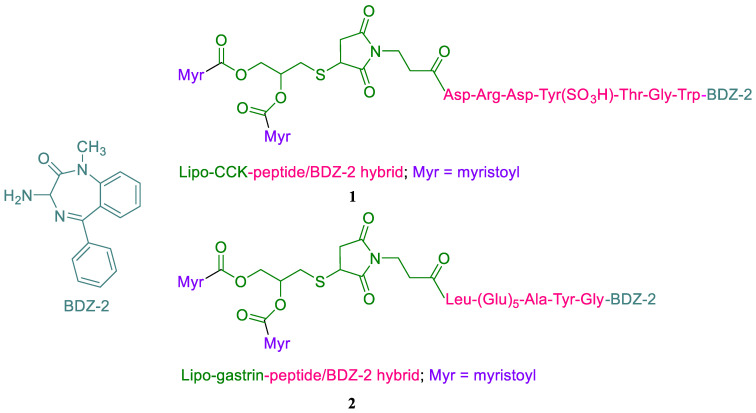
Peptides-benzodiazepine (BDZ-2) hybrid molecules.

Escherich A et al. synthesised peptides conjugated with Trp/benzodiazepine (BDZ-2) and tested their effects on CCK_A_ and CCK_B_ (Figure 12). The (neuro)hormones cholecystokinin (CKK) (**1**) and gastrin (**2**) contained a common C-terminal tetrapeptide amide sequence that was identified as the message portion, while the N-terminal substitutions were responsible for the CCK_A_ and CCK_B_ receptors. 1,4-Benzodiazipine derivatives were found to be potent and selective antagonists of CCK_A_ and CCK_B_ receptors. Based on the previous work, authors opted for dimyristoyl-type derivatives, which clearly exhibited that such lipidation led to an almost irreversible capture of di-fattyacyl-peptide analogues by cell membranes, therefore dictating receptor recognition in a membrane bound pathway. Lipo-derivatisation of the CCK/gastrin and BDZ-2 hybrids showed good binding affinities with IC_50_ of 0.74, and 1.02 µM for CCK_A_-R and CCK_B_-R, respectively. In view of these facts, authors prepared the hybrids by combining the benzodiazepine moiety with gastrin peptide as well as CCK peptides in order to check the efficacy of the conjugates as antagonists. It was observed that the binding affinities of cholecystokinin- and gastrin-peptide/BDZ-2 conjugates having tryptophan isomers showed IC_50_ values of 0.36 and 1.03 µM against CCK_A_ and CCK_B_ receptors, respectively. All the conjugates were recognised as antagonists by the CCK_A_ and CCK_B_ receptors with no appreciable increase in selectivity and avidity. Authors concluded that in the conjugates, benzodiazepine moiety occupied various binding sites and did not allow the peptide portion to interact specifically and optimally with the surface of the receptor [99].

**Figure 13:**
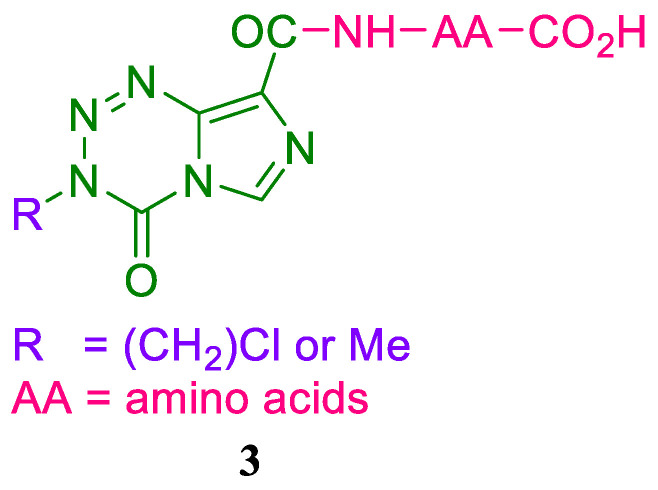
Amino acids/peptides-temozolomide and mitozolomide hybrid molecules.

Arrowsmith J et al. have performed the conjugation of various amino acids and peptides to 8-carboxylic moiety derived from the amido groups (**3**) of antitumor agents, such as temozolomide and mitozolomide, using carbodiimide as coupling agent, and subsequently developed highly potent antitumor imidazotetrazines (Figure 13). The authors further implemented the SPPS to connect the acids with DNA major groove-binding peptidic motifs which are known to adopt α-helical conformations. The connection of acids to imidazole and pyrrole polyamidic lexitropsins delivered a set of highly potent DNA minor groove-binding ligands. The in vitro antitumor activity of the synthesised conjugates was examined on mouse TLX5 lymphoma cells, which revealed that two sets of hexa- and octapeptide conjugates synthesised by using Arg, Glu, Thr, Val, Gly, Lys, Ser, Gln, Ile, His, Asp, Asn and Leu showed the same specificity towards DNA alkylations in guanine-rich tracts of DNA as temozolomide and *cis*-platin, which are major groove interactive agents. Imidazotetrazine conjugated to methoxy-substituted alanine showed good in vitro cytotoxicity against mouse TLX5 lymphoma cells with an IC_50_ value of 1.5 µM. The in vitro cytotoxicity against human Xeroderma Pigmentosum Fibroblast cells (HMGZip and HMGhAT) exhibited IC_50_ > 100 µM. It was observed that the chemical stability of imidazotetrazines was not affected by the coupling of peptides at C-8 position [100].

**Figure 14:**
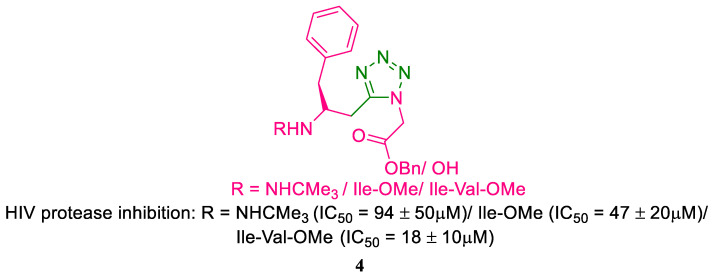
Tetrazole-based peptidomimetics.

May B C H et al. synthesised α-methylene tetrazole-based peptidomimetics (**4**) containing constrained and non-hydrolysable moieties, which were evaluated for in vitro HIV protease inhibition (Figure 14). The title compounds were prepared by incorporating α-methylene tetrazole isosteric unit into a number of peptidic sequences. The structure and conformation of the conjugates were determined by the single crystal X-ray and were found to have similarities with the isosteric core of JG-365 bound to HIV protease. The conjugates possessing Ile-Val-OMe moiety retained inhibition property against HIV protease with promising IC_50_ values of 18 ± 10 µM. The presence of a longer length of the C-terminus part enhanced the inhibition of HIV protease, and this suggested the interplay of the conjugate between the geometry of the heterocyclic moiety and the substituent at the C-terminus [101].

**Figure 15:**
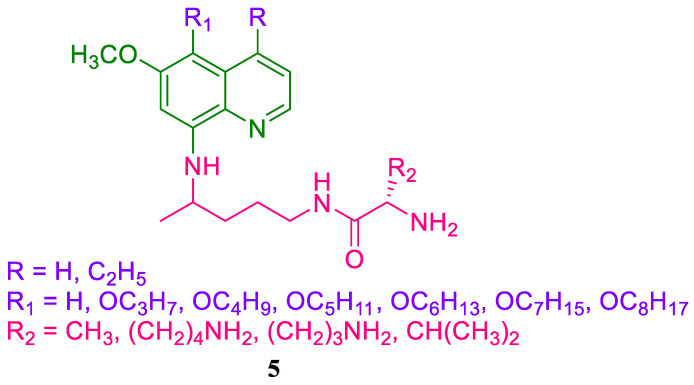
8-quinolinamine-amino acid conjugates.

Vangapandu S et al. have synthesised 8-quinolinamine-amino acid derivatives (**5**) by the conjugation of 8-quinolinamine with Val, Orn, Lys and Ala and developed them as a new class of highly potent antimalarial agents (Figure 15). Condensation of Cbz-protected *L*-amino acids with a free amino group of 8-quinolinamines in the presence of DCC, followed by hydrogenation using 10% Pd/C, yielded the desired compounds. The 28 synthesised conjugates were evaluated for in vivo blood-schizontocidal antimalarial activity against two different strains of plasmodium species, viz. *P. berghei* (drug sensitive strain) and *P. yoelii nigeriensis* (high virulent multi-drug-resistant strain) infected mice, and the results were compared with the positive control group of mice treated with chloroquine. From the results, it was observed that Ala and Val conjugates exhibited the least antimalarial property, whereas 8-quinolinamines incorporated by Lys at the side chain showed enhanced activity. Finally, the authors concluded that Lys-containing conjugate showed curative activity at 5 mg/kg in *P. berghei* at 50 mg/kg in *P. yoelii nigeriensis*, thus emerging as the most effective molecule against multi-drug-resistant strain [102].

**Figure 16:**
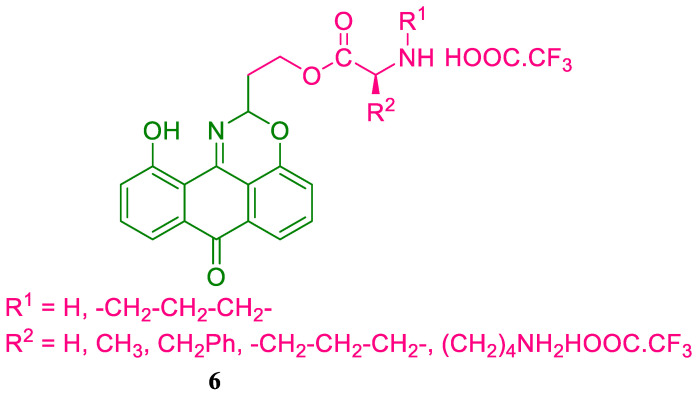
Amino acids-oxoazabenzo[*de*]anthracenes hybrid molecules.

Dias N et al. have reported the synthesis of a novel series of various Boc-protected amino acids (Gly, Lys, Pro, Phe and Ala) conjugated to oxoazabenzo[*de*]anthracenes (**6**) by the amination of carbonyl group at C-9 position of 1,8-dihydroxyanthracene-9,10-dione with 3-amino-1 propanol, followed by the oxidative cyclisation using copper (II) ions which yielded a 1,3-oxazine ring (Figure 16). The former key intermediate was treated with Boc-protected amino acids using DCC/DMAP and DCM, followed by deprotecting the Boc using TFA which furnished the title compounds. The synthesised conjugates were evaluated for DNA binding and cytotoxic studies. The structure–activity relationship studies revealed that the introduction of Lys, a cationic side-chain amino acid, strongly favoured the DNA binding interaction, whereas the neutral amino acids exhibited weaker DNA interactions, and the incorporation of hydrophobic amino acids disturbed the DNA binding interactions. From the surface plasmon resonance, fluorescence quenching and melting temperature, it was concluded that the Phe conjugates exhibited the least activity, whereas Lys conjugates were found to bind DNA with a stronger binding capacity than the other counterparts. The Lys conjugate exhibited the highest cytotoxicity with an IC_50_ value of 0.13 ± 0.06 µM against human leukaemia cells (CEM) by nearly 20 folds compared to the other compounds in the series [103].

**Figure 17:**
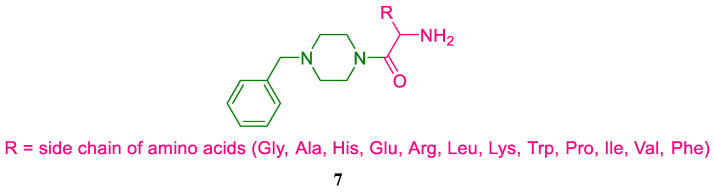
Amino acids conjugated to benzylpiperazine derivatives.

Shivakumara K et al. synthesised a new series of amino acids conjugated to benzylpiperazine derivatives (**7**) as potential antimicrobial agents by coupling various Boc-amino acids with benzylpiperazine (Figure 17). Removal of Boc-group of the intermediate using 4N HCl/dioxane yielded target molecules. All the Boc-removed conjugates were subjected to antimicrobial activity against various bacterial (including both Gram-positive and Gram-negative organisms, such as *S. aureus*, *E. coli*, *P. auregenosa* and *K. pnemoniae*) and fungal strains (*A. flavus*, *A. niger* and *F. monoliforme*) by using agar-well diffusion method. All the synthesised molecules reported enhanced antimicrobial activity, especially Trp- and Phe-containing conjugates, which reported enhanced antibacterial activity with inhibitory zone values ranging between 9 and 12 mm, which was nearly equal to that of the conventional drugs, whereas individual benzylpiperazine and amino acids were least active [104].

**Figure 18:**
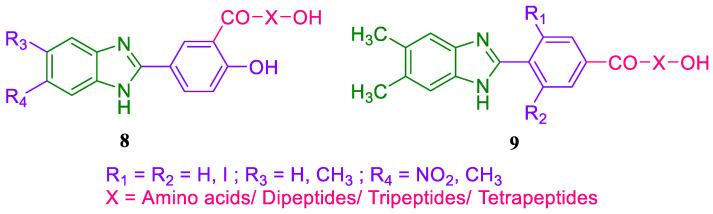
Benzimidazolo-peptide conjugates.

Dahiya R et al. have synthesised four novel substituted benzimidazolyl-salicylic (**8**)/benzoic acids (**9**) by the reaction of diazotised unsubstituted/substituted aminobenzoic acids with 5,6-dimethyl-/6-nitrobenzimidazoles in the presence of CuCl_2_, followed by coupling it with various amino acid ester/dipeptides/tripeptides/tetrapeptides to furnish new benzimidazolo-peptide conjugates (Figure 18). These were subjected to antimicrobial, cytotoxic and anthelmintic studies. The antimicrobial activity was performed using four bacterial (*E. coli*, *P. aeruginosa*, *S. aureus and B. substilis*) and fungal (*A. niger*, *T. mentagrophytes*, *M. audoouinii and C. albicans*) strains using ciprofloxacin (antibacterial) and griseofulvin (antifungal) as the reference standards. The anthelmintic activity was performed using three earthworm species, viz. *Eudrilus*, *Pontoscotex corethruses* and *Megascoplex konkanensis*, and mebendazole served as reference standard. The cytotoxic activity of the synthesised conjugates was performed on Ehrlich’s ascites carcinoma (EAC) and Dalton’s lymphoma ascites (DLA) using 5-fluorouacil as the standard drug. The biological activity revealed that hydrolysed peptide conjugates emerged as potential antimicrobial agents, especially the molecules having iodo and nitro moieties in the phenyl nucleus which showed zone of inhibition values ranging from 9 to 20 mm and MIC values of 6 to 12.5 µg/mL, respectively. In the anthelmintic activity, derivatives of Trp and His showed the highest activity. The analogues having salicylic acid group in their nucleus reported enhanced anthelmintic property along with good antifungal activity in comparison to the benzoic acid counterparts. Furthermore, it was observed that the methyl ester derivatives reported the least antimicrobial and anthelmintic activity, whereas the hydrolysed conjugates showed enhanced activity. The cytotoxicity of the title compounds was evaluated by using standard 5-fluorouacil, which revealed that only two molecules, viz. Gly-Trp-Gly-His and Pro-(nitro)Arg-containing analogues, have retained the cytotoxic property with CTC_50_ values of 6.15 µM and 114 µM for DLA cell line, while 12 µM and 84.35 µM were reported against EAC cell line [105].

**Figure 19:**
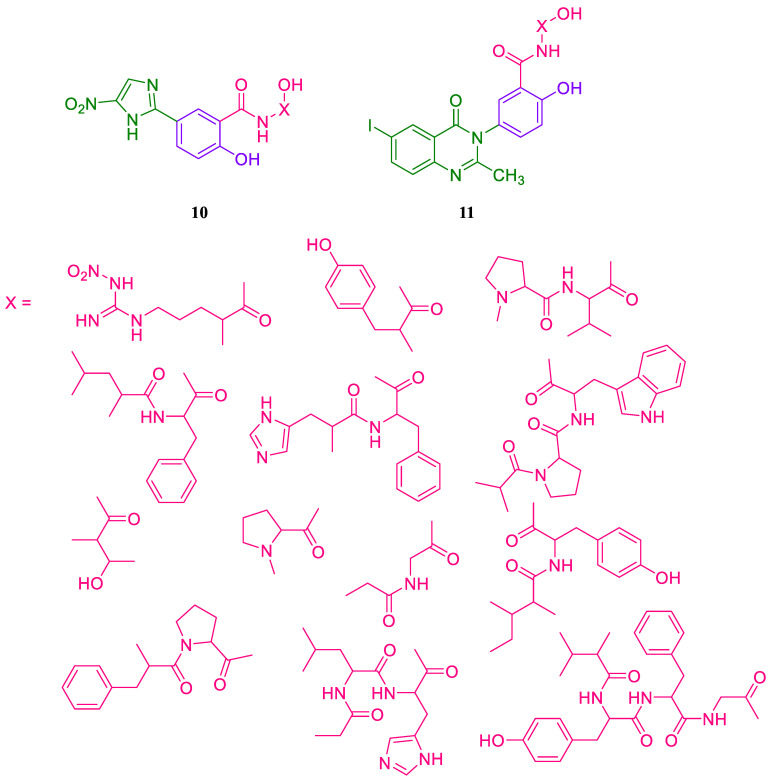
Imidazolo-/quinazolino-peptide derivatives.

Dahiya R et al. have synthesised two substituted imidazolyl (**10**)/quinazolinyl (**11**)-salicylic acids by the reaction of 5-aminosalicylic acid with 6-iodo-2-methylbenzoxazin-4-one/5-nitroimidazole, followed by the coupling of two substituted imidazolyl/quinazolinyl-salicylic acids with various amino acid esters hydrochlorides, tripeptide and dipeptide methyl esters, using TEA/NMM as base and DCC as coupling agent to yield novel imidazolo-/quinazolino-peptide derivatives (Figure 19). The synthesised analogues were evaluated for antimicrobial and anthelmintic activities. The antimicrobial activities were performed against various bacterial (both Gram-positive and Gram-negative) and fungal strains. The results revealed that synthesised analogues retained good antimicrobial activity, with zone of inhibition values ranging between 10 and 29 mm, and MIC values of 6 to 25 µg/mL against *C. albicans*, dermophytes and Gram-negative bacteria, whereas hydrolysed peptide derivatives emerged as more highly potent antimicrobial agents than their methyl ester counterparts, except for methyl ester, which retained the highest activity against dermatophytes. The anthelmintic activity revealed that highest activity was exhibited by the conjugates having Tyr and His in their molecular structure [106].

**Figure 20:**
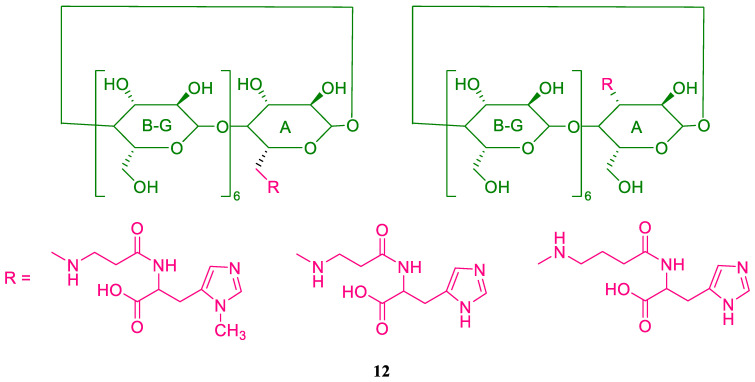
Glycosidic analogues of His dipeptides.

Bellia F et al. synthesised a new series of glycosidic analogues of His dipeptides (**12**) and evaluated their antioxidant activity and carnosinase inhibition activity (Figure 20). Anserine (β-alanyl-3-methyl-*L*-histidine), carnosine (β-alanyl-*L*-histidine) and homocarnosine (γ-amino butyryl- *L*-histidine) were conjugated with β-cyclodextrin to form various glycosides by -NH or -OH groups. The synthesised compounds played an important role in the antioxidant activity. A generation of malondialdehyde was used as a function of increasing amounts and the compounds were taken as a measure to determine the inhibitory effect towards the oxidation of human low density lipoprotein (LDL) by Cu^2+^ ions. The compound having anserine unit showed the highest antioxidant effect. The antioxidant effects of each synthesised derivative were greater than the corresponding free dipeptides and showed resistance to the hydrolysis of rat brain carnosinase. The authors felt that these results represent a good platform for further studies in animal and cellular models. The compounds with anserine derivative showed a more significant antioxidant activity than the other His analogues, which may due to the covalent linking of β-cyclodextrin residue. The anserine-containing compound showed IC_50_ of 23.4 and 41.6 µM, which was 10 to 20 folds more than other synthetic derivatives. The authors finally expressed the possible usefulness of these compounds in the treatment of neurodegenerative disorders such as Parkinson’s and Alzheimer’s diseases [107].

**Figure 21:**
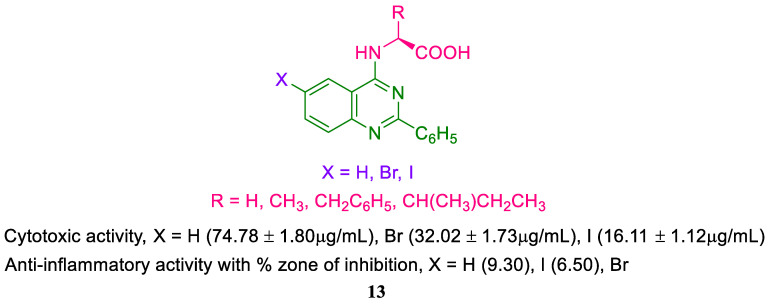
4,6-disubstituted quinazoline-α-amino acid hybrid molecules.

Chandrika P M et al. synthesised a series of 4,6-disubstituted quinazoline derivatives (**13**) conjugated with α-amino acids and then screened their anti-inflammatory and anticancer properties (Figure 21). The synthesis was initiated by the treatment of anthranilic acid with benzoyl chloride, which resulted in benz-oxazinone, upon which a treatment with aq. ammonia resulted in amides. Upon refluxing these in aq. sodium hydroxide, quinazolin-4-one was produced, which was then further chlorinated to yield 4-chloroquinazalone. The title compounds were synthesised by reacting 4-chloroquinazalone with different optically pure α-amino acids, such as Ile, Trp, methyl glycine, Phe and Gly. The anti-inflammatory activity of the compounds was evaluated by using carrageenan-induced rat paw model. Among the synthesised molecules, the compound having methyl glycine on the fourth position exhibited superior activity, with 9.3% of inhibition against a standard 48% of indomethacin. It was found that activity is inversely proportional to the length of the chain of amino acid. The anticancer activity of the synthesised compounds was evaluated against leukaemia human lymphoma cancer cell lines (U937), and it was found that the compounds exhibited lower cell viability. The conjugates having iodo and benzyl substituents showed a promising IC_50_ value of 16.11 ± 1.12 µg/mL and is close to etoposide standard [108].

**Figure 22:**
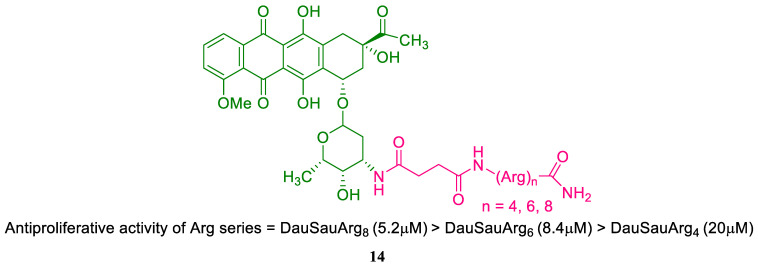
Oligoarginine-daunomycin conjugates.

Bánóczi Z et al. synthesised oligoarginine (Arg_n_, n = 4, 6, or 8)-daunomycin (succinyl derivative of daunomycin) conjugates (**14**) and evaluated their effect on leukaemia cells (HL-60) (Figure 22). Daunomycin was coupled to different lengths of oligoarginine using a succinyl spacer between the two partners. The octa- and hexa-Arg conjugates showed the highest IC_50_ values of 5.2 and 8.4 µm, respectively. All the conjugates exhibited antiproliferative activity against resistant as well as sensitive human leukaemia (HL-60) cells, i.e., DauSucArg_8_ and DauSucArg_6_ were more potent than the parent DauSuc. DauSucArg_6_ showed a lower IC_50_ value of 12.8 µm, whereas DauSucArg_8_ resulted in the highest cytotoxic effect. Fluorescence characteristics of daunomycin could be maintained even after conjugation at the Dau moiety. Hence, fluorescence intensity measurements may be used for analysing the internalisation of title compounds. Cellular uptake properties of the conjugates were performed with live cells and trypsin-treated cells. The authors concluded that increasing chain length of Arg resulted in higher cytostatic effect. The cellular uptake was partially dependent on concentration, cell types and number of Arg residues. It was found that at low concentration, only Dau was transported into the cells efficiently. Arg_6_ conjugate showed the highest internalisation, whereas conjugate of Arg_8_ showed the highest antitumor effect [109].

**Figure 23:**
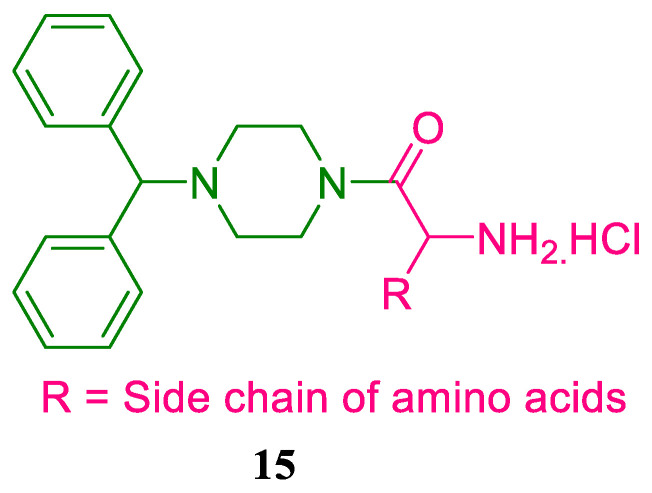
Amino acids-diphenylmethylpiperazine hybrid molecules.

Shivakumara K N et al. synthesised a novel series of antimicrobial agents by conjugating Boc-amino acids with diphenylmethylpiperazine (**15**) using EDCI/HOBt as the coupling agent and NMM as the base (Figure 23). The antibacterial activity of the synthesised compounds was tested against various bacterial strains, including both Gram-negative and Gram-positive bacteria, such as *P. auregenosa*, *S. aureus*, *E. coli* and *K. pneumoniae*. Among the synthesised compounds, molecules conjugated to aromatic amino acids, such as Phe and Trp, showed better antibacterial activity with more promising inhibitory zone values, ranging between 11 and 14 mm, than standard drugs. The molecules also showed good antifungal activity against different fungi, such as *A. flavus*, *F. monoliforme* and *A. niger* with inhibitory zone values ranging from 5 to 7 mm, respectively [110].

**Figure 24:**
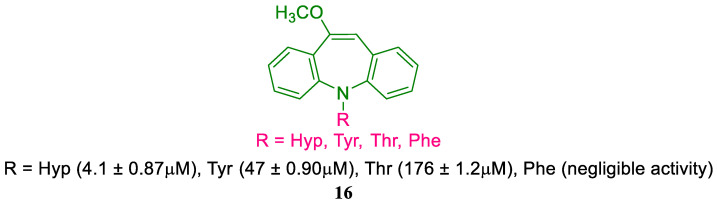
*L*-amino acids conjugated to 10-methoxy-dibenz[*b*,*f*]azepine.

Kumar H et al. have developed conjugates of *L*-amino acids (Thr, hydroxyproline (Hyp), Phe and Tyr) attached to 10-methoxy-dibenz[*b*,*f*]azepine (**16**) via 3-chloro-1-(10-methoxy-5H-dibenz[*b*,*f*]azepine-5-yl)propan-1-one intermediate, which were synthesised by N-cyclisation of 10-methoxy-dibenz[*b*,*f*]azepine with 3-chloro-propionylchloride (Figure 24). The synthesised analogues were screened for DPPH radical scavenging assay from which it was observed that the free amino acids showed the least rate of activity than the standards (BHA and ascorbic acid). On the other hand, the coupling of intermediates to amino acids led to an enhanced activity which may be due to the presence of a methoxy group in the heterocyclic system. The key intermediate did not retain radical scavenging property, but when conjugated with amino acids, enhanced activity was observed. The conjugates of Thr, Hyp and Tyr exhibited enhanced activity. The enhanced activity of Tyr conjugate was due to the presence of phenolic moiety, whereas decreased activity was observed for Phe conjugate. This may be due to the absence of a hydroxyl group. Among the synthesised conjugates, 1-(3-(5H-dibenz[*b*,*f*]azepine-5-yl)-3-oxopropyl)-3hydroxypyrolidine-2-carboxylic acid showed the highest activity with an IC_50_ value of 4.1 ± 0.87 µM/mL, which might be due to the presence of a hydroxyl group of Hyp [111].

**Figure 25:**
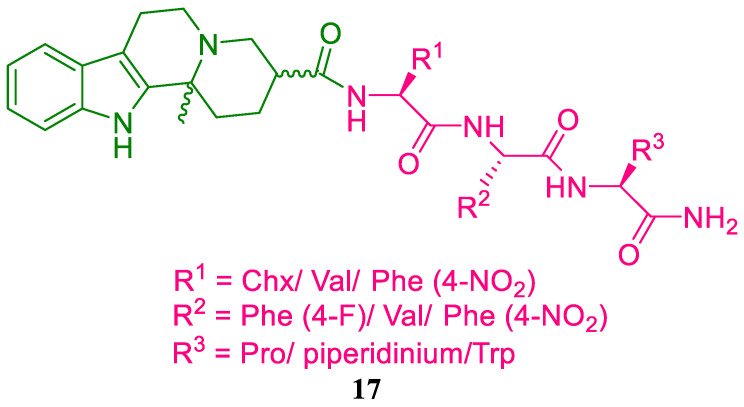
Indoloquinolizidine-peptide hybrids.

Vendrell M et al. synthesised a small library of twenty indoloquinolizidine-peptide hybrids (**17**) as agonists for dopamine receptors (Figure 25). This is made by combining two indolo [2,3-*a*] quinolizidine moieties with different tripeptides to form novel ligands for D_1_ and D_2_ dopamine receptors. These new compounds showed characteristics of multiple ligands for D_1_R and D_2_R. The first step in the synthesis is the formation of pyridinium salts (tryptophyl bromide) by reacting chiral octahydroindolo-[2,3-*a*] quinolizin-3-carboxylic acids. Then, tryptophyl bromide was reacted with methyl nicotinate in anhydrous methanol, producing desired salts. These N-alkylpyridinium salts, upon reduction, led to 1,2-dihydropyridines or 1,4-dihydropyridines, depending on their reaction condition with Na_2_S_2_O_4_/NaHCO_3_ and CH_2_Cl_2_/H_2_O, respectively. This labile intermediate was reacted with HCl in methanol to convert into cyclic tetrahydropyridine. Using Pictet–Spengler reaction, these then yielded a tetrahydro-β-carboline ring system. Further, on enamine reduction, these resulted in title compounds. The selection of the tripeptides is the main factor in the synthetic process. The synthesised compounds, mainly with trans-configuration, showed dual agonist activity at both D_1_ and D_2_ receptors. The compounds with cyclohexyl, fluorobenzyl and pyrrolidine moiety showed K_D_ values of 0.35 ± 0.03 µM and 1.5 ± 0.2 µM D_1_R and D_2_R, respectively. To evaluate the antagonist/agonist behaviour of indoloquinolizidine-peptide derivatives, they were subjected to evaluation by testing intracellular cAMP levels originated from the inhibition or activation of adenylate cyclase in cell lines [112].

**Figure 26:**
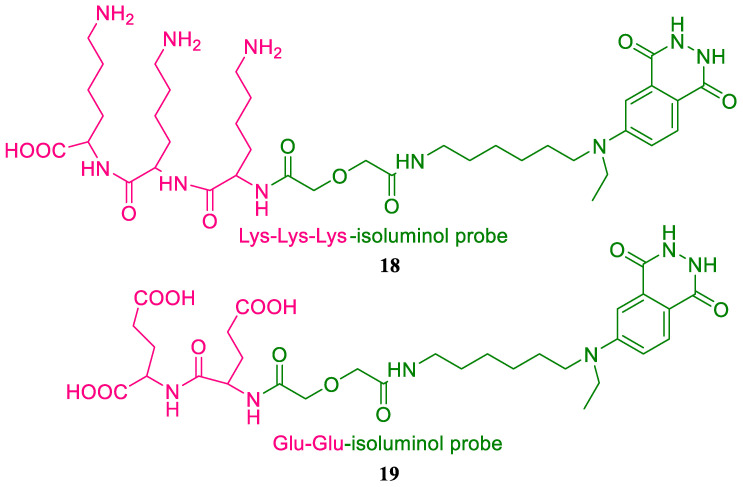
Isoluminol conjugated to amino acid derivatives.

Neelakantan S et al. synthesised novel isoluminol conjugated to Lys-Lys-Lys (**18**)/Glu-Glu (**19**) probes and evaluated their use in rapid bacterial assays (Figure 26). Initially, the isoluminol molecule was synthesised by a nine-step reaction, starting from phthalic anhydride and 6-aminohexanol under Dean–Stark condition. The synthesised isoluminol was later coupled either to Glu-Glu or Lys-Lys-Lys peptides in separate reactions using dimethoxytriazole methyl morphonium iodide (DMTMM). The Lys-Lys-Lys/Glu-Glu conjugates were tested for their ability to bind to the surface of host *L. lactis* spp. *lactis* C2 bacteria, *S. typhimurium*, *E. coli* and *S. aureus*. Fluorescence results showed that nearly 34% of the Lys-Lys-Lys-isoluminol probe was absorbed to the surface of the *L. lactis* spp. *lactis* C2 bacteria, whereas the Glu-Glu-isoluminol probe absorbed 15–20%. Specificity test showed that Glu-Glu-isoluminol probe binds more specifically than the Lys-Lys-Lys-isoluminol probe, in that it had a higher affinity for *L. lactis* spp. *lactis* C2 than for *E. coli*, *S. aureus*, or *S. typhimurium*. Finally, the authors envisaged that these types of probes have more advantages than the conventionally used polymer chain reaction (PCR) and nucleic acid probes assays [113].

**Figure 27:**
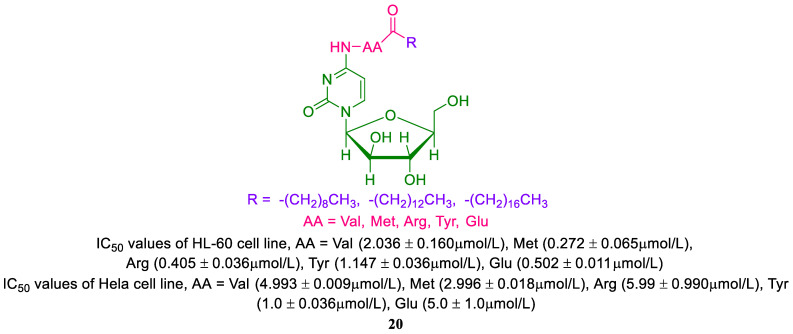
Amino acid conjugated to Ara-C (cytarabine) analogues.

Liu B et al. synthesised a series of fatty acid-amino acid conjugated to Ara-C (cytarabine) analogues (**20**) and evaluated their antitumour activities (Figure 27). Cytarabine is a drug used to treat acute non-lymphocytic leukaemia (ANLL) which showed poor lipophilicity and low bioavailability. Hence, in order to overcome these limitations, different amino acids (Val, Met, Tyr, Arg and Gln) and fatty acids (C_10_, C_14_ and C_18_) were conjugated to Ara-C. The biological activity of the resultant conjugates indicated that the lipophilicity and bioavailability of Ara-C were enhanced upon conjugation to amino acids and fatty acids. The in vitro cytotoxicity of the synthesised conjugates was evaluated against HeLa and HL-60 cells. The in vivo antitumour activity and in vitro cytotoxicity revealed that the conjugates were much more active than Ara-C in Hela cells, but not in HL-60 cells, thereby showing their cell specificity. It was found that methionine-containing derivatives, viz. C_14_-Met-Ara-C, showed best results with an IC_50_ value of 0.272 ± 0.065 µmol/L, while valine-containing derivatives, viz. C_14_-Val-Ara-C, showed the lowest activity with 32.294 ± 3.584 µmol/L of IC_50_ value against HL-60 cell line. In case of HeLa cell line, tyrosine-containing analogues, viz. C_10_-Tyr-Ara-C, reported the highest activity with an IC_50_ value of 1.000 ± 0.036 µmol/L, whereas glutamine-containing analogues exhibited the least activity with an IC_50_ value of 40.999 ± 1.999 µmol/L. It was also observed that the activity of the synthesised compounds increased as the chain length of the fatty acid increased. The authors concluded that some of the derivatives were more potent than Ara-C in mice containing S_180_ tumour, while others exhibited a lower activity in comparison with Ara-C [114].

**Figure 28:**
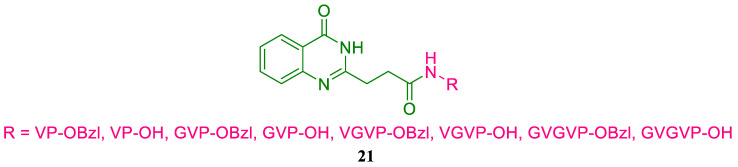
Quinazolinone conjugated to elastin-based peptide fragments.

Suresha G et al. have developed novel antimicrobial compounds (**21**) by coupling quinazolinone moiety to elastin-based fragment peptide sequences, such as VP, GVP, VGVP and GVGVP (Figure 28). In antibacterial studies, the synthesised heterocyclic conjugated tetra- and pentapeptides showed better results with inhibitory zone values ranging between 30 and 35 mm when tested against both Gram-negative (*E. coli*, *P. fluorescens*, *X. campestris pvs* and *X. oryzae*) and Gram-positive bacterial strains (*B. substilis*). The authors predicted that the enhanced antibacterial activity nearly twice that of conventional drugs was due to the polar nature of the molecules, hydrophobic properties and cationic nature of the peptides. It was also recorded that the antibacterial activity increased as the hydrophobicity and the length of the peptides increased [85].

**Figure 29:**
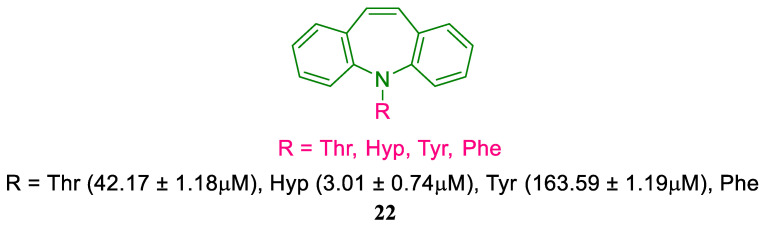
Amino acid conjugated to 5H-dibenz[*b*,*f*]azepine.

Kumar H V et al. have synthesised Hyp, Phe, Tyr and Thr conjugated to 5H-dibenz[*b*,*f*]azepine (**22**) derivatives (Figure 29). The key intermediate was 3-chloro-1-(5H-dibenz[*b*,*f*]azepine-5-yl)propan-1-one, which was obtained by N-acylation of 3-chloro propionyl chloride with 5H-dibenz[*b*,*f*]azepine upon coupling with free amino acids, which yielded 2-(3-(5H-dibenz[*b*,*f*] azepine-yl)-3-oxopropyl amino)-3-hydroxy butanoic acid, 1-(3-(5H-dibenz[*b*,*f*] azepine-5-yl)-3-oxopropyl)-3-hydroxypyrolidine-2-carboxylic acid, 2-(3-(5H-dibenz[*b*,*f*]azepine-5-yl)-3-oxopropylamino)-3-phenyl propanoic acid and 2-(3-(5H-dibenz[*b*,*f*] azepine-5-yl)-3-oxopropylamino)3-(4-hydroxyphenyl) propanoic acid. The title compounds were subjected to DPPH radical scavenging activity in which the compounds exhibited an enhanced activity when compared to the key intermediate, 3-chloro-1-(5H-dibenz[*b*,*f*]azepine-5-yl)propan-1-one, by which the authors concluded that the coupling of amino acids by elimination reaction led to an enhanced radical scavenging property. The activity of Tyr conjugate (IC_50_ = 47 ± 0.90 µM/mL) exhibiting moderate activity may be due to the presence of phenolic moiety, whereas the Phe conjugate showed decreased activity due to the lack of -OH group in the aromatic ring. The conjugation of Hyp (IC_50_ = 4.1 ± 0.87 µM/mL) showed superior activity due to the presence of -OH group on pyrrolidine ring. Finally, the authors concluded that Hyp conjugate reported the highest activity among the synthesised derivatives [115].

**Figure 30:**
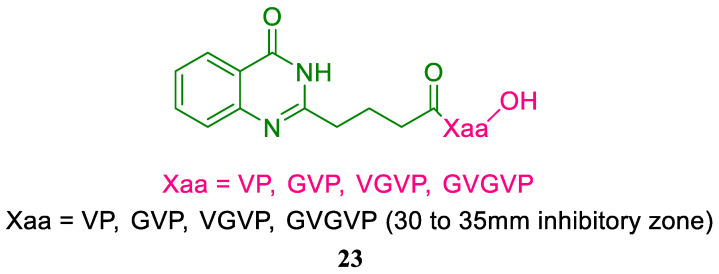
Peptides conjugated with 4-(4-oxo-3,4-dihydroquinazolin-2-yl) butanoic acid.

Suresha G P et al. synthesised novel antimicrobial compounds by coupling various peptides, such as VP, GVP, VGVP and GVGVP, with 4-(4-oxo-3,4-dihydroquinazolin-2-yl) butanoic acid to yield the title compounds (23) and further screened for antimicrobial activity against various bacterial strains, such as *X. oryzae* (Gram-negative), *X. campestris pvs*, *B. subtilis* (Gram-positive), *P. fluorescens* and *E. coli* by disc diffusion method (Figure 30). The synthesised conjugates containing GVGVP showed maximum inhibitory zone value of 30 to 35 mm against all the tested microbial strains. The peptides alone were the least hydrophobic and inactive towards the bacterial strains, but an enhanced activity even greater than that of the commercial drug was reported when the peptides were conjugated with the heterocyclic compound. It was also observed that an increase in the length of the peptide chain, variation in polarity and hydrophobicity of the molecules were responsible for increased antimicrobial activity [116].

**Figure 31:**
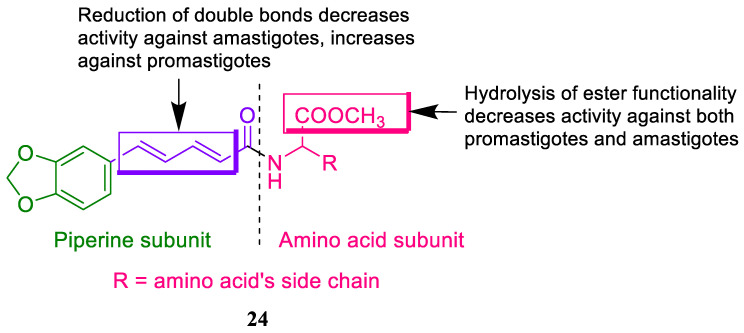
Amino acids conjugated to piperoyl derivatives.

Singh I P et al. synthesised amino acids conjugated to piperoyl derivatives (**24**) and evaluated them as antileishmanial agents (Figure 31). The authors used alkaline hydrolysis to convert piperine into piperic acid. Another method they used involved preparing piperic acid by the direct saponification of the chloroform extract of *P. nigrum*, which was treated with methanesulfonyl chloride in dry CH_2_Cl_2_ to yield piperic acid mesylate. These, upon reaction with amino acid methyl esters, resulted in piperoyl-amino acid methyl ester conjugates. All the compounds exhibited superior results for antileishmanial activity against amastigote compared to the promastigote (three-fold lesser). The title compounds with ester function showed high activity against amastigotes, whereas the compounds with acid functional group exhibited maximum effectiveness against promastigotes. Val methyl ester conjugated piperic acid displayed an IC_50_ of 0.075 mM and compounds with Met and Trp also showed good activity. The docking study was performed on the synthesised compounds by using Autodock 4.2 programme against the target adenine phosphoribosyltransferase. The compound containing isopropyl moeity showed H-bonding interaction to Ala150, Thr151, Gly152, Thr154 and Ala155 and docked efficiently in terms of location near AMP and Mg^2+^. Molecular simulation studies suggested that the synthesised compounds were bound to the active site of *L. donavani* protein 1QBB [117].

**Figure 32:**
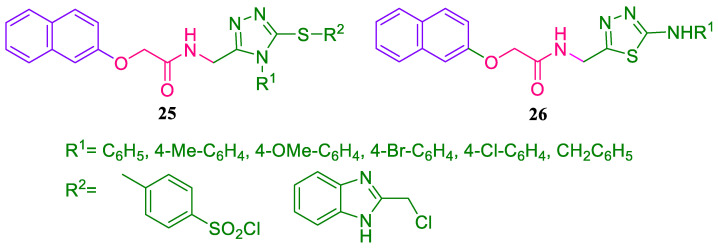
1,2,4-triazolo-naphthalene and thiadiazole analogues conjugated amino acid derivatives.

Hamad N S et al. synthesised new 1,2,4-triazolo-naphthalene (**25**) and thiadiazole (**26**) analogues conjugated to amino acid (Gly, Val, Tyr, Leu, Ala, Met and Ser) derivatives and screened for anti-HIV activity (Figure 32). Conjugates were obtained by hydrazinolysis of amino acid derivative followed by a reaction with aryl isothiocyanate in the presence of Et_3_N, which then yielded the carbamothioyl derivatives. Further cyclisation in the presence of 4N NaOH and cold H_2_SO_4_ produced 2-mercapto-1,2,4-triazole and thiadiazole derivatives, respectively. In this study, authors synthesised substituted 2-mercapto-1,2,4-triazole derivatives by also treating them with 2-chloromethylbenzimidazole or *p*-toluenesulphonyl chloride. All the conjugates were evaluated for their in vitro anti-HIV-1 (strain IIIB) and anti-HIV-2 (strain ROD), and screened by the inhibition of the virus-induced cytopathic effect in human T-lymphocyte (MT-4) cells using MTT assay. All the conjugates were inactive except for conjugates containing thiadiazole analogue with phenyl substituent and mercapto 1,2,4-triazole derivative with 2-chloromethylbenzimidazole moiety, showing EC_50_ values of 0.96 and 0.20 µg/mL, respectively. The mercapto 1,2,4-triazole derivative with 4-methyl phenyl substituents exhibited inhibitory activity against HIV-2 and HIV-1 with EC_50_ values (>25.2 and 15.2 µg/mL, respectively) and showed low selectivity with a selectivity index of 2.1 and 3.5, respectively. The substitution of naphthalene-containing amino acid precursors carrying different thiadiazole groups displayed a more superior activity than the corresponding substituted mercapto 1,2,4-triazole derivatives. The authors concluded that the mercapto 1,2,4-triazole derivative substituted with 2-chloromethylbenzimidazole could be a new lead in the development of antiviral agents as non-nucleoside reverse transcriptase inhibitors [118].

**Figure 33:**
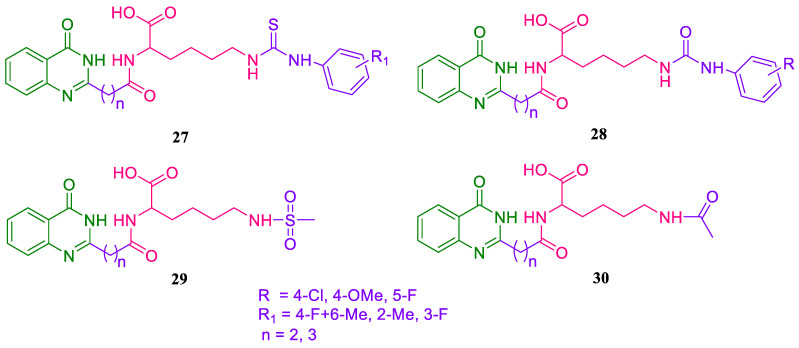
Lys-quinazolinone conjugates.

Suresha G P et al. synthesised a novel series of thiourea (**27**)/urea (**28**)/sulphonamide (**29**)/acetamide (**30**) derivatives of Lys-quinazolinone conjugates by coupling H-Lys(Boc)-OMe with quinazolinone moiety using DIEA as base and EDCI/HOBt as coupling agent (Figure 33). The Lys having ^Ɛ^amino group was further converted to substituted thiourea, urea, methyl sulphonamide and acetamide derivatives by using isothiocyanates, isocyanates, methyl sulphonyl chloride and acetyl chloride, respectively. Antibacterial studies were performed against both Gram-negative (*P. fluorescens*, *E. coli*, *X. oryzae* and *X. campestris pvs*) and Gram-positive (*B. substilis*) bacteria and it was reported that Lys-quinazolinone conjugates alone resulted in moderate antimicrobial activity, but when methyl ester protected by C-terminal was deblocked, Lys was blocked by Boc, which resulted in enhanced antimicrobial activity. Among the synthesised compounds, it was reported that thiourea and urea conjugates showed enhanced activity with substituted -F in the phenyl ring. The conjugate methyl 6-(3-(3-fluorophenyl)thioureido)-2-(3-(4-oxo-3,4-dihydroquinazolin-2-yl) propanamido) hexanoate exhibited the highest zone of inhibition values at 36 ± 1.2 mm (*B. substilis*), 38 ± 0.4 mm (*E. coli*), 36 ± 0.3 mm (*P. fluorescens*), 38 ± 0.5 mm (*X. campestris pvs*) and 35 ± 0.3 mm (*X. oryzae*). Another interesting observation was a decrease in activity as the alkyl chain of quinazolinones was increased by one carbon atom [119].

**Figure 34:**
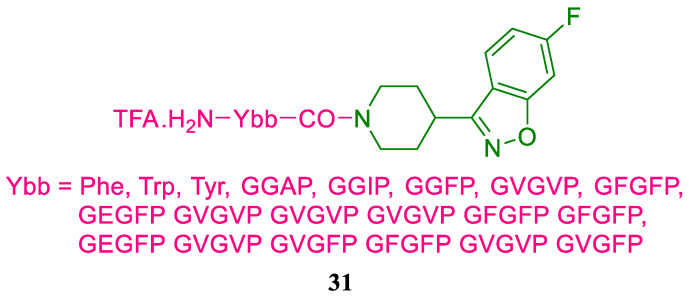
Amino acids conjugated to benzisoxazole derivatives.

Suhas R et al. developed a novel approach to enhance biocompatibility by conjugating benzisoxazole derivative with aromatic amino acids/elastin-based peptides to yield the title compounds (**31**). The elastin-based peptides, viz. tetrapeptides (GGAP, GGIP and GGFP), pentapeptides (GVGVP and GFGFP) and tricosamers (GE(OcHx)GFP GVGVP GVGVP GVGVP GFGFP GFGFP and GE(OcHx)GFP GVGVP GVGFP GFGFP GVGVP GVGFP), were synthesised by using Boc chemistry in solution-phase fragment coupling method (Figure 34). The in vitro antimicrobial studies were performed by agar-well diffusion and micro-dilution methods against various bacterial strains, including both Gram-negative (*X. oryzae* and *E. coli*) and Gram-positive bacteria (*C. staphylococcus* and *K. pneumoniae*) along with different fungal strains (*A. niger*, *F. oxysporum* and *A. flavus*). It has been reported that all the amino acids/peptides conjugated heterocycles showed enhanced antimicrobial properties (6 to 19 µg/mL) which was nearly five-fold to that of the standards, such as amoxicillin (antibacterial) and bavistin (antifungal), but heterocycles and peptides/amino acids tested alone were either the very least active or inactive towards antimicrobial activity (>50 mg/mL). In the series, conjugates of pentapeptides revealed improved activity over tetrapeptides and the amino acids used in the study. Of particular mention, conjugates of tricosamers have shown extraordinary activity by nearly two folds to that of standards with IC_50_ values ranging between 3 and 5 µg/mL against the fungal strains tested [120].

**Figure 35:**
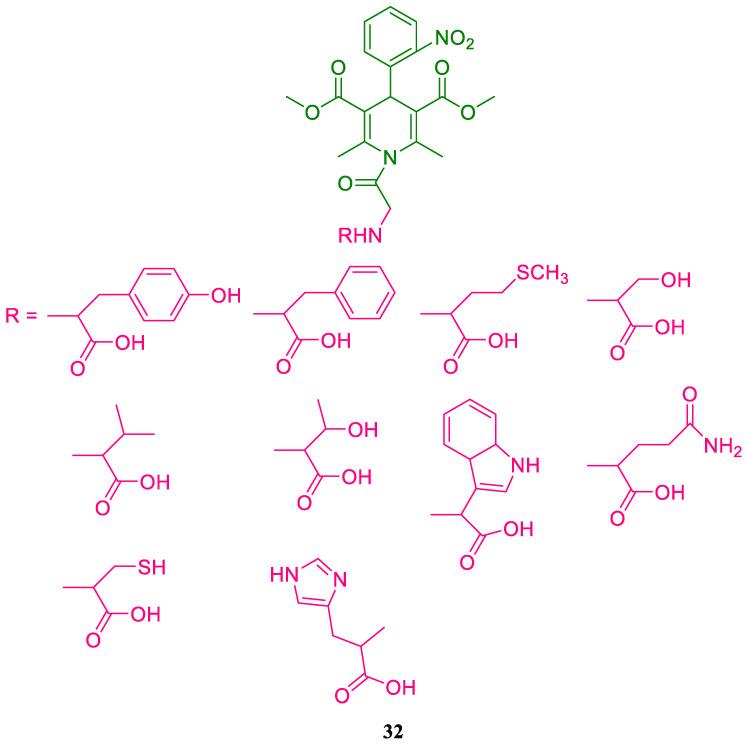
Amino acids conjugated to nifedipine.

Subudhi B B et al. synthesised a series of novel amino acids conjugated to nifedipine to yield the title compounds (**32**) and tested for their anti-inflammatory, anti-ulcer and antioxidant activities (Figure 35). The intermediate nifedipine was synthesised via Hansch pyridine synthesis by treating methyl acetoacetate with 2-nitrobenzaldehyde in the presence of ammonium hydroxide and ethanol. Nifedipine was then treated with 3-chloroacetyl chloride in the presence of triethylamine, followed by reacting it with amino acids in the presence of K_2_CO_3_ in methanol to yield the title compounds. The antioxidant activity of the synthesised compounds was tested using DPPH. The antioxidant activity of nifedipine was increased by amino acid conjugation, as indicated by the reduced IC_50_ values of the compounds. The antioxidant activity of the title compounds with Thr showed highly potent activity, indicating their good radical scavenging potential. All the synthesised compounds exhibited better activity than the standard ascorbic acid. The anti-inflammatory activity of the synthesised compounds was significantly reduced by lowering paw volume (*p <* 0.001). The synthesised compounds were evaluated for anti-ulcer activity by pyloric ligature-induced gastric ulcers in rat model, which mainly involves the reduction in acid secretion (*p <* 0.001). Compounds containing Met, Phe and Thr showed high anti-inflammatory and anti-ulcer activities. The radical scavenging property of Phe and Thr conjugates showed enhanced property with promising IC_50_ values of 10 and 65 µg/mL, respectively. This study suggested that adding amino acids to nifedipine boosted its antioxidant activity and also enhanced its antisecretory properties and anti-inflammatory and anti-ulcer activities. Phe and Met conjugates of nifedipine showed the greatest activity in the series [121].

**Figure 36:**
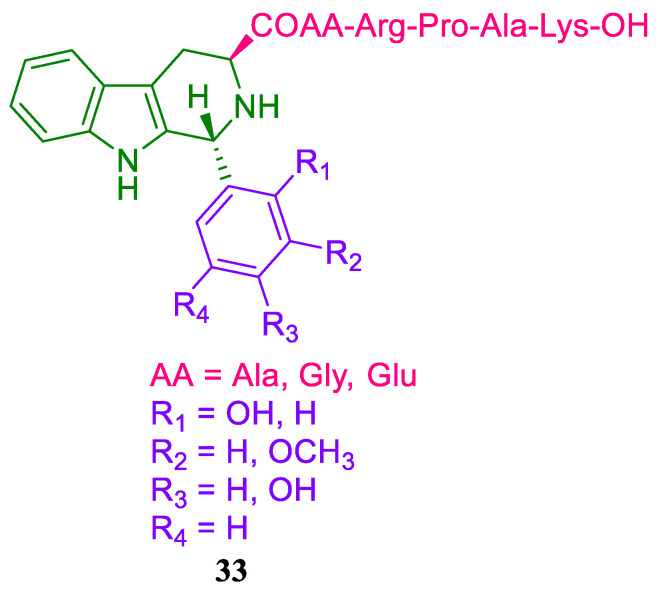
β-carboline alkaloid-peptide conjugates.

Bi W et al. have developed novel β-carboline alkaloid-peptide conjugates (**33**) possessing thrombolytic and free radical scavenging activities which can be used as an effective remedial for the treatment of acute limb ischemia/reperfusion injury (Figure 36). Initially, the β-carboline alkaloids were synthesised by the reaction of vanillin, anisaldehyde and salicylaldehyde with Trp in the presence of acidic-aqueous solution to generate a key intermediate, viz. 1,3-disubstituted tetrahydro-β-carbolines, through the Pictet–Spengler intramolecular cyclisation of Schiff base intermediate. The successive treatment of the above key intermediate with Boc-N_3_ yielded *cis* and trans-isomers of N-Boc-protected 1,3-disubstituted tetrahydro-β-carbolines. Furthermore, *L*-Lys(Z)-OBzl was prepared by implementing solution-phase peptide synthesis in which the Boc moiety was deprotected to yield intermediates having free N-terminus, followed by coupling it with trans-1,3-disubstituted tetrahydro-β-carbolines to yield protective peptide conjugates. The removal of all the temporary protecting groups in the peptide moieties yielded the title compounds. The synthesised conjugates were evaluated for various biological activities, such as in vivo thrombolytic activity, free radical scavenging activity, stability of in vitro trypsin intervention, limb ischemia-reperfusion injury, lipid peroxidation-glutathione (GSH) and malondialdehyde (MDA) assays, histological analysis and simulation of the molecular dynamics. The in vivo thrombolytic activity revealed that the synthesised amino acid conjugates could prevent the thrombus mass remarkably. The authors suggested that the conjugates exhibiting high potential thrombolytic activity also showed enhanced free radical scavenging activity against reactive oxygen species, chiefly hydroxyl radicals, which are produced by the Fenton reactions. The stability of in vitro trypsin intervention studies revealed that the depletion of the synthesised amino acid conjugates was noticed after the hydrolysis (enzyme promoted) which was delayed for nearly 3–4 h. The limb ischemia-reperfusion injury was studied on the injured limb of the rat induced by rubber band tourniquet model in which the complete limb ischemia was ensured by seizing the collateral blood flow around iliac and femoral arteries, and the results revealed that certain synthesised molecules could potentially fit as remedial for local and remote organ injury. The molecule responsible for the limb ischemia-reperfusion injury was further studied for its role on lipid peroxidation-MDA and GSH assays, which showed that the β-carboline alkaloid group was responsible for the strength of the compound’s antioxidant property. The histological analysis was performed on the skeletal muscles of sham rats, which revealed that the title molecules showed little inflammatory (0.6 ± 0.1 nmol/mg) cell infiltration with mild edema in tibialis anterior muscle, while there was no evidence reported by muscle fibres for the discernible histological changes. The molecular dynamic simulations of the synthesised β-carboline alkaloid-peptide conjugates revealed that the antioxidant capacity correlated with various parameters, such as -OH bond dissociation energy, difference between the heat of the formation of antioxidant and its radical, net-charge and HOMO energies. It was revealed that the larger the HOMO energy, the higher is the antioxidant activity. Finally, the authors concluded that the coupling of active peptide groups resulted in the most active conformation, which led to enhanced conformational rigidity and the stability of the conjugates towards trypsin, along with expanding their bioavailability [122].

**Figure 37:**
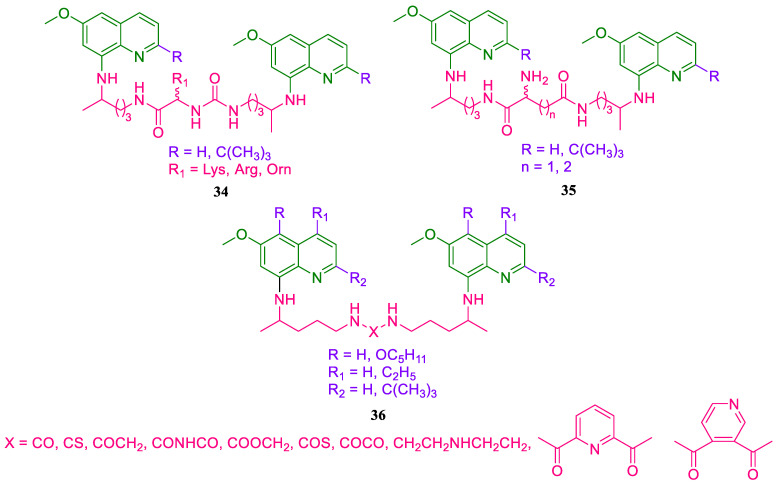
Amino acids/peptides conjugated *bis*-8-aminoquinolines.

Kaur K et al. have synthesised three novel series of *bis*(8-aminoquinolines) by coupling Orn, Arg, Lys, Asp and Glu to 8-aminoquinolines (**34**–**36**). The first series of conjugates (**34**) were synthesised by the reaction of primaquine (PQ) or 2-*tert*-butylprimaquine with protected *D*/*L*-amino acids in presence of DIC, which readily yielded side-chain- and α-NH_2_-protected amino acid analogues, followed by deprotecting Fmoc/Boc under basic or acidic conditions which led to the formation of key intermediates (Figure 37). The synthesised key intermediates, upon a coupling reaction with primaquine or 2-*tert*-butylprimaquine in the presence of CDI, resulted in protected *bis*(8-quinolinmaines) followed by the removal of side-chain amino-protecting moieties by hydrogenolysis or acidolysis that furnished the desired *bis*(8-quinolinmaines). In the second series (**35**), Glu or Asp coupled with 8-aminoquinolines via α-CO_2_H group led to the formation of intermediate products. Another molecule of 8-aminoquinolines was linked to the side-chain β/γ-CO_2_H moiety of the intermediate product. In the final series (**36**), the authors reported a one-pot synthesis of the title compounds by the covalent attachment of primaquine, 2-*tert*-butylprimaquine and (4-ethyl-5-pentyloxy)primaquine via the linker at a side-chain primary amino group that led to 8-aminoquinalones, which were treated with different electrophiles, such as chlorocarbonylsulfenyl chloride, oxalyl chloride, chloromethyl chloroformate and N-(chlorocarbonyl)isocyanate, in the presence of anhydrous THF or excess TEA or DCM that yielded the final series of conjugates. The synthesised molecules were evaluated for antimalarial, cytotoxicity, β-hematin inhibitory, methemoglobin formation, antileishmanial and antimicrobial activities. The in vitro antimalarial activity was performed using plasmodial LDH assay using two strains of *P. falciparum*, viz. chloroquine-sensitive (D6) strain and chloroquine-resistant (W2) strain, which revealed that the first series of conjugates were found to be active against both the strains, especially the molecule substituted by C(CH_3_)_3_ and D-Orn, which showed the highest activity with IC_50_ values of 0.30 and 0.34 µg/mL against W2 and D6 strains, respectively. The second series of conjugates were comparatively less active with IC_50_ values ranging between 2.2 and 3.8 µg/mL (W2 strain) and 2.7 to 4.76 µg/mL, whereas in the case of final series, the analogue of *bis*-quinoline derivative linked via pyridine-3,4-dicarbonyl linker showed the enhanced activity with promising IC_50_ values of 0.87 µg/mL for W2 strain, and 1.5 µg/mL for D6 clone. The in vitro cytotoxicity of all the analogues were examined using mammalian kidney cell line (Vero) by natural red assay method and it was reported that none of them were found to retain cytotoxicity, which specifies the selectivity only towards the antimalarial action. The title compounds were assayed for the inhibitory activity of β-hematin by using chloroquine as the reference standard, from which it was revealed that one of the derivative of first series showed enhanced β-hematin inhibition with a promising IC_50_ value of 10.8 µM compared to the standard chloroquine, with an IC_50_ value of 80 µM. The conjugates were also examined for methemoglobin (MetHb) formation and their toxicity using PQ as reference standard, which revealed that the derivatives of first series exhibited the least MetHb formation, ranging between 0.2 and 7.9%, whereas the derivatives of second and third series showed MetHb formation of 1.55 to 13.35% and 0.25 to 20.3%, respectively. The in vitro antileishmanial activity performed against *Leishmania donovani* promastigotes revealed that in the first series, the molecule having H and *L*-Arg at R and R_1_ showed the highest activity with IC_50_ and IC_90_ values of 3.1 µg/mL and 7.2 µg/mL, respectively, whereas in the second series, the analogue having R = H and n = 1 and R = C(CH_3_)_3_ and n = 2 emerged as the most promising compound with an IC_50_ of 6.2 and 3.9 µg/mL, and an IC_90_ of 30 and 10.7 µg/mL. In the final series, three conjugates were found to be the most potent with IC_50_ and IC_90_ values ranging between 2.9 and 3.5 µg/mL, and between 7.0 and 27 µg/mL compared to IC_50_ and IC_90_ values of the standard pentamidine, i.e, 1 µg/mL and 3.8 µg/mL. The antimicrobial activities were performed against various bacterial (*S. aureus*, *E. coli*, *M. intracellulare*, methicillin-resistant *S. aureus* and *P. aeruginosa*) and fungal strains (*C. neoformans*, *A. fumigatus*, *C. krusei*, *C. glabrata* and *C. albicans*). The results revealed that two analogues of the first series possessed antibacterial activity against both the strains of *S. aureus* with a promising IC_50_ value ranging between 1.78 and 2.73 µg/mL, and none of the analogues retained antibacterial activity against *P. aeruginosa* and *E. coli* strains. In antifungal activity, analogues of all the series exhibited fungicidal activity, especially the conjugates of final series which retained the maximum antifungal activity against *C. neoformans* with a good IC_50_ value of 5 µg/mL, respectively [123].

**Figure 38:**
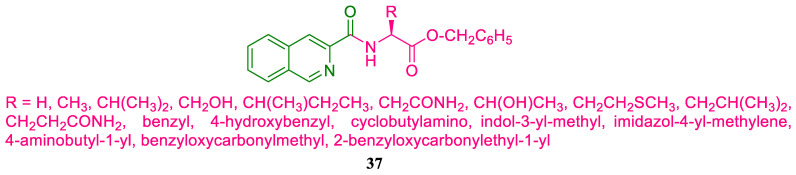
Benzyl esters of N-isoquinoline-3-carbonyl-*L*-leucine and N-isoquinoline-3-carbonyl-*L*-threonine.

Zheng M et al. synthesised novel benzyl-esters of N-isoquinoline-3-carbonyl-*L*-leucine and N-isoquinoline-3-carbonyl-*L*-threonine (**37**) by a three-step reaction and developed them as potential antitumor agents (Figure 38). The first step involves the conversion of *L*-Phe to 3S-1,2,3,4-tetrahydroisoquinoline-3-carboxylic acid via Pictet–Spengler condensation, followed by oxidation using KMnO_4_ resulting in isoquinoline-3-carboxylic acid. In the final step, the amino acid benzyl-esters of Leu and Thr were incorporated into isoquinoline-3-carboxylic acid using DCC/HOBt/NMM, and which yielded N-isoquinoline-3-carbonyl-amino acid benzyl-esters. The synthesised compounds were subjected to in vitro antiproliferation activity on 96 microtiter plates by using MTT method on the growth of Hela and HL-60 cells. The results revealed that the IC_50_ values of all the synthesised analogues inhibited the growth of Hela and HL-60 cells with IC_50_ values ranging from 87.74 to 507.94 nM and 44.50 to 99.94 nM, respectively. The in vivo antitumor activity of the synthesised analogues consisted cytarabine (100 µmol/kg/day in 0.2 mL of NS) serving as the positive control and 0.2 mL of normal saline (NS) serving as the negative control. The results revealed that the tumour weights of all the analogues-treated mice ranged from 0.821 to 1.588 g, which were significantly lesser than that of NS-treated mice, thereby concluding that, at this dose, the drug possesses antitumor activity. The authors also stated that in vitro antitumor activity of isoquinoline-3-carboxylic acid exhibited 80-fold higher potency than that of β-carboline-3-carboxylic acid. The dose dependence of the oral antitumor activity reported that the weight of the tumour in treated mice steadily increased with decreased dosage, suggesting that the title compounds presented dose-dependency to prevent the tumour growth of treated mice. The neurotoxic and acute toxicity of the highly potent analogues were determined on healthy mice, from which it was observed that the analogues exhibited low toxicity, along with zero neurotoxic behaviour, concluding as safety antitumor leads. The 3D QSAR analysis of all the molecules was performed to understand the dependence of in vivo and in vitro antitumor activity. Furthermore, the in vitro membrane permeation analysis revealed that N-isoquinoline-3-carbonyl-*L*-leucine benzyl-ester and N-isoquinoline-3-carbonyl-*L*-threonine benzyl-ester exhibited 2.5 folds higher in vitro membrane permeability, 2.6 to 21.3 folds enhanced antiproliferative activity and 4.4 folds enhanced in vivo antitumor activity than isoquinoline-3-carboxylic acid [124].

**Figure 39:**
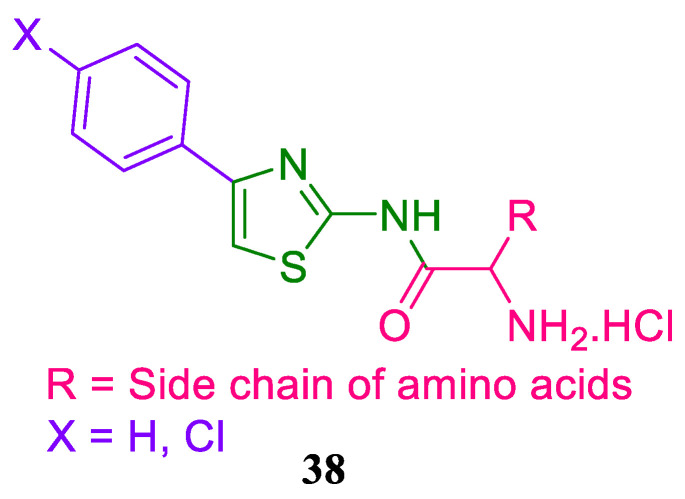
Amino acids-conjugated thiazoles.

Prakasha K C et al. synthesised two new series of amino acid-conjugated thiazoles (**38**). Boc-amino acids (Gly, Pro, Phe, Tyr, Trp, His, Ser, Lys, and Arg) were conjugated with 2-amino-4-(4′-chlorophenyl) thiazole and 2-amino-4-phenylthiazole using IBCF/NMM as the coupling reagent/base (Figure 39). By using the agar-well diffusion method, the antibacterial and antifungal activities were tested for both series, and they showed very good results in both the assays. Due to the electron-withdrawing property of the chlorine atom present at the para position of the phenyl ring in 2-amino-4-(4′-chlorophenyl) thiazole, the compound showed an increased activity compared to 2-amino-4-phenylthiazole. The order of antibacterial activity evaluated by zone of inhibition method was found to be Trp derivatives (5 to 9 mm) > Tyr derivatives (2 to 8 mm) > Phe derivatives (2 to 6 mm). Among the synthesised conjugates, derivatives of Arg and Lys showed superior antifungal activity, with zone of inhibition values of 5 to 11 mm and 4 to 8 mm, which might be due to their more lipophilic nature. Authors have suggested that the obtained results could be due to the easy passage of the thiazole conjugates through the cell wall in turn into the cytosol of fungi and bacteria, thus disrupting the metabolic activities of microbes by the thiazole derivatives [125].

**Figure 40:**
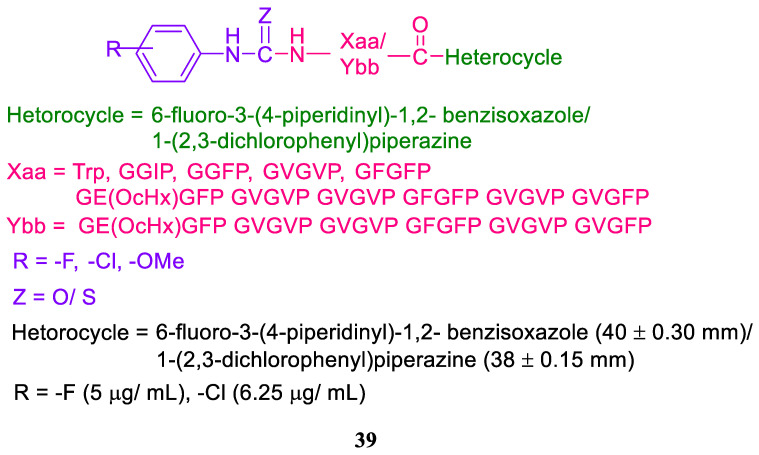
Amino acid/peptides conjugated to heterocycles.

Suhas R et al. synthesised a series of uriedo and thiouriedo analogues of amino acid/peptide conjugated heterocycles (**39**), such as 6-fluoro-3-(4-piperidinyl)-1,2-benzisoxazole and 1-(2,3-dichlorophenyl) piperazine, and evaluated their antimicrobial activity (Figure 40). The authors synthesised a small library of forty-five new derivatives of thiourea and urea. The amino acid/peptides used in the study were: Trp, GGIP, GGFP, GVGVP, GFGFP, GE(OcHx)GFP GVGVP GVGVP GFGFP GFGFP and GE(OcHx)GFP GVGVP GVGVP GVGVP GFGFP GFGFP. The results clearly showed that thiourea-containing compounds were highly active compared to ureas, which may be due to the electronegativity and larger size of S as it improves the biding affinity with the interaction sites. The compounds containing phenyl ring, namely Trp, Tyr and Phe, exhibited more antimicrobial activity than the standards. The synthesised compounds were examined for antimicrobial activity of different strains of human pathogens of both Gram-positive bacteria, such as *K. pneumoniae* and *Coagulase positive staphylococcus*, and Gram-negative organisms, such as *E. coli* and *X. oryzae*, and antifungal activity was studied against *A. niger*, *A. Flavus* and *F. oxysporum*. Bavistin and amoxicillin were used as standard drugs for antifungal and antibacterial studies, respectively. The compounds having EWGs, such as -F and -Cl, in the phenyl ring of urease/thioureas were more active with low MIC values of 3.25 to 5 µg/mL. The compounds containing piperidine moiety have shown a small increase in the activity over the piperazine analogues that may be attributed to the more lipophilic nature of piperidine moiety, along with the presence of -F and benzisoxazole. It is interesting to note that the analogues of tricosamers conjugates have shown inhibition in the growth of fungal species at 1–3 µg/mL, which is around 15–25 fold more superior than the standard [126].

**Figure 41:**
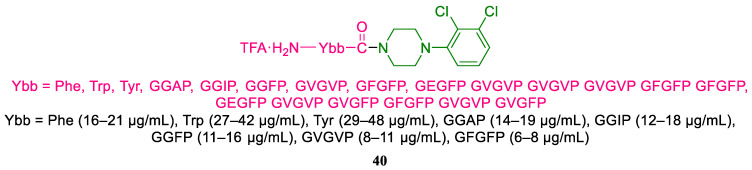
Elastin-based peptides conjugated to 1-(2,3-dichlorophenyl) piperazine.

Suhas R et al. synthesised a new series of elastin-based peptides conjugated 1-(2,3-dichlorophenyl) with piperazine analogues (**40**). In this work, the authors have used different aromatic amino acids (Phe, Tyr and Trp): tetrapeptides (GGAP, GGIP and GGFP); pentapeptides (GVGVP and GFGFP); and tricosamers (GEGFP GVGVP GVGVP GVGVP GFGFP GFGFP, GEGFP GVGVP GVGFP GFGFP GVGVP GVGFP) (Figure 41). The antifungal activity of the synthesised compounds was tested against fungal species such as *A. niger*, *A. flavus* and *F. oxysporum* by microdilution and agar-well diffusion methods. The conjugates were evaluated for antibacterial activity against various strains of pathogens of both Gram-negative organisms, such as *E. coli* and *X. oryzae*, and Gram-positive bacteria, such as *K. pneumoniae* and *C. positive staphylococcus*. Bavistin and amoxicillin were used as standard drugs for antifungal and antibacterial studies, respectively. The heterocycle conjugated amino acids/peptides have shown higher activity (6 to 19 µg/mL) than the free peptides or heterocycles alone (>50 µg/mL). The compounds with more hydrophobicity and aromaticity, such as Phe, Trp and Tyr, exhibited enhanced activity. The order of activity of the heterocyclic conjugated amino acids/peptides was found to be GFGFP (6 to 8 µg/mL) > GVGVP (8 to 11 µg/mL) > GGFP (11 to 16 µg/mL) > GGIP (12 to 18 µg/mL) > GGAP (14 to 19 µg/mL) > Phe (16 to 21 µg/mL) > Trp (27 to 42 µg/mL) > Tyr (29 to 48 µg/mL) which may be due to the length of the peptide chain, conjugation and hydrophobicity. The report showed that all the conjugates exhibited more increased activity than the standards used. Of particular interest, the conjugates of tricosamers exhibited high activity with a MIC value of 3 to 5 µg/mL against fungal species, which is five fold more than the standard, bavistin [127].

**Figure 42:**
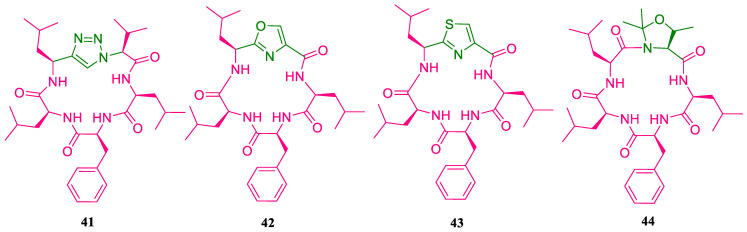
Sansalvamide A peptidomimetics.

Davis M R et al. have synthesised several sansalvamide A peptidomimetic cytotoxic derivatives comprising heterocycles, such as triazoles (**41**), oxazoles (**42**), thiazoles (**43**) and pseudoprolines (**44**), across the macrocyclic backbone (Figure 42). The synthesis of these analogues involved several approaches in which macrocyclic peptide was converted to its peptidomimetic counterparts. These approaches comprise the generation of an azide and alkyne for click chemistry by peptide modification, thiazole generation by Hantzsch reaction, the generation of pseudoproline derivatives from Thr and the conversion of Ser to oxazole. The synthesised molecules were subjected to in vitro cytotoxicity against HeLa cervical cancer cell lines. The results revealed that the incorporation of pseudoproline or oxazole to the peptide backbone reduced the cytotoxicity in comparison to the parent compound with a growth inhibition of <20% [128].

**Figure 43:**
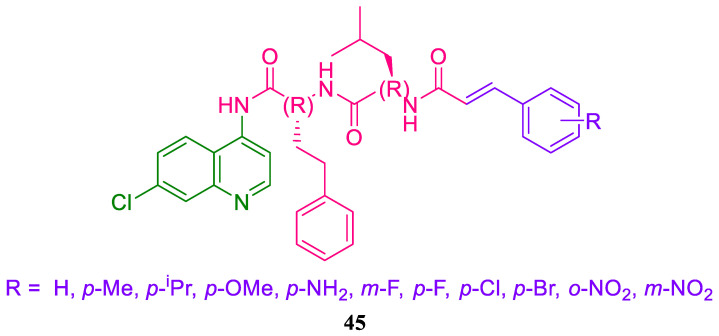
4-aminoquinoline/cinnamic acid conjugates.

Pérez B C et al. synthesised twenty-three novel 4-aminoquinoline/cinnamic acid conjugates (**45**) by linking it through a suitable spacer, viz. Leu-Phe, having non-proteinogenic amino acids for the development of potential antimalarials with dual mode of action by using various coupling agents (Figure 43). The synthesised molecules were evaluated for the inhibition of heme polymerisation, anti-plasmodial activity, in vitro inhibition of falcipain and in silico studies. The inhibition of heme polymerisation assays were performed using 96-well microplates possessing negative (water and DMSO) and positive controls (1 mM chloroquine, CQ). The assays revealed that the title compounds with phenyl ring substituted by nitrogenated groups, such as -NO_2_ and -NH_2_ at para position, -NO_2_ at ortho position and small alkyl groups (-CH_3_) at para position, reported the best inhibitory activity, whereas the presence of -NO_2_ at meta position, halogens/hydrogen at para position and bulky group at para position were found to be unfavourable for the inhibitory activity, finally inducing the complete loss of activity. It was also reported that the conjugates without spacer emerged as better falcipain-2 inhibitors. The authors finally concluded that the inhibition of hemozoin formation can be successfully achieved by the replacement of CQ’s aliphatic chain with dipeptidyl-cinnamoyl group. The molecular simulations revealed that all the conjugates were not able to fit in to the FP3 catalytic site as effectively as FP2 subsites. The anti-plasmodial activity was performed on blood-stage CQ-resistant *P. falciparum* strain W2, which revealed that molecule containing *p*-isopropyl moiety (bulky electron donating group) exhibited the highest anti-plasmodial activity, but with zero inhibitory activity of in vitro heme polymerisation [129].

**Figure 44:**
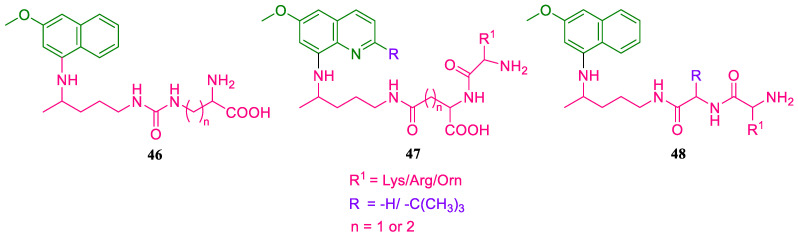
8-aminoquinolines conjugated amino acids/dipeptides/pseudopeptides analogues.

Kaur K et al. synthesised three new series of ring substituted 8-aminoquinolines conjugated with amino acids (**46**)/dipeptides (**47**)/pseudopeptides (**48**) and evaluated for in vitro antimalarial activity, cytotoxicity, antileishmanial activity and inhibition of β-haematin (BH) formation (Figure 44). From the previous reports, the authors found that 8-aminoquinolines conjugated cationic amino acids enhanced the antimalarial activity more than the anionic and lipophilic amino acids, and hence, they selected cationic amino acids such as ornithine (Orn), Lys and Arg. The 8-aminoquinoline derivatives such as primaquine and carboxyprimaquine were treated with appropriately protected *D*/*L*-amino acids in the presence of CDI, followed by deprotection using Pd-C/H2 in methanol to yield title compounds. Except for D-Arg, all the products were obtained in good yields. The effectiveness of the synthesised compounds as antimalarials was assessed against chloroquine-resistant (W2) and chloroquine-sensitive (D6) strains of *P. falciparum* by plasmodial LDH assay. The synthesised compounds were evaluated for in vitro cytotoxicity against mammalian kidney cell lines (Vero) by neutral red assay. The synthesised analogues were also screened for the inhibition of BH formation. The antileishmanial activity of the synthesised compounds were tested in vitro against promastigotes *Leishmania donavani* by alamar blue assay. The amino acid conjugates showed weak or inactive in vitro antimalarial and poor inhibition of the BH formation, and inactive against *L. donavani*. The dipeptide conjugates containing L-Lys- L-Lys and L-Arg- L-Arg and C(CH_3_)_3_ alkyl side chain exhibited superior activity with IC50 values of 0.63 and 0.40 µg/mL and IC50 values of 0.97 and 0.82 µg/mL against W2 and D6 strains, respectively. The compounds having alkyl side chain consisting -H, -H and C(CH_3_)_3_ and another consisting L-Orn, L-Lys and L-Arg exhibited high activity against *L*. *donavani*, with an IC50 of 4, 3.5 and 9 µg/mL, respectively. The compounds containing both free carboxyl and amino groups were less potent than the parent primaquine which may due to the presence of undesirable -CO groups. The pseudopeptide conjugates showed the most potent antimalarial activity with IC50 values of 0.13 and 0.26 µg/mL. The title compounds were also evaluated for in vivo blood-schizontocidal antimalarial activity by *P. berghei* mouse malarial model which showed 100% curative activity at 100mg/kg. The compounds were also evaluated for their antibacterial activity against *S. aureus*, *E. coli*, *P. aeruginosa* and *Myobacterium intracellulare* and were found to possess promising activity with IC50 of 5.9 and 7 µg/mL, MBC value of 20 µg/mL and MIC value of 10 µg/mL. The antifungal activity of the synthesised conjugates was tested against *A. fumigatus*, *C. neoformans*, *C. albicans*, *C. glabrata* and *C. krusei*. The compounds were found to be inactive against *A. fumigatus* and active against *C. neoformans*, with IC_50_ between 2.5 and 7 µg/mL. The authors concluded that the title compounds showed broad spectrum activity against various pathogenic protozoal and microbial infection [130].

**Figure 45:**
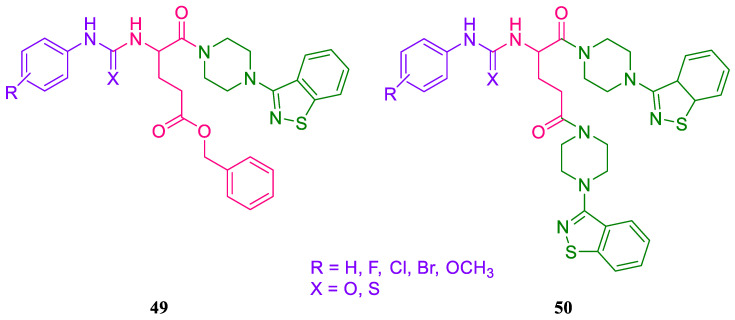
Glu conjugated to 3-(1-piperazinyl)-1,2-benzisothiazole.

Sharma A et al. reported the synthesis of a novel series of thiourea and urea derivatives (**49**–**50**) by the double conjugation of 3-(1-piperazinyl)-1,2-benzisothiazole with Glu, and further screened for urease enzyme inhibition activity, in vitro antiglycation studies, H^+^/K^+^-ATPase inhibition activity, antibacterial and antifungal activities (Figure 45). Among urea- and thiourea-derivatives, the latter were reported to have promising results in comparison to the former. It was also reported that molecules with electron-releasing groups, such as methoxy and electron-withdrawing fluoro, showed enhanced inhibitory potency with IC_50_ values ranging from 4.6 ± 0.1 to 14.5 ± 0.4 µM for anti-urease activity, whereas for antiglycation activity, IC_50_ values ranged between 10 ± 0.1 and 23 ± 0.3 µM. The derivatives of single heterocyclic conjugation exhibited the least urease inhibition and antiglycation activity, whereas the double conjugated heterocycles reported better results [131]. H^+^/K^+^ is an enzyme responsible for the secretion of gastric acid, and hence, its inhibition is very crucial. Among the series of synthesised compounds, molecules with electron-withdrawing group, such as bromo, and electron-donating group, such as methoxy substituted in the aryl group (particularly in the para position), resulted in excellent anti-ulcer activity with promising IC_50_ values of 46.0 ± 0.84 µM and 5.4 ± 0.64 µM, which was nearly 15 times higher than that of the standard omeprazole [132]. The in vitro antibacterial and antifungal activities were evaluated using agar-well diffusion and microdilution methods against both Gram-negative and Gram-positive bacteria (*E. coli*, *R. solanacearum*, *X. oryzae* and *K. pneumoniae*) and various fungal strains (*A. niger*, *F. oxysporum* and *A. flavus*). From both antifungal and antibacterial studies, it was confirmed that the molecules possessing thiourea moiety and electron-withdrawing fluoro group were found to have enhanced antimicrobial activity with 28 ± 0.31 mm zone of inhibition, in comparison with the compounds of urea derivatives having electron-donating -OCH_3_ group, showing 13 ± 0.18 mm zone of inhibition [133].

**Figure 46:**
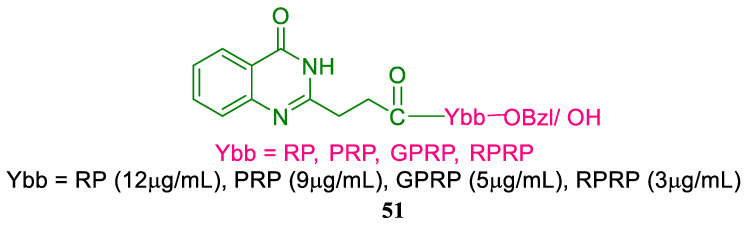
Quinazolinone conjugated to shorter analogues of Bactenecin 7.

Suhas R et al. synthesised a series of quinazolinone-conjugated shorter analogues (**51**) of Bactenecin 7 (Bac7) peptides (RP, PRP, GPRP and RPRP). Bac7, a cathelicidin-derived peptide of bovine neutrophil granules having antimicrobial properties has 59 amino acids with three tandem repeats of a tetra decamers, consisting several PRP triplets and single hydrophobic amino acid (Figure 46). Therefore, the authors have chosen highly active tetrapeptides (GPRP and RPRP) from their earlier work [134], conjugated them to quinazolinone moiety and examined the antimicrobial activity. The results reported that tetrapeptide analogues showed a higher potent activity than their tri- and dipeptide analogues. The authors examined antimicrobial activity of peptides alone, heterocyclic conjugates and the hydrogenolysed conjugates against a panel of pathogens. The results showed that all the conjugates have shown a more superior activity than the starting compounds. The hydrogenolysed tetrapeptide conjugates exhibited almost three to four folds more activity than the standard drugs used. In vitro antibacterial activity was tested against pathogens of both Gram-positive organisms, such as *K. pneumoniae* and *P. putida*, and Gram-negative organisms, such as *S. faecalis*, *S. pyogenes* and *E. coli*, by agar-well diffusion and microdilution method. The antifungal activity was examined against *A. flavus*, *C. capsisi*, *F. oxysporum*, *F. verticellartar* and *A. alternata* by using the above methods. The activities of the compounds were dependent on the charge, hydrophobicity and length of the peptide chain. It was observed that, as the length of the peptide chain increased, activity also increased. The conjugates having RP, PRP, GPRP and RPRP exhibited zone of inhibition of 6 ± 0.36 mm to 31 ± 0.32 mm. The tetrapeptide, RPRP (MIC, IC_50_ = 4 µg/mL) showed a small increase in activity than the GPRP (MIC, IC_50_ = 5 µg/mL) conjugate, which may be due to the basic nature of Arg and also the bulky guanidine group. When the nitro group and benzyl ester at the C-terminus of the conjugates were removed, all the free-acid-containing compounds exerted high potency, nearly 3–4 times in stopping the growth of microbes. Thus, the quinozolinone conjugated with shorter peptides of Bac7, which are of small size, are easy to prepare, are of simple amino acid composition, of high antimicrobial activity and of low toxicity, making them highly active for the design of pharmaceuticals and the systematic treatment of the antibiotic-resistant strains of fungi and bacteria [135].

**Figure 47:**
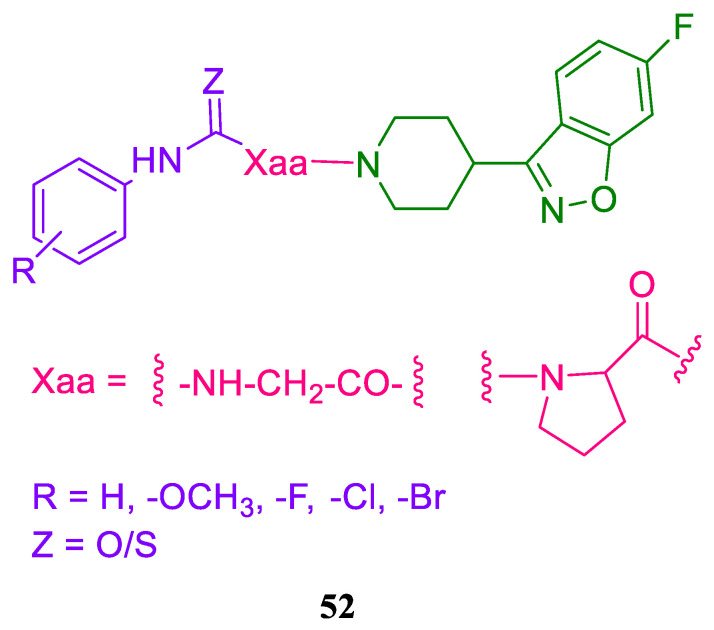
Pro/Gly conjugated with benzisoxazole derivatives.

Shantharam C S et al. synthesised a novel series of thiourea/urea analogues of Pro-/Gly-conjugated benzisoxazole derivatives (**52**). In the series, some of the compounds were highly potent antiglycating agents due to the presence of urea and thiourea analogues (Figure 47). The compounds containing -OCH_3_ and -Br were recognised as active compounds, which hindered the glycation with IC_50_ values of 3.6 ± 0.3 µM and 6.3 ± 0.8 µM, respectively. Comparatively, the compounds containing Pro showed less activity than the Gly-containing moieties, which may be due to the simple nature and small size of Gly, which could have increased the binding properties. Among urea and thiourea derivatives, compounds with thiourea have shown greater activity over urea analogues due to the high nucleophilic power of sulphur. Thus, the synthesised compounds represented a novel class of antiglycating agents. The compounds with EDGs showed good results and, conversely, less activity for the EWGs may be due to the inductive and resonance effects [136].

**Figure 48:**
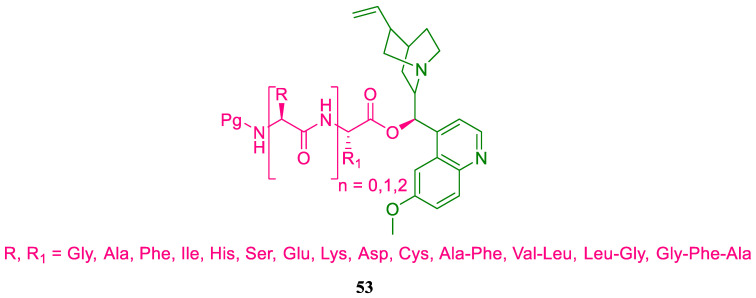
Amino acid conjugated to quinine.

Panda S S et al. have synthesised 14 chirally pure peptide and amino acid conjugates of quinine (**54**) by O-acylation of N-protected acylbenzotriazoles under microwave irradiation (20 W, 50 °C) in the presence of K_2_CO_3_ in anhydrous DMF (Figure 48). The synthesised analogues, along with quinine, were subjected to in vitro antimalarial activity against *P. falciparum*. From the antimalarial bioassay, it was reported that quinine itself exhibited potency with an IC_50_ of 18 nM. Out of 14 synthesised conjugates, 11 exhibited good activity with promising IC_50_ values ranging below 100 nM. Among these, Z-L-Asp(Bz)-QN reported the highest activity in the series with an IC_50_ value of 17nM compared to the quinine counterpart (18 nM) [137].

**Figure 49:**
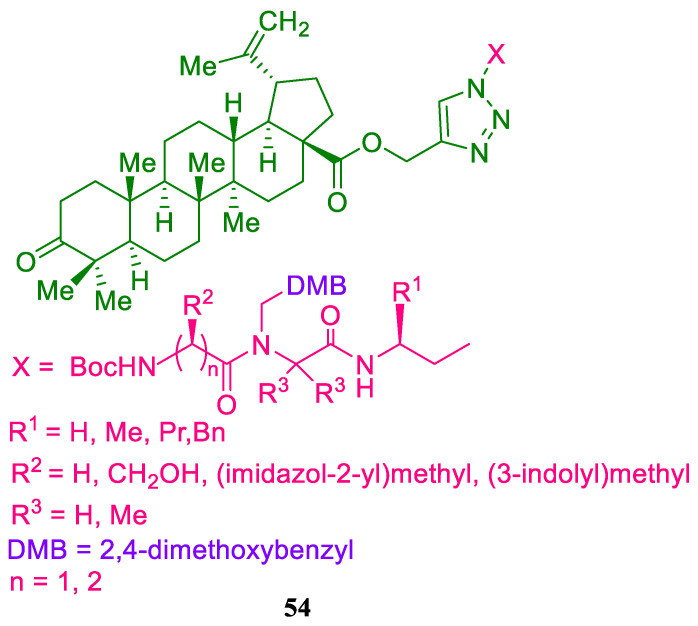
Peptides conjugated to betulonic acid derivatives.

Govdi A I et al. synthesised a series of peptides conjugated to betulonic acid derivatives having 1,2,3-triazoles (**54**) and tested their anti-inflammatory activity (Figure 49). Betulonic acid and its derivatives have a wide range of biological activities with high lipophilicity and lower aqueous solubility. These peptide derivatives of betulonic acid showed high antiviral activity and also acted as a suppressor of the cancer cell growth. The synthesis consisted of two parts. First, the conversion of betulonic acid into its propargyl ester by the reaction of betulonic acid with propargyl bromide in the presence of K_2_CO_3_. On the other hand, azido peptides were prepared by Ugi’s four component reaction of chiral isocyanoazides with 2,4-dimethoxybenzylamine, Boc-protected amino acids and carbonyl compounds (acetone or formaldehyde). The coupling of betunolic acid propargyl ester and azido peptides in 10% copper sulphate–40% sodium ascorbate in a biphasic H_2_O-CH_2_Cl_2_ (1:10) mixture rendered the desired compounds. The synthesised compounds were examined for anti-inflammatory activity using the histamine-induced paw edema model. The compounds having Ser and Ile showed activity. All the tested compounds did not present better activity than the standard, indomethacin [138].

**Figure 50:**
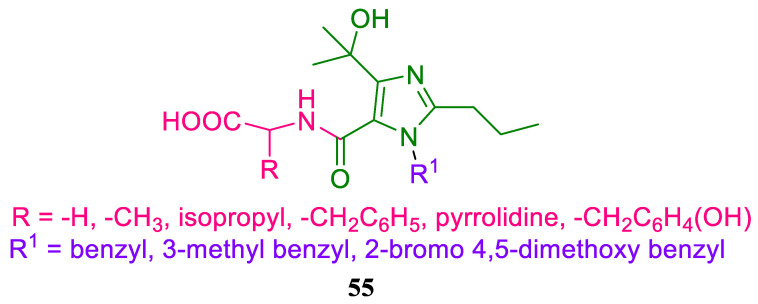
Imidazole-amino acids conjugates.

Ullas BJ et al. synthesised a series of imidazole-amino acid conjugates (**55**) and evaluated their antibacterial, antifungal and antioxidant activities (Figure 50). In the series of compounds synthesised, analogues with Tyr showed excellent oxidation inhibition due to the presence of phenolic functional groups which act as radical scavengers. The majority of compounds with a free -COOH group and more functional groups showed greater activity. Most of the compounds were strain-specific. The effectiveness of the synthesised compounds as antimicrobials was assessed in antibacterial studies against various strains of both Gram-positive bacteria, such as *Ralstonia solanacearurm C. positive staphylococcus* and *X. campestris pv campestris*, and Gram-negative organisms, such *as E. coli* and *X. oryzae*, as well as antifungal studies against *A. niger*, *F. oxysporum* and *F. flavus* by the agar-well diffusion method. The antioxidant activities of the compounds were examined using DPPH method and they showed IC_50_ values ranging from 25 to 40 µg/mL. It was seen from the activity profiles that the majority of the compounds in the study demonstrated better antibacterial activity than antifungal activity. Molecules with Pro/Phe/Tyr were more active than that of analogues containing simple amino acids such as Gly/Ala/Val [139].

**Figure 51:**
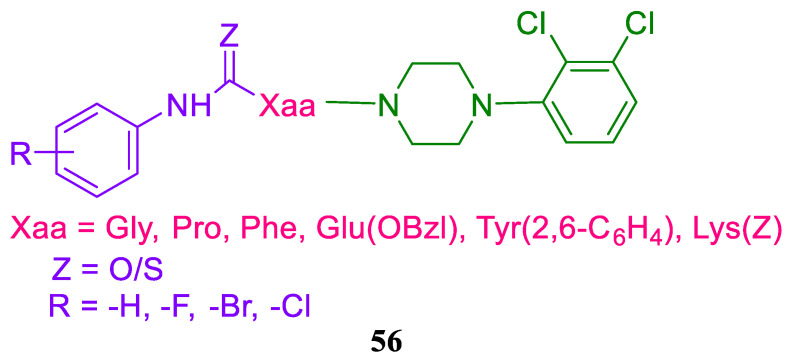
Urea/thiourea derivatives of amino acids conjugated 2,3-dichlorophenyl piperazine.

Suyoga Vardhan D et al. synthesised a series of ureas and thioureas from amino acids conjugated to 2,3-dichlorophenyl piperazine (**56**). The urease inhibitory property of the title compounds was tested against jack bean urease. The authors synthesised six series of urea and thiourea analogues by treating substituted phenyl isocyanates/isothiocyanates with amino acids such as Gly, Pro, Phe, Glu, Tyr and Lys conjugated to 2,3-dichlorophenyl piperazine (Figure 51). The compounds containing -F group particularly at para position showed superior urease inhibition activity with IC_50_ values of 2.6 to 4.2 µM compared to the reference inhibitor, thiourea (21.0 µM). Urease inhibition activity was determined by calculating ammonia production using the indophenol method. In the fourth position of phenyl ring, the substituents improved the anti-urease activity. From the activity profile, it was observed that amino acids that were tested alone and their heterocyclic conjugates exhibited less activity. Further, compounds containing halogens showed higher activity than the methoxy groups [140].

**Figure 52:**
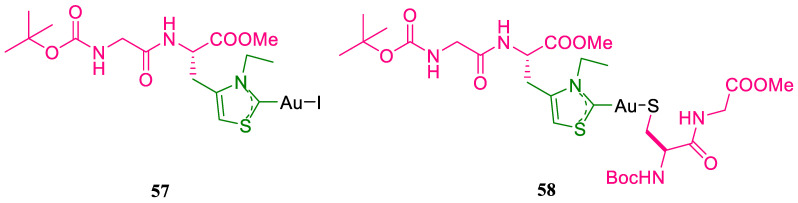
Au^I^ N and S-heterocyclic carbenes (NSHC)-derived peptides.

Gutiérrez A et al. have developed Au^I^ N (**57**), S-heterocyclic carbenes (**58**) (NSHC) derived from peptides containing L-thiazolylalanine (Figure 52). The alkylation of dipeptide having L-thiazolylalanine resulted in the thiazolium salts of bromide and iodide, followed by its conversion to the first NSHC-gold(I) chloride and NSHC-gold(I) iodide having non-proteinogenic amino acid by employing the well-established Ag_2_O path, or by the deprotection which led to the carbene formation as a result of using a strong base, viz. KHMDS (potassium *bis* [trimethylsilyl]amide). The reaction of NSHC-gold(I) iodide with a cysteine-containing dipeptide yielded NSHC-gold(I) thiolate (a bioconjugate). The synthesised analogues were subjected to in vitro cytotoxic activity against three different human cancer cell lines, viz. MiaPaca2 (pancreatic carcinoma), Jurkat (T-cell leukaemia) and A549 (lung carcinoma) using MTT method, which revealed that the two NSHC-gold(I) analogues showed good cytotoxicity with promising IC_50_ values of 16.6 ± 0.2 µM, 6.2 ± 0.1 and 0.4 ± 0.01 µM, respectively. Especially the carbene complex with iodide which emerged as the most potent analogue in all the cases. The NSHC-gold(I) analogue iodide showed the highest cytotoxicity against A549 cell line with sub-micromolar IC_50_ values, which might be due to the enhanced lability of Au-I bond compared to that of Au-S bond or higher lipophilicity [141].

**Figure 53:**
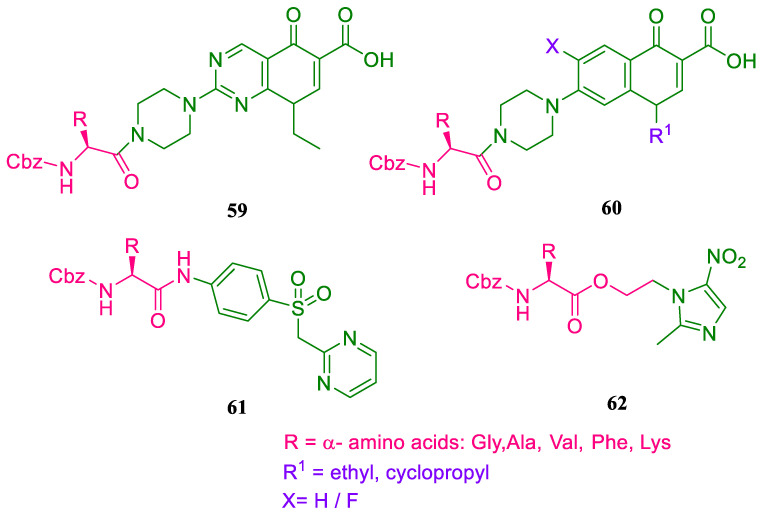
Amino acid conjugated to metronidazole/sulphadiazine/quinolone.

Ibrahim M A et al. synthesised amino acid conjugated metronidazole, sulphadiazine and quinolone antibiotics using benzotriazole method. The authors synthesised amino acid-antibiotics conjugates by coupling pipemidic acid (**59**), norfloxacin (**60**), sulphadiazine (**61**), metronidazole (**62**) and ciprofloxacin with Cbz-N-(aminoacyl)benzotriazoles (Figure 53). Amino acid-antibiotic conjugates were evaluated for their antibacterial activity against *E. coli*, *S. aureus*, *B. subtilis* and *P. aeruginosa*. Among the antibiotic conjugates synthesised, compounds having norfloxacin and ciprofloxacin displayed growth inhibition in all bacterial strains used in the study. Conjugates containing ciprofloxacin exhibited superior activity at lower concentration than any other antibiotic conjugates. Ciprofloxacin conjugates showed maximum inhibition in *B. subtilis* and exhibited variable results in other strains. Norfloxacin conjugates inhibited growth in all the tested strains. It was found that concentration is directly proportional to the activity. Pipemidic acid conjugates inhibited the growth (90%) in only Gram-positive bacteria, such as *S. aureus* and *B. subtilis*. Metronidazole and sulphadiazine conjugates did not show any microbial inhibition in the tested strains. Lipophilicity/hydrophobicity was the key booster for the activity which might have played an important role in the activity. The lipophilicity of the title compounds (log P) and octanol/water partition coefficient Clog P were measured by using Chem Draw Ultra 13.0 with Cambridgesoft software. The log P values of the synthesised conjugates showed maximum values (1.436 to 5.470) compared to the antibiotics alone (−0.5) [142].

**Figure 54:**
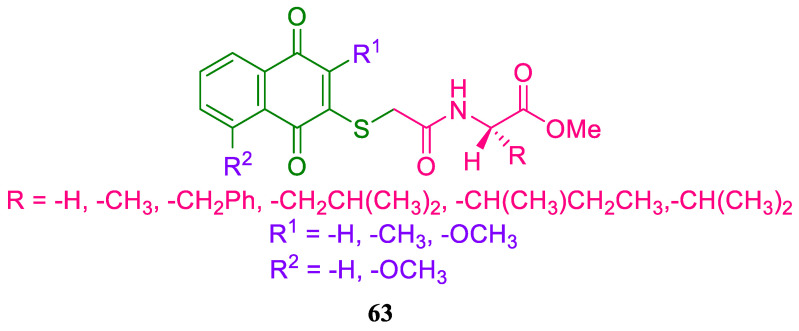
Naphthoquinone amide derivatives.

Sreelatha T et al. synthesised four series of naphthoquinone amide derivatives (**63**) of the bioactive quinones, such as plumbagin, juglone, menadione and lawsone, which were evaluated for their anticancer effect against SAS and HeLa cancer cell lines (Figure 54). The synthesis started by the methylation of quinones in the presence of iodomethane in Ag_2_O followed by Michael addition with thioglycolic acid. Further, these amides of quinone carboxylic acids were treated with methylated amino acids (neutral amino acids such as Gly, Ala, Phe, Ile, Leu, Val, GABA) using DCC/HOBT/DIPEA. The antimicrobial activity of the synthesised compounds was evaluated against two human bacterial pathogens, the Gram-negative *P. aeruginosa* and Gram-positive *Methicillin resistant S. aureus* (MRSA) and a human yeast pathogen and a fungal strain, fluconazole-resistant *C. albicans* (FRCA) using well diffusion method. Among the four series of compounds, menadione and plumbagin conjugates were cytotoxic against SAS and HeLa cell lines with MIC values of 15.6 and 7.8 µg/mL, respectively, whereas lawsone and juglone were inactive. Juglone was inactive to FRCA and MRSA strains. The three-dimensional -QSAR of the synthesised compounds against two cell lines were determined using Vlife QSAR Plus modelling software and it suggested that the presence of EDGs near sulphur increased the activity against HeLa cells. The authors concluded that, by simple functional group transformation, they were successful in synthesising new antimicrobial and cytotoxic quinones [143].

**Figure 55:**
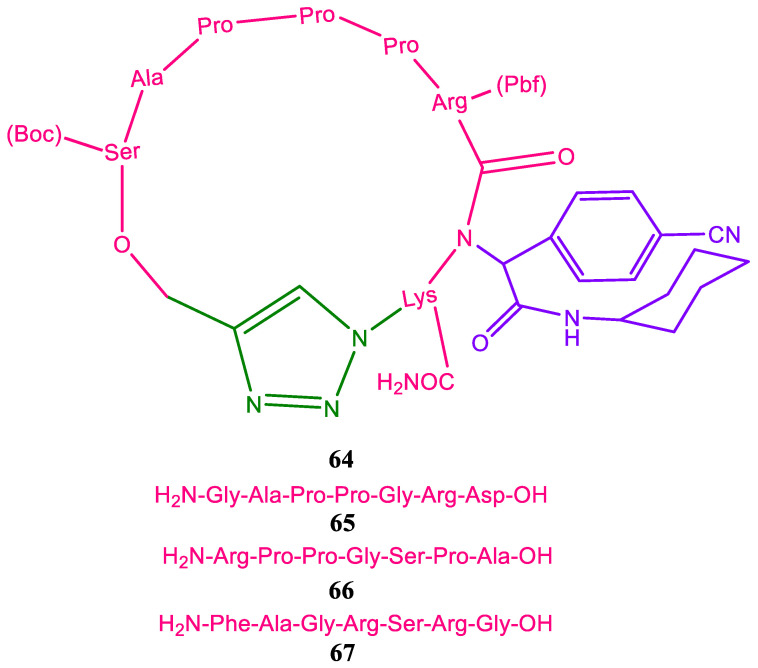
Triazole conjugated cycloheptapeptides.

Tahoori F et al. synthesised a series of cycloheptapeptides (**64**–**67**) incorporating triazole nucleus and examined their anticancer activity (Figure 55). The authors synthesised heptapeptides via SPPS and evaluated their neoplastic activity in ras cancer cells. For improving the anticancer activity of the cyclic peptides, the authors modified the structure into cyclic form by Ugi–Huisgen 1,3-dipolar cyclisation reaction. The compounds containing cyclic heptapeptides showed potent anticancer activity against cancer cells such as C26, PC3 and A549 cells. They used head-to-tail cyclisation in the formation of heptapeptides. The authors used multicomponent reactions (MCRs) through Huisgen [3+2] cycloaddition by using copper as catalyst. The cyclisation was done using 1,3-dipolar cycloaddition of an azide and an alkyne group via Ugi intermolecular reaction. The cyclisation of heptapeptides with a triazole moiety improved the activity by up to 20 times compared to heptapeptides. The compounds containing triazole moiety showed superior anticancer activity, which in turn showed the importance of triazole skeleton [144].

**Figure 56:**
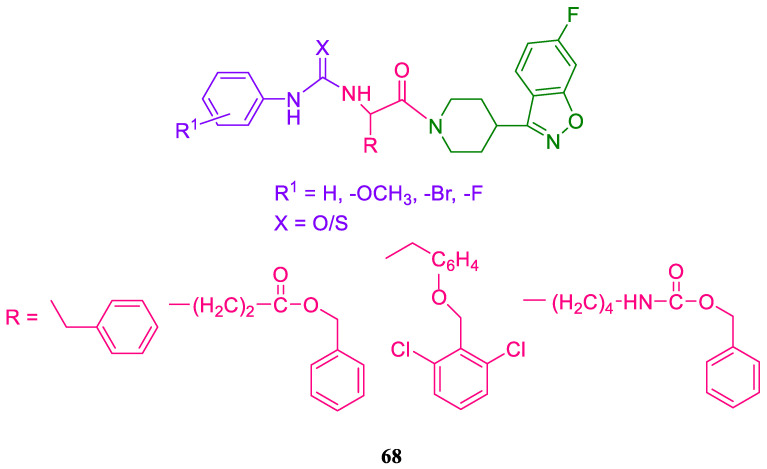
Amino acids-[3-(4-piperidyl)-6-fluoro-1,2-benzisoxazole] conjugates.

Shantharam C et al. synthesised amino acids-[3-(4-piperidyl)-6-fluoro-1,2-benzisoxazole] conjugates (**68**), converted them into thioureas/ureas and examined their antiglycating properties (forty-five analogues) (Figure 56). The compounds containing Tyr with methoxy substituent at the para position showed an IC_50_ value of 1.9µm. Authors have made efforts to improve the antiglycation activity by varying different amino acids (Phe, Tyr, Glu and Lys) and substituted isothiocyanates/isocyanates. The compounds with aromatic ring possessing EWGs, such as -F/-Br (4.8 ± 0.7 µM), and EDG, such as -OCH_3_ (1.9 ± 0.2 µM), exhibited high activities. Further, the compounds with Tyr analogues showed greater activity, whereas Phe-containing derivatives showed moderate activity. Compounds having Glu showed more activity than Lys, indicating that acidic amino acids are more favourable than basic amino acids. Hence, the order of activity was found to be Phe < Lys < Glu < Tyr. The nature of substituents on the phenyl ring of isocyanates and isothiocyanates also played an important role in exerting antiglycation effect [86].

**Figure 57:**
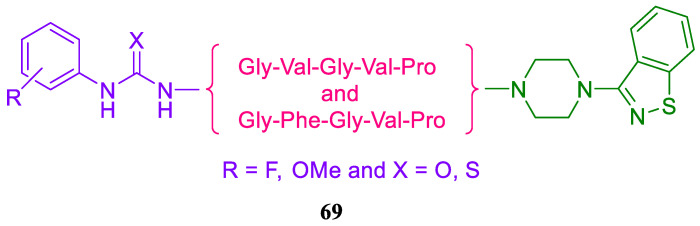
Pentapeptides conjugated to 3-(1-piperazinyl)-1,2-benzisothiazole.

Sharma A et al. synthesised two series of pentapeptides (GVGVP and GFGVP) conjugated to 3-(1-piperazinyl)-1,2-benzisothiazole (**69**) and evaluated their antiglycation, antimicrobial, urease and H^+^/K^+^-ATPase inhibitory activities (Figure 57). The pentapeptides were synthesised using Boc chemistry in solution-phase and conjugated to the heterocycle, followed by converting the N-terminus into ureido and thioureido moieties. The antimicrobial activity was performed using agar-well diffusion and microdilution methods against various bacterial strains, including both Gram-positive (*C. staphylococcus*), Gram-negative (*K. pneumoniae*, *E. coli* and *X. oryzae*) and fungal strains (*F. oxysporum*, *F. monoliforme*, *A. flavus* and *A. niger*). It was found that GFGVP peptide derivatives exhibited increased antimicrobial activity compared to GVGVP counterparts with zone of inhibition values of 33 ± 0.74 mm and 25 ± 0.76 mm, respectively. This could be due to the higher level of hydrophobicity of Phe, thus revealing the importance of hydrophobicity in presenting good activity. In urease inhibition and antiglycation studies, the target molecules having para-substituted methoxy group exhibited superior activity with promising IC_50_ values of 16 ± 0.26 µM against jack bean urease and bovine serum albumin, whereas in the H^+^/K^+^-ATPase inhibition, GVGVP-containing conjugates exhibited an enhanced inhibition of 34.0 ± 0.86 µM IC_50_ value. A general observation was that the title compounds with thiourea group were more active than their urea counterparts [145].

**Figure 58:**
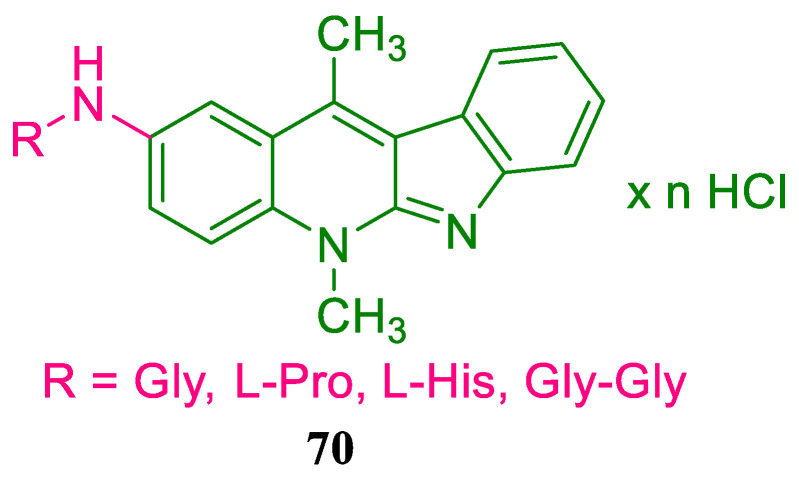
Neocryptolepine-amino acids analogues.

Sidoryk K et al. synthesised a novel series of neocryptolepine analogues (**70**) substituted with a dipeptide or an amino acid at C-9 or C-2 position and developed them as highly potent antiproliferative, antimicrobial and antifungal agents (Figure 58). The reaction of N^α^-*tert*-butyloxycarbonyl-amino acid with 5,11-dimethyl-5H-indolo [2,3-*b*]quinoline-2-ylamine by using a coupling activator, TBTU in the presence of DIEA and HOBt yielded the desired compounds. The peptide derivative of 5,11-dimethyl-5H-indolo [2,3-*b*]quinoline-2-ylamine was also synthesised by the coupling of 5,11-dimethyl-5H-indolo [2,3-*b*]quinoline-2-yl-glycylamide dichloride with Boc-N^α^-Gly using TBTU method in DMF. The in vitro antiproliferative activity was performed against KB cells (cervix carcinoma), which revealed that all the analogues exhibited a higher enhanced activity than the reference standards with promising IC_50_ values ranging between 0.42 and 0.47 µM. However, glycylglycine- and L-His-substituted conjugates showed lesser activity with 1.54 µM and 3.88 µM as IC_50_ values, whereas (DOX) doxorubicin hydrochloride and DiMIQ exhibited 0.84 µM and 1.14 µM as their IC_50_ values. Furthermore, the activity of the Gly conjugate substituted at C-2 position showed enhanced activity than C-9-substituted counterparts. The selected analogues were also examined for the antiproliferative activity against colon cancer LoVo, breast cancer MCF-7, non-small cell lung cancer A549 and normal mice fibroblast BALB/3T3, which revealed that majority of the synthesised conjugates showed enhanced activity, especially the Gly analogue of DiMIQ, which exhibited the highest activity against LoVo and A549 cell lines with IC_50_ values of 0.06 µM and 0.08 µM, whereas L-Pro conjugate of DiMIQ reported the better activity against LoVo cell line with a 0.07 µM IC_50_ value. It was concluded that the amino acid-substituted products at two and nine positions emerged as potential antiproliferative agents than the peptide derivatives. The in vitro antimicrobial and antibiofilm activities of the synthesised analogues were performed using two-fold serial agar dilution method against various bacterial (including both Gram-positive and Gram-negative) and fungal (*C. albicans*) strains, from which it was concluded that certain analogues retained the activity against *C. albicans* biofilms at a lower dosage level than required against free-floating planktonic fungal cells. Finally, the authors concluded that the study provided evidence for the dual action of indoloquinoline conjugates against infectious pathogenic microbes and cancer cell lines, and also as a remedial for biofilm-related infections in humans [146].

**Figure 59:**
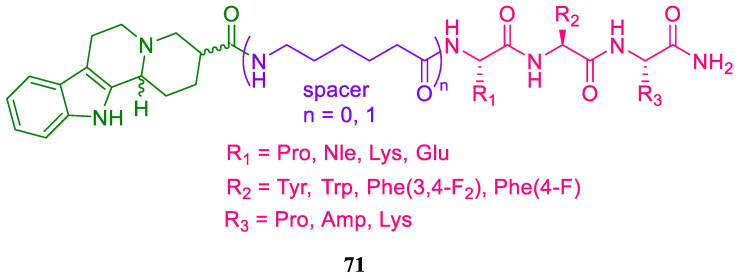
Peptides conjugated to indoloquinolizidine.

Molero A et al. synthesised a combinatorial small library of 80 peptides conjugated to indoloquinolizidine heterocycle (**71**) by solid-state and evaluated them as high affinity at D1 and D2 dopamine receptors in neurodegenerative disorders (Figure 59). The main aim of the current study was to develop several D_1_/D_2_ high-affinity ligands by exploring various peptides around the indoloquinolizidine core to boost the binding affinity at D_2_R. Dopamine receptors are a class of G protein-coupled receptors (GPCRs) that play an important role in the vertebrate central nervous system (CNS). A variety of neurodegenerative diseases may be treated using ligands that act on numerous dopamine receptors. Compounds that can bind to both D_1_R and D_2_R with high affinity might reverse the effects of dopamine depletion and improve motor activation. Studies using indoloquinolizidine-peptides in the past suggested that the interaction at the binding sites might have been caused by the heterocyclic core, whereas tripeptides mostly influenced dopamine receptors’ extracellular regions. By using nine various amino acids (Pro, norleucine (Nle), Lys, Glu, Tyr, Trp, Phe(3,4-F_2_), Phe(4-F), Pro, aminoproline (Amp) and Lys), the authors have synthesised a series of tripeptides and conjugated them to indoloquinolizidine. Among the amino acids, Pro and Amp were neutral and cyclic in nature, and Lys was a straight chain positively charged amino acid. This selection was mainly on the electrostatic nature of the extracellular area (surface) of dopamine receptors. The compounds containing positive charge at the C-terminus showed enhanced ionic interaction with the extracellular region and also increased the binding capacity at dopamine receptors. Among the tripeptides containing Phe(4-F), Phe(3,4-F_2_), Trp and Tyr, there was an increase in activity with K_D_ value of 1.3 ± 0.2 µM and 0.19 ± 0.06 µM for D_1_R and D_2_R, which may be due to increased H-bonding property. The compounds containing positively charged amino acids at the C-terminal residue enhanced the affinity at both dopamine receptors and compounds with trans-configuration at the indoloquinolizidine core, thus prominently enhancing the selectivity towards D_2_R [147].

**Figure 60:**
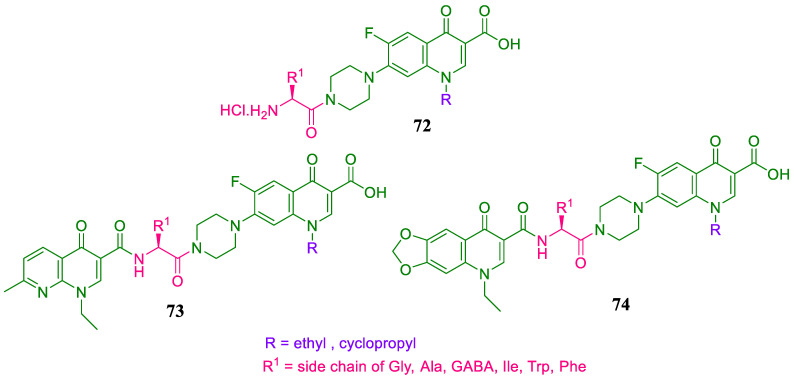
Amino acids conjugated to fluoroquinolone-quinolone.

Panda S S et al. synthesised a series of fluoroquinolone-quinolone conjugated to amino acids (**73**,**74**) using benzotriazole strategy. In this work, authors used four quinolone and fluoroquinolone antibiotics, such as nalidixic acid, oxolinic acid, norfloxacin and ciprofloxacin (Figure 60). Benzotriazole was treated with nalidixic acid and oxolinic acid in the presence of SOCl_2_ to form N-benzotriazolyl nalidixic acid and oxolinic acid, respectively. The norfloxacin and ciprofloxacin were treated with the Boc-protected aminoacylbenzotriazoles in the presence of TEA in dimethyl fluoride to yield amino acid-norfloxacin/ciprofloxacin conjugates, respectively, followed by deprotecting Boc to obtain the desired amino acid-antibiotics conjugates (**72**). The *bis*-conjugates were obtained by coupling amino acid-antibiotics conjugates with N-benzotriazolyl oxolinic acid and nalidixic acid in the presence of TEA in DMF to obtain fluoroquinolone-quinolone antibiotics *bis*-conjugates. The title compounds were evaluated for antibacterial activity against both Gram-negative bacteria, such as *P. aeruginosa* and *S. typhi*, and Gram-positive bacteria, such as *S. aureus* and *S. pyogenes*, using standard agar dilution method. The quinolone conjugates showed mild activity against *S. aureus*, with MIC values around 30 µM and selective activity against *S. typhi*. The *bis*-conjugates of quinoloine-fluoroquinolone having an amidic linkage showed enhanced activity against *S. aureus* and *S. pyogenes*, with 3.3 and 7.8 µM MIC values. In this study, a QSAR was evaluated using CODESSA-Pro (comprehensive descriptors for structural and statistical analysis) software for all the synthesised compounds, including the antibiotics against *S. typhi*, in order to correlate with the results obtained [148].

**Figure 61:**
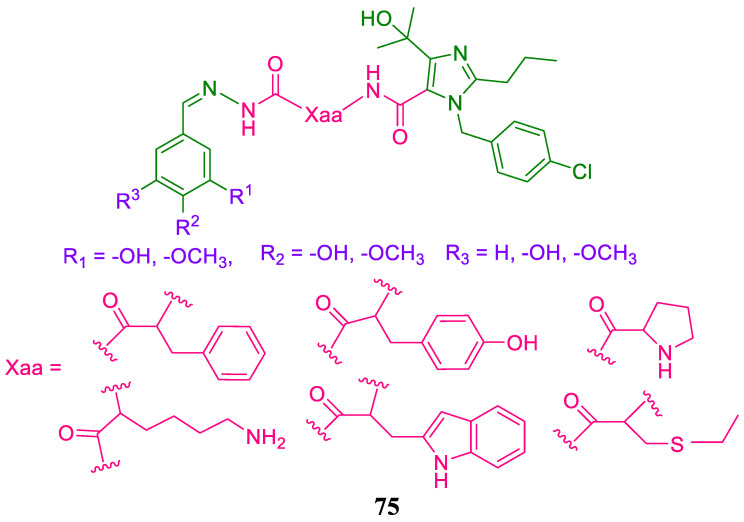
Amino acids–imidazole conjugates.

BJ Ullas et al. synthesised a series of Schiff’s bases from amino acid–imidazole conjugates (**75**) and tested their antioxidant and antimicrobial activities. By using the agar-well diffusion method, antibacterial and antifungal properties were evaluated, as shown in Figure 61. The compounds containing aromatic amino acids showed interesting results. When compared to Lys, a basic amino acid, Met (S-containing)-, and Pro (an imino acid)-containing conjugates showed higher activity with an inhibition zone value of 10 ± 0.45 mm. The compounds containing indole ring from Trp, -OH (phenolic group) from Tyr and phenyl ring from Phe have exhibited greater antioxidant activity (87.90%), which may be due to the electron-donating power and resonating characteristics of phenyl ring. Conjugates having Pro and Met showed the least activity, which may be due to a lack of functional units that are favourable for activity. Chloramphenicol and bavistin were used as standard drugs for antibacterial and antifungal activities, respectively. *X. oryzae* and *E. coli* were used as Gram-positive and Gram-negative bacteria, respectively, whereas the fungal strains used were *A. niger* and *F. oxysporum*. Additionally, derivatives of amino acids-heterocyclic conjugates have shown good results when hydroxyl and methoxy groups were introduced to the phenyl ring [149].

**Figure 62:**
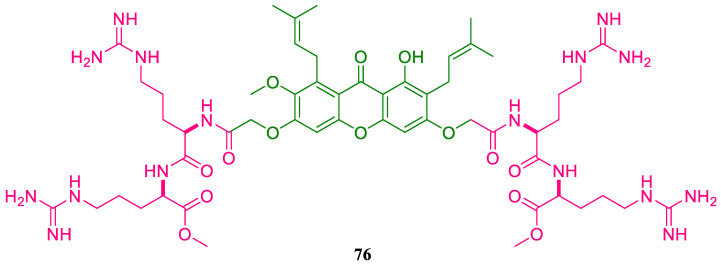
Amino acid-xanthone derivatives.

Koh J et al. have reported the synthesis of novel xanthone derivatives (**76**) by the attachment of amino acids having rigid and hydrophobic cores as promising membrane-active antimicrobials for multidrug-resistant Gram-positive bacterial strains, especially MRSA (Figure 62). A novel series of α-mangostin conjugates were developed by the chemical alteration of basic amino acid moieties and by the functionalisation of two phenolic moieties located at C6 and C3 positions of α-mangostin. The hydroxyl group present at the C1 position was found to be the least reactive due to the formation of a potential intermolecular hydrogen bond between carbonyl group of C9 and hydroxyl group of C1. The alkylation with methyl bromoacetate resulted in corresponding esters which was further hydrolysed with lithium hydroxide to yield carboxylic acids. These were further coupled with His, Arg or Lys using HOBt and DIC at room temperature in anhydrous DMF to yield the key intermediates. Finally, the key intermediate was reacted with H-Arg(Fmoc)-OMe.HCl in the presence of HOBt and DIC, followed by deprotecting the Fmoc using piperidine in DMF, which yielded the highly potent-desired conjugates. On the other hand, a series of isoprenyl-modified conjugates were also synthesised to understand the importance of lipophilic chains. This is not discussed in detail as it is out of the scope of this article. The synthesised conjugates were subjected to time-kill kinetic studies against VRE (vancomycin-resistant enterococci) and MRSA, which revealed that arginine-containing analogues killed the bacteria most rapidly. The multi-passage resistance selection studies demonstrated that arginine analogues prevented the induction of antibiotic resistance. The highly potent Arg analogues were evaluated for the membrane selectivity studies for bacterial or mammalian model membranes. The results revealed that the enhanced cationic charge density of xanthone derivatives was critical for the high membrane selectivity and desired membrane interaction. The antimicrobial properties of the title compounds were performed against *S. aureus* in the presence of bovine serum albumin and the results revealed that Arg conjugates retained the highest activity. The highly potent antimicrobials were further examined for in vivo cytotoxicity using rabbit corneal wound healing model which showed no detrimental effect. Finally, the authors concluded that the highly potent compounds containing hydrophobic xanthone core (IC_50_ = 2 µg/mL), cationic amino acid chains and lipophilic chains exhibited enhanced antimicrobial activity with low toxicity (in vitro and in vivo) and also helped in combating the life-threatening infections [150].

**Figure 63:**
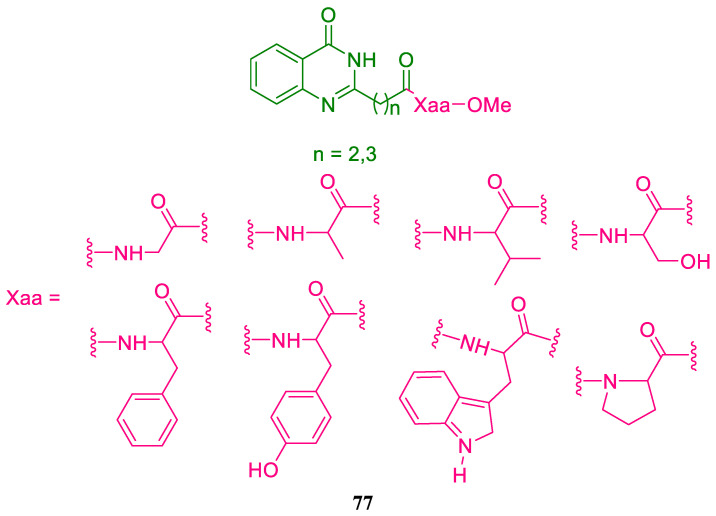
Amino acids linked quinazolinones.

Rakesh K P et al. synthesised two novel series of amino acids linked quinazolinones (**77**) as potential antioxidant, antimicrobial and anti-inflammatory agents by the conjugation of amino acid methyl esters with quinazolinones (Figure 63). The antimicrobial activity was performed against different Gram-negative bacterial strains, including *E. aerogenes*, *S. typhimurium*, *K. pneumoniae* and *E. coli*, and also various fungal strains, such as *F. moniliforme*, *A. niger* and *C. albicans*, by considering streptomycin and bavistin as standard antibacterial and antifungal drugs, respectively. From the structure activity relationship studies and zone of inhibition studies, it was confirmed that conjugates of Phe, Tyr, Trp and Pro presented enhanced anti-inflammatory (38 ± 0.5 mm), antimicrobial (21 ± 0.6 mm) and antioxidant activities (30 ± 0.9 mm) compared to Gly-, Ala-, Val- and Ser-containing conjugates. The authors envisaged that the superior activity of these conjugates could be due to the involvement of an aryl group in the form of phenyl or indole, in the case of Phe, Tyr and Trp, whereas the presence of pyrrolidine nucleus in Pro might have been involved in increasing the activity. Further, the authors have also observed that increasing the length of alkyl chain in the heterocycle decreases the activity, thus suggesting that propyl chain would be preferred for better activity [151].

**Figure 64:**
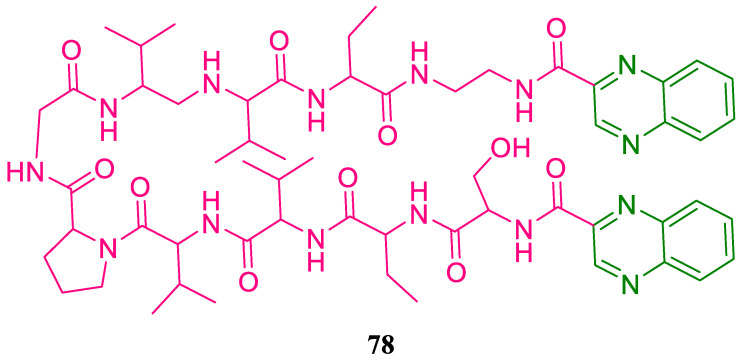
Quinoxaline conjugated to peptides.

Zamudio-Vázquez R et al. designed and synthesised a new library of quinoxaline-conjugated peptides (**78**). The peptidic derivatives were synthesised by classical SPPS and then incorporated with 2-quinoxalinecarboxylic acid (Qxn) in-solution, followed by final deprotection (Figure 64). The synthesised compounds were tested for in vitro anticancer activity in the following four human cancer cell lines: colon adenocarcinoma (HT-29), lung carcinoma (A-549), breast adenocarcinoma (SK-BR-3) and cervical adenocarcinoma (HeLa), by using MTT assay. Among the synthesised compounds, one conjugate having peptidic scaffold with four Val, no N-methylations and L-Ser attached to one of the quinoxalines exhibited the most potent activity with IC_50_ values of 6.8 µM, 11.9 µM, 5.4 µM and 2.7 µM in the above-mentioned cancer cells, respectively. Molecular dynamic simulations were studied by using the GPU-based PMEMD module of the Amber12 package. It was found that the above compound that inhibited autophagy encouraged the development of acidic compartments, where it accumulated and obstructed the enhancement of autophagy. Further, this compound interfered with the mitochondrial membrane potential and showed an increase in mitochondrial ROS, which led to cell death. Thus, the aforesaid compound may be a potent therapeutic candidate for the treatment of cancer because of its cytotoxic property and protease resistance [152].

**Figure 65:**
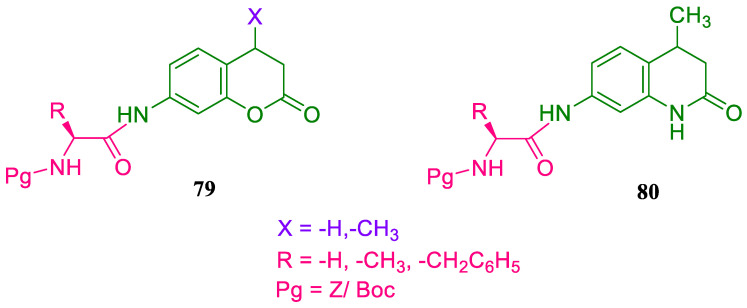
Coumarin/quinolinone conjugated to N-protected amino acids.

Küçükbay F Z et al. synthesised a series of heterocycles, such as coumarin (**79**)/quinolinone (**80**) conjugated to N-protected amino acids (Gly/Ala/Phe), and evaluated their carbonic anhydrase inhibitory activity (Figure 65). Carbonic anhydrase (CAs) catalyses the bidirectional conversion of CO_2_ and H_2_O into bicarbonates and protons. The title compounds were synthesised by reacting N-protected aminoacylbenzotriazole with appropriate coumarin analogues under microwave heating and, on the other hand, N-protected aminoacylbenzotriazole were reacted with 7-amino-1,2,3,4-tetrahydro-2-quinolinone in good yields. The compounds were tested for carbonic anhydrase inhibitory activity against human isoforms, such as hCA I, hCA II, hCA IV and hCA XII. The coumarin conjugates were not effective hCA I and II inhibitors, whereas quinolinone conjugates were inactive as enzyme suppressors in submicromolar range, with an inhibition constant ranging from 0.11 to 0.79 µM [153].

**Figure 66:**
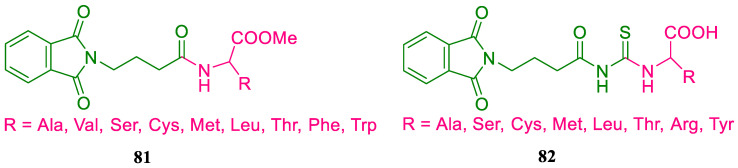
*N*-α-amino acids conjugated to phthalimide moieties.

Al-Masoudi N A et al. synthesised a series of *N*-α-amino acid derivatives conjugated to phthalimide moieties with the aim to develop new non-nucleoside reverse transcriptase inhibitors (NNTRIs) (Figure 66). From the previous literature, the authors found that the carbon-spacer length is crucial in the synthesis of potent anti-HIV agents. Hence, in this work, they selected phthalimido-(substituted-alkyl-2-thioureido) alkyl carboxylic acid containing three carbon atoms spacer. The conjugates, methyl 2-(4-(phthalimido-2-yl)butanamido) alkyl carboxylates (**81**), were synthesised by the acylation of phthalimide acid followed by treatment with ammonium thiocyanate to obtain isocyanate intermediates, which were coupled with various amino acids (Ala, Ser, Cys, Met, Leu, Thr, His, Arg and Tyr). Conjugates containing phthalimido-(substituted-alkyl-2-thioureido) alkyl carboxylic acid derivatives (**82**) were synthesised starting from NH_2_-AA-OMe (AA = Ala, Val, Ser, Cys, Met, Leu, Thr, Phe, Trp and Pro). The newly synthesised conjugates were tested for antiviral activity against HIV-1 and HIV-2 strains in MT-4 cells. The compounds containing heterocyclic structures, such as Trp and methyl proline moieties, exhibited inhibition of HIV-replication at EC_50_ lower than the CC_50_ compared to the standard nevirapine and azidothymidine. The most active conjugates showed an effective 50% decrease in the cytopathogenicity induced by HIV-1 at CC_50_ of 29.61 and 101.90 μM and EC_50_ > 4.23 and 20.38 μM, resulting in a selective index of 5 and 7, respectively, at non-toxic concentration. Conjugates were subjected to docking study using HIV-1 reverse transcriptase enzyme (Autodock Tools) to study the interactions and binding energy. One of the active compounds, methyl 2-(4-(phthalimido-2-yl)butanimido)-3-(indol-3-yl)propanoate, showed three hydrogen bond interactions, namely Lys22 with O-OMe, Thr377 with NH of indole and Lys395 with O atom of phthalamide. Conjugates showed moderate activity against HCV genotype 1b in the Huh-5-2 replicon system. The anti-HIV activity indicated that the synthesised conjugates act as new candidates for non-nucleoside reverse transcriptase inhibitors [154].

**Figure 67:**
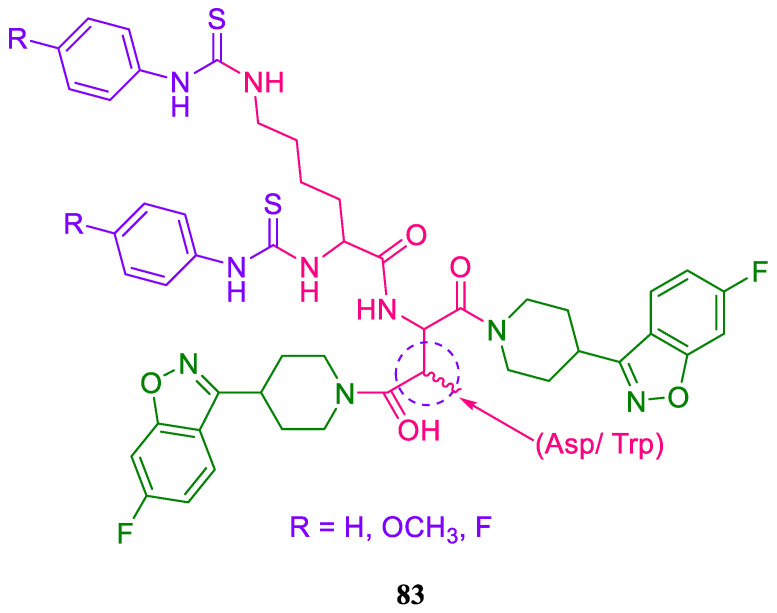
Peptides conjugated to 6-fluoro-3-(piperidin-4-yl) benzo[*d*]isoxazole.

Kumara H K et al. synthesised a novel series of *bis*-thiourea derivatives of four dipeptides consisting of Lys-Trp, Lys-Asp, lys(D)-Trp, lys-Asp conjugated to 6-fluoro-3-(piperidin-4-yl) benzo[*d*]isoxazole (**83**). The title conjugates were evaluated for their in vitro antioxidant activity using N, N-dimethyl-p-phenylenediamine dihydrochloride (DMPD), DPPH and 2,2-azinobis-(3-ethylbenzothiazoline-6-sufonic acid) (ABTS) assays (Figure 67). All the synthesised molecules exhibited good antioxidant activity at lower concentration. The presence of covalently conjugated amide bond, EDGs (methoxy), different substituents on the phenyl ring of thiourea moiety, nature of the amino acids in the dipeptides and conversion of α and ε-amino functions into *bis*-thioureas led to a marked increase in the antioxidant activity of the final compounds. It was observed that D-configuration of Lys in the dipeptidic part exhibited more potent activity than the dipeptide derivatives with L-configured Lys. Further, the conjugates with Trp exhibited better activity (IC_50_ values: 20 ± 1.64, 25 ± 0.95 and 20 ± 0.98 µg/mL determined by DPPH, DMPD and ABTS assays, respectively) than the Asp conjugates that may be due to high oxygen radical absorbance capacity, i.e., the presence of aromatic nature and indole ring in the case of Trp. The in vitro toxicity study of the synthesised compounds showed that they are less toxic to Hela and A459 cell lines. Thus, the structures could be considered as a reference in the development of a new series of antioxidant agents [155].

**Figure 68:**
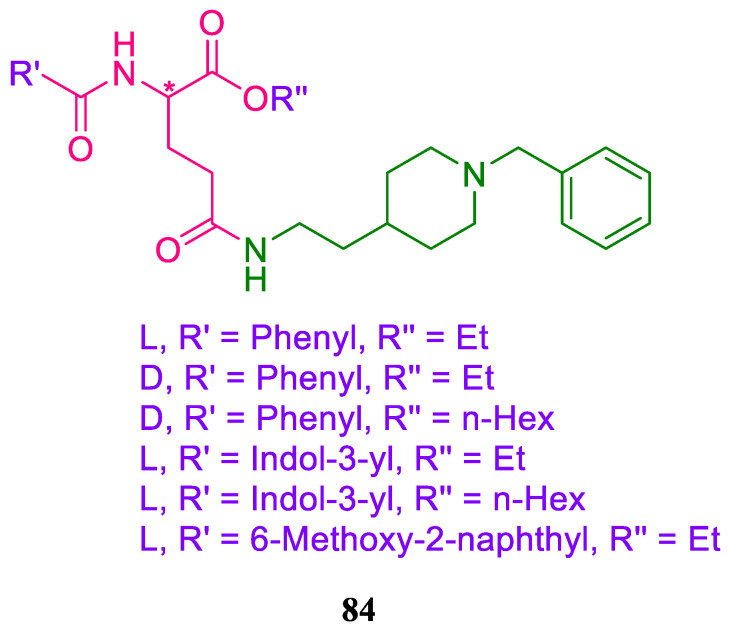
*D*- and *L*-Glu derivatives having N-benzylpiperidine.

Monjas L et al. have applied solid-phase and chemoenzymatic synthetic routes for the development of multifunctional *D*- and *L*-Glu derivatives (**84**) as potential agents for the treatment of many neurodegenerative diseases such as Alzheimer’s disease and cerebral ischemia (Figure 68). The steps involved in solid-phase synthesis are: N-Fmoc-Glu(OAll)-OH (*L* and *D* configuration) was attached to Wang resin, Alloc was deprotected using Pd(PPh_3_)_4_/PhSiH_3_, N-benzyl piperidine (NBP) was then attached using EDC/HOBt, Fmoc was removed using piperidine and various amino acids were attached, resin was then removed using TFA and finally, esterification was performed with various alcohols. The authors have observed that the preparation of *D*- and *L*-Glu derivatives having N-benzylpiperidine at γ-position was possible only by implementing solid-phase synthesis. In chemoenzymatic synthesis, the authors decided that Cbz would be the proper substrate. This method involved the reaction between N-Cbz-*D*-Glu(OEt)-OEt and NBP-NH_2_ in the presence of CAL-B (Lipase B from Candida antaretica) to yield mono-amidated product. The target molecules exhibited enhanced multifunctional properties, such as protection of neuro-related properties against oxidative stress caused by the deprivation of oxygen and glucose, or by the mixture of rotenone with oligomycin and inhibiting human ChEs in sub-micromolar and micromolar range. The synthesised molecules were examined for the inhibition of human plasmatic BuChE (hBuChE) and human recombinant AChE (hAChE) using donepezil as reference standard using Ellman method. The results revealed that the diethyl derivatives failed as enzyme inhibitors, whereas novel donepezil-based *D*- and *L*-Glu derivatives, especially the molecule having NBP moiety in the α-carboxyl group, inhibited hBuChE 10 times compared to hAChE with IC_50_ values of 3.00 ± 0.09 and 0.17 ± 0.01 µM against hAChE and hBuChE cell lines, respectively. The *D*-Glu derivatives exhibited a better enhanced activity than *L*-Glu derivatives. Finally, the hexyl and ethyl ester derivatives emerged as better inhibitors with promising IC_50_ values in both human hChEs [156].

**Figure 69:**
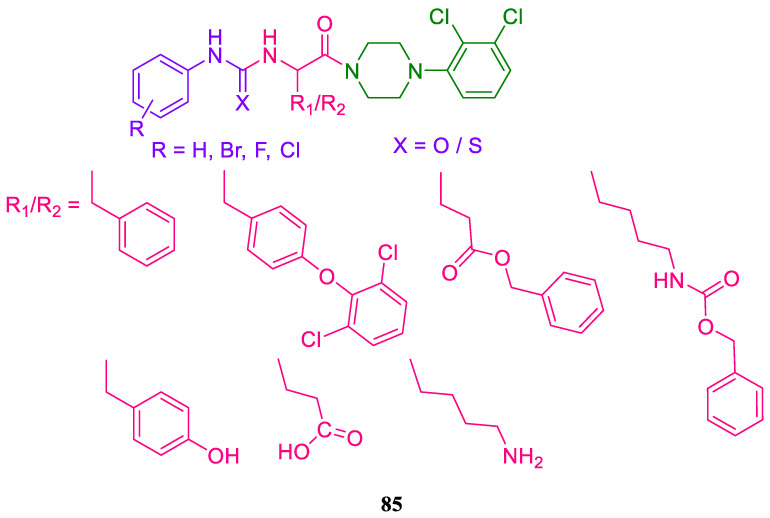
Amino acids conjugated to 2,3-dichlorophenyl piperazine.

Suyoga Vardhan D M et al. synthesised a library of eighty-four ureido/thioureido analogues of amino acids conjugated to 2,3-dichlorophenyl piperazine (**85**) and evaluated them as antiglycating agents (Figure 69). The compounds containing thiourea group exhibited more activity than urea-containing derivatives. The synthesised compounds exhibited up to 20 times greater activity than the standard, rutin. The compounds containing EWGs (IC_50_ = 2.6 ± 0.9 µM) emerged as the active analogues compared to EDGs, in that, the compounds bearing halogen emerged as highly potent antiglycating agents. The amino acids used in this study were Phe, Tyr, Glu and Lys. Among the amino acids, Phe (4.2 ± 0.7 µM)- and Lys (3.2 ± 0.9 µM)-containing compounds exhibited lower antiglycation activity compared to compounds containing Glu (4.2 ± 0.9 µM) and Tyr (2.9 ± 0.6 µM). This suggested that the presence of acidic rather than basic amino acids and phenyl groups containing amino acids favours antiglycation activity. In this work, the authors reported that there was no difference in activity when O of urea was replaced by S [157].

**Figure 70:**
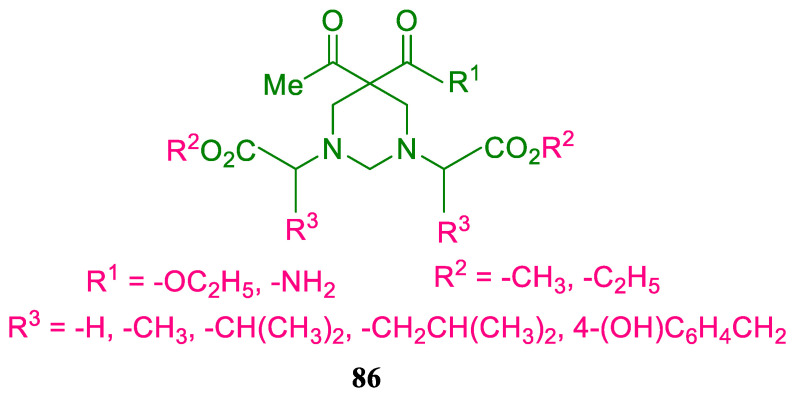
N-substituted α-amino acids analogues consisting hexahydropyrimidine moiety.

Latypova D R et al. synthesised a novel series of N-substituted α-amino acids analogues consisting hexahydropyrimidine moiety (**86**) and tested their cytotoxicity (Figure 70). The authors examined the cytotoxicity of the compounds in cancer cells. One-pot synthesis of Mannich reaction of 3-oxobutanamide or ethyl acetoacetate with HCHO and amino acid hydrochlorides in acetate buffer rendered diastereomerically pure N-substituted hexahydropyrimidine-amino acids in 71 to 89% yield. The target compound was generated as four stereoisomers based on the information from NMR spectra and chiral HPLC evaluation. These included three diastereomers, two of which were in the meso form. At the same time, one pure (*S*, *S*)-enantiomer of the relevant compound was obtained from (*S*)-Ala under the identical conditions. The cytotoxic activity was examined in human cells (HEK293) and cancer cells (SH-SY5Y and Jurkat). It was observed that analogues of hexahydropyrimidine showed moderate inhibitory activity with IC_50_ ranging from 37 to 88 µM against all the tested cell lines. From the activity profile, it was noticed that compounds with EDGs, such as -CH_3_, showed higher cytotoxicity than the aromatic and EWGs. It is important to notice that among the diastereomers, (-)-isomer exhibited weak toxicity against Jurkat SH-SY5Y, and more prominently against HEK 293 cells [158].

**Figure 71:**
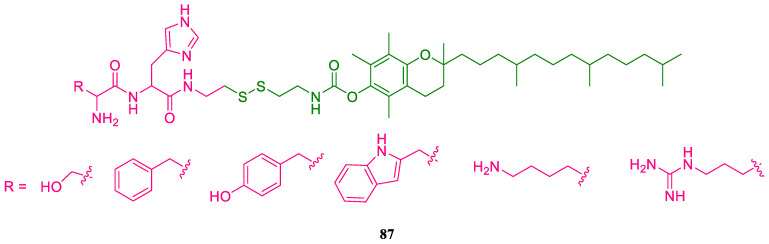
Tocopherol-based cationic lipids conjugated to peptides.

Liu Q et al. designed and synthesised a series of tocopherol-based cationic lipid (**87**) containing imidazole moiety (pH sensitive) in the dipeptide group and disulphide linkage (reduction-responsive) (Figure 71). The molecules had tocopherol at one end and different amino acids at the other end in order to investigate their structure–activity relationship. In the synthesis, an imidazole group with the pH buffering capacity was attached into cationic head group using His. Acid-base titration of the compounds revealed that these have effective buffering ability. The improved transfection activity is achieved by the target lipid ability to load and deliver pDNA into cells with good efficiency. The liposomes formed from synthesised compounds and co-lipid DOPC (1,2-dioleoyl-sn-glycero-3-phosphocholine) could effectively bind and condense DNA into nanoparticles. According to the findings, cationic dipeptide headgroups had a substantial impact on the physico-chemical and biological characters of cationic lipids. The findings not only point to such lipids as potential non-viral gene vectors with pH and reduction-dual sensitive properties, but also assist in creating new non-viral gene vectors with greater effectiveness and decreased cytotoxicity [159].

**Figure 72:**
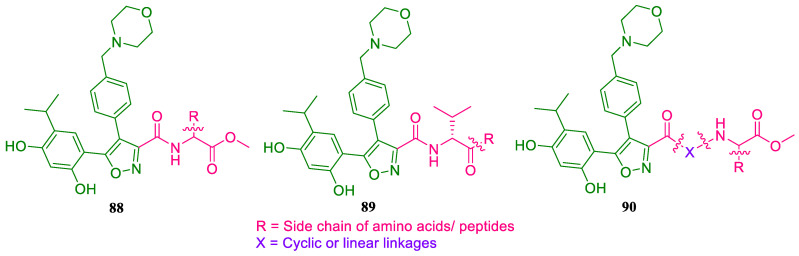
4,5-diarylisoxazoles conjugated to amino acids.

Zhang C et al. have developed a novel sequence of 4,5-diarylisoxazoles by diversifying the conjugation of various amino acid residues (**88**,**90**) and evaluated them as potential Hsp90 inhibitors (Figure 72). The synthesis involved the condensation of various amino acids with a known (modified) Hsp90 inhibitor in the presence of HOAt, HATU and DIPEA that yielded amides that were further subjected to the deprotection of benzyl groups, using BCl_3_ at 0 °C to obtain final compounds. In another series, methyl amino acid esters were reacted with pyrimidine-5-carboxylic acid, yielding pyrimidine amides which, on further treatment with TFA, furnished piperazine derivatives, which then underwent condensation with isoxazole-3-carboxylic acid in the presence of HOAt, HATU and DIPEA, and later the deprotection of O-benzyl was conducted, which led to the development of target molecules. In the third series, the amino acid methyl esters were treated with the abovementioned Hsp90 inhibitor (an acid) having various linkers, which formed the intermediates, and which were later N-deprotected using TFA and subjected for condensation reaction with isoxazole-3-carboxylic acid, and finally, they yielded the desired products by O-debenzylation using BCl_3_. The synthesised molecules were pharmacologically evaluated for anticancer studies on cancer cell lines such as NCI-H3122 using SRB assay and fluorescence polarisation (FP) assay. The structure–activity relationship studies revealed that the isoxazole-3-amido analogues conjugated to unnatural and natural amino acid methyl esters reported good potency against Hsp90α with promising IC_50_ values ranging between 0.07 and 0.84 µM, whereas compounds containing *L*-amino acid analogues in the same subseries were either the least or inactive in H3122 cell line. Furthermore, the authors optimised the former compounds by the conjugation of Val-derived esters and amides in which ethyl esters exhibited enhanced antiproliferative effect by ten folds and less potency by two folds of the methyl ester counterparts. Both the optimised esters were more highly potent by ten folds than the reference standards. The conjugation of the second set of amino acids, viz. Leu, Val and Phe, to the former compounds (Val moiety) furnished dipeptide analogues. The authors reported the moderate potency for Hsp90 (0.2 to 0.4 µM), whereas Val–Phe dipeptide analogue exhibited enhanced cellular potency with a more satisfactory IC_50_ value (79 nM) than its counterparts. The authors further examined for tripeptides and tetrapeptides and reported that tripeptides were 20 folds higher in potency than the tetrapeptides with promising IC_50_ values ranging between 0.03 µM and 0.63 µM, respectively. The molecular docking studies was performed on Hsp90 N-terminal domain having ATP-binding site, which revealed that the molecule having ethyleneglycol linker and Val moiety at the terminal end formed polar and additional apolar interactions with various amino acids. Therefore, the authors suggested that the synthesised molecules reported enhanced binding potency against Hsp90 and inhibited the growth of various cancer cell lines such as BT-474 (breast cancer cell) and H3122 (lung cancer cell) with promising IC_50_ values [160].

**Figure 73:**
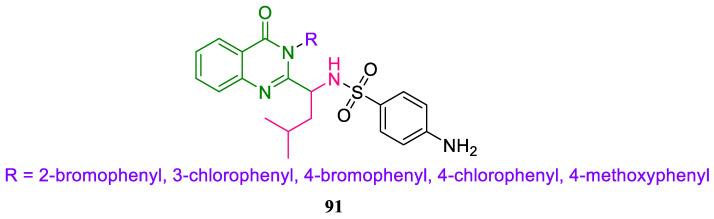
Leu linked sulphonamide-quinazolinone hybrid derivatives.

Patel T S et al. synthesised a new series of Leu-linked sulphonamide-quinazolinone hybrid derivatives (**91**) as a new class of highly potent antimalarial agents (Figure 73). The synthesis involved the following steps: N-heterocyclisation of various aromatic amines with previously synthesised N-acylanthranilic acid under microwave irradiation (350 W) and green solvent such as triethylamineacetate along with a catalyst. Finally, the acid hydrolysis of the former compounds by microwave-assisted and conventional heating conditions furnished the entitled de-acetylated analogues. The target molecules were evaluated for in vitro antimalarial activity, antiplasmodial assay (p-LDH and MTT), enzyme inhibition assay and cytotoxicity against Vero cells. The synthesised compounds were examined for in vitro antimalarial studies on *P. falciparum* strain using pyrimethamine as reference standard. All the target molecules reported enhanced potency with IC_50_ < 0.20 µg/mL compared to the standard drug. This may be due to the presence of sulphonamide-linked amino acid at 2-position in the quinazolinone residue. This may especially be because the molecules having phenyl ring substituted by 4-OMe, 3-Cl, 4-Cl, 2-Br and 4-Br reported to have exhibited enhanced activity compared to other counterparts. The anti-plasmodial assay was performed in order to determine the selectivity (p-LDH) and toxicity (MTT) of the synthesised compounds. The p-LDH assay reported that all the synthesised molecules exhibited moderate to the least potency compared to the standard chloroquine, except the molecules having 3-Cl- and 4-Cl-substituted phenyl ring, which were reported to be highly potent. In MTT assay, selectivity indices (SI) of *P. falciparum* were calculated by using CC_50_ and EC_50_, which showed that all the synthesised compounds exhibited SI values of >1 and thus confirmed that the synthesised molecules were specific only against *P. falciparum* but not RBCs. Further, the compounds were subjected for cytotoxic assay against Vero cells and inhibition action of bovine liver dihydrofolate reductase (DHFR) enzyme. The cytotoxic assay revealed that concentration of the molecules was exceeding 15 µg/mL against Vero cells, thus exhibiting non-toxic behaviour. Furthermore, the authors suggested that the compounds having phenyl ring substituted by 3-Cl, 4-Cl and 4-OMe emerged as potential DHFR inhibitors. The synthesised molecules were subjected to molecular docking studies on the active site of Pf-DHFR enzyme to study the various interactions among the molecules, such as H-bond and π-π interactions. The docking scores revealed that the compounds having phenyl ring substituted by 2-Br, 3-Cl, 4-Br, 4-Cl and 4-OMe reported to exhibit the docking scores ranging between −3.7 and −4.21, and between −34.56 and −43.08 binding energies with a more enhanced activity than the pyrimethamine and trimethoprime standards. Later, the target molecules were docked against human DHFR (1MVT) to study the selectivity of quinazolinone-sulphonamide analogues, which reported that the highly potent molecules interacted poorly with h-DHFR when compared to the Pf-DHFR. The in silico pharmacokinetic properties of the synthesised conjugates were found to obey all the four Lipinski’s rule of five by violating only one rule, thus proving it to be a drug-likeness candidate with good oral bioavailability [161].

**Figure 74:**
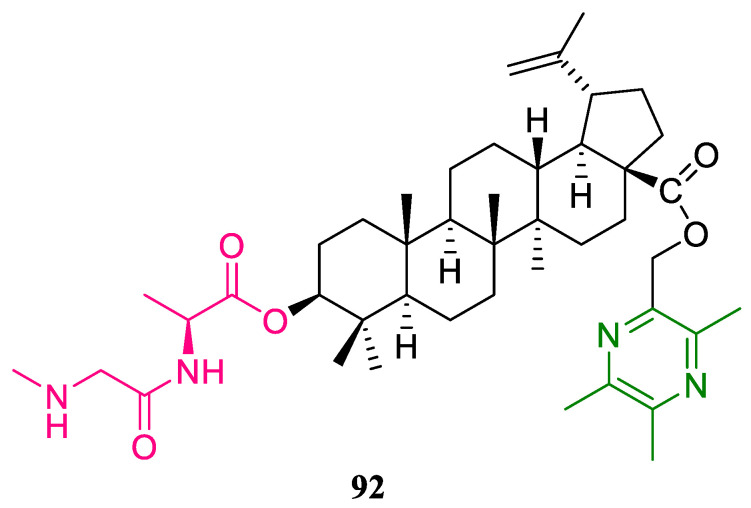
Peptide conjugates of pyrazine derivatives.

Xu B et al. reported the synthesis of 3β-hydroxy-lup-20(29)-ene-28-oic acid-3, 5, 6-trimethylpyrazin-2-methyl ester (**92**) by the conjugation of acid/dipeptide with ligustrazine-betulinic acid as a novel highly potent anticancer agent (Figure 74). The lead molecule was screened for cytotoxic activity against five different cancer cell lines (A549, BCG-823, Hela, HT-29, HepG2) by standard MTT assay and it was revealed that the molecules with smaller aliphatic amino acids reported higher activity than the molecules having higher molecular weight aliphatic amino acids. The most active compound exhibited IC_50_ values of 3.09 ± 1.49, 1.70 ± 0.34, 1.74 ± 0.99, 1.79 ± 0.28, 3.25 ± 1.10 and 10.84 ± 0.27 µM against HepG2, HT-29, Hela, BGC-823, A549 and MDCK cell lines, respectively. The authors have also observed that amino acid derivatives were the least active when compared with dipeptide derivatives [162].

**Figure 75:**
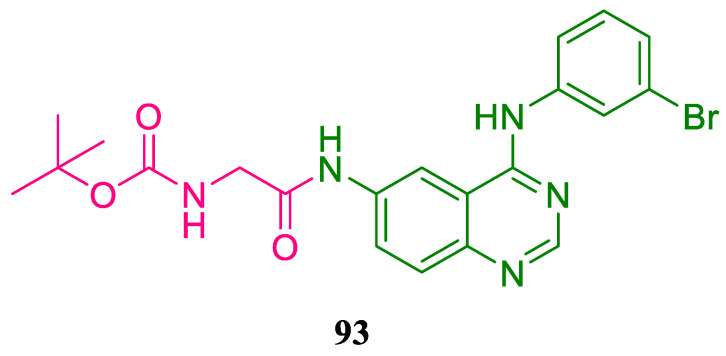
4-anilinoquinazoline-amino acid derivatives.

Zheng Y G et al. have synthesised a series of 4-anilinoquinazoline-amino acid derivatives (**93**) as a new class of anticancer agents (Figure 75). The reaction of 2-amino-5-nitrobenzoic acid with formamidine acetate in the presence of 2-methoxyethanol resulted in 6-nitro-3H-quinazolin-4-one, followed by the chlorination which yielded the intermediate. The intermediate was then refluxed with different substituted aniline in the presence of isopropanol, followed by the reduction of nitro moiety using Fe powder, which yielded the amine intermediates. Finally, the reaction of amine intermediates with diverse N-Boc protected amino acids yielded the desired compounds. The title compounds were subjected to anticancer activities against human hepatocellular carcinoma cell HepG2 using SRB assay. Among the synthesised analogues were N-Boc protected Gly moiety at the sixth position of quinazoline and N-Boc protected Ala—the former exhibited enhanced inhibitory activity of EGFR (IC_50_ = 0.0032 µM) with high selectivity of nearly 2000-folds of other kinases, such as MEK, VEGF2, RAF, c-MET and Aurora B. The authors observed that up-regulating the Bax expression and down-regulating Bcl-2 expression and a decrease in the potential of mitochondrial membrane led to the promotion of mitochondrial cytochrome c, which was further released into the cytoplasm and activated caspase-3, and finally induced HepG2 cell apoptosis. The molecular docking studies were performed on the active site of EGFR and the results revealed that the target molecule could successfully bind with EGFR [163].

**Figure 76:**
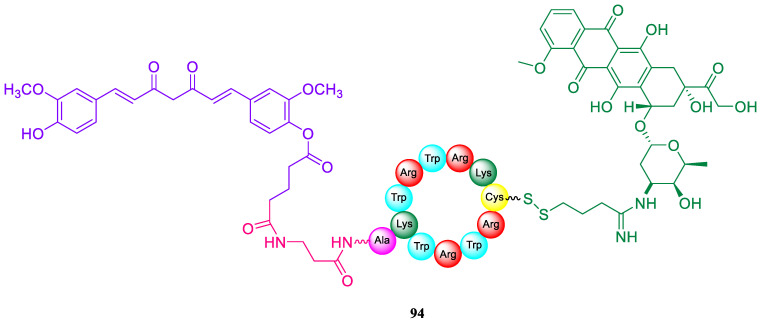
Doxorubicin-curcumin conjugated to cyclic peptide analogues.

Darwish S et al. have reported the synthesis of doxorubicin-curcumin conjugated cyclic peptide analogues (**94**) for the improvement of curcumin solubility and also to develop a conjugate having anticancer property (Figure 76). The amphiphilic trifunctional cyclic peptides, viz. (C(WR)_4_K_2_(β-A)), was synthesised by the implementation of SPPS employing Fmoc/*t*Bu strategy. Appropriately, side-chain-protected amino acids, namely β-alanine, Lys, Cys, Arg and Trp, were assembled on Trp(Boc)-2-chlorotrityl resin to produce side-chain-protected linear peptide. The Fmoc moiety at N-terminal was deprotected using piperidine in DMF, followed by the breaking of protected peptide by the resin in mild acidic solution, which yielded a linear peptide having free carboxylic group. The linear-protected peptide was cyclised under diluted and anhydrous conditions by using DIC/HOAt as coupling agent, followed by the deprotection of the side chain, resulting in an amphiphilic cyclic peptide. Doxorubicin and curcumin were subjected to suitable modification so that they could be conjugated to the cyclic peptide effectively, thereby ensuring that their activity is not lost. The title compound, DPCC (dox-peptide-curcumin conjugate) was produced by the amide coupling reaction between monocarboxylic group of curcumin and amine moiety of doxorubicin-cyclic peptide by using PyBOP (phosphonium hexafluorophosphate) and HOBt in a basic medium for three hours. Doxorubicin-cyclic peptide was treated with curcumin monoglutarate to furnish doxorubicin-cyclic peptide-curcumin conjugate. The synthesised conjugates were biologically evaluated for the cytotoxic assay on two different human cancer cell lines, such as leukaemia carcinoma cells (CCRF-CEM), ovarian carcinoma cells (SKOV-3) and normal kidney cell line (LLCPK) by using MTS assay with 24 h and 72 h of incubation time and 5 µM concentration. It was reported that, due to precipitation in cell culture media and low solubility, the curcumin did not inhibit the proliferation of leukaemia carcinoma cells. The combination of doxorubicin, curcumin and a cyclic peptide exhibited the highest inhibition rate with 78 to 84% of cell proliferation compared to doxorubicin-peptide conjugate. In the case of the ovarian carcinoma cell line and normal kidney cell line, doxorubucin-conjugated peptide exhibited the highest activity, indicating their selectivity towards the cell lines [164].

**Figure 77:**
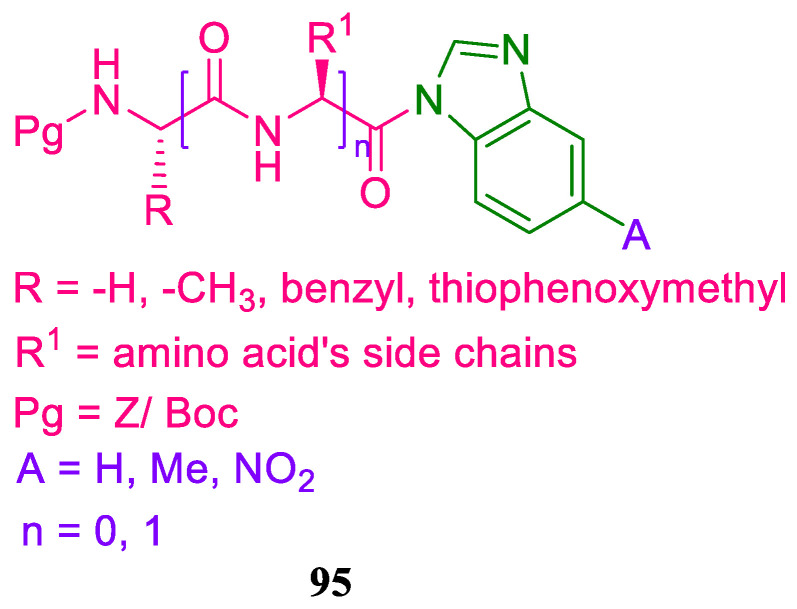
Amino acid/Peptides conjugated to benzimidazole derivatives.

Buğday N et al. synthesised a series of sixteen novel benzimidazole (**95**) conjugated to different amino acids (Gly, Ala, Phe and Cys) and a dipeptide (Gly-Gly) using DCC and evaluated their antimicrobial and antioxidant potential (Figure 77). The antimicrobial activity of the title compounds was evaluated against Gram-negative bacteria, such as *E. coli* and *P. aureginosa*, and yeasts, such as *C. albicans* and *C. tropicalis*, as well as Gram-positive bacteria, such as *S. aureus*, *E. faecium* and ampicillin, whereby ceftazidime and fluconazole served as the standard drugs. All the title compounds exhibited moderate antifungal activity, whereas some of the compounds showed the highest antifungal activity with a MIC value of 100 µg/mL. The antioxidant activity was examined for the synthesised compounds by using DPPH method, and BHA and α-tocopherol was used as the reference. All the title compounds displayed moderate free radical scavenging activity [165].

**Figure 78:**
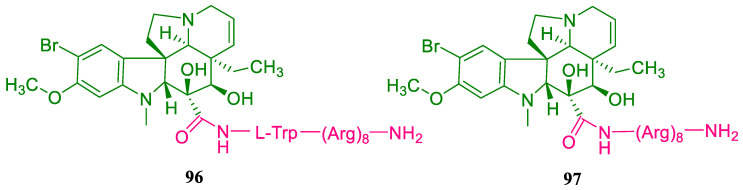
Oligoarginine conjugated to vindoline analogues.

Bánóczi Z et al. synthesised a series of vindoline analogues (**96**,**97**) conjugated to oligoarginine (tetrapeptides, hexapeptides, or octapeptides) and evaluated their antitumor activity in vivo and in vitro (Figure 78). The vindoline are the major alkaloids in plants and were first extracted from the leaves of *Catharanthus roseus*. N-terminal of octa-arginine conjugated to Br-vindoline-(*L*)-Trp-OH was the most promising compound in vitro on HL-60 cell line. Amino acid esters were coupled with the vindolines substituted at tenth position and further reacted with lithium hydroxide to obtain free carboxylic acids, which, upon hydrazinolysis, resulted in carboxylic acid hydrazide. The hydrazide product was further treated with Arg_8_-NH_2_ in the presence of sodium nitrite to obtain the title compounds. Evaluation of the in vitro activity of the two isomer conjugates, Br-vindoline-(*D*)-Trp-Arg_8_ (IC_50_ = 10.8 and 18.0 µM) and Br-vindoline-(*L*)-Trp-Arg_8_ (IC_50_ = 4.3 and 3.1 µM) indicated that the covalent bonding between vindoline and Arg_8_ resulted in the enhancement of antitumor activity against C26 and P388 tumour cells in vitro. The compounds with the highest Arg length in the oligoarginine segment showed superior activity and it was found that length of the oligoarginine is directly proportional to the IC_50_ value in HL-60 cells (i.e., octa > hexa > tetrapeptides) [166].

**Figure 79:**
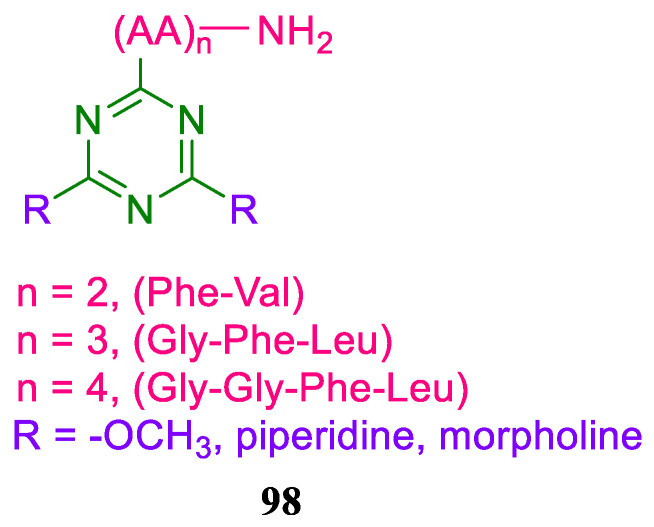
Dipeptides/tetrapeptides conjugated to s-triazine analogues.

Khattab S N et al. synthesised a library of small dipeptides/tetrapeptides conjugated to s-triazine analogues (**98**) at the N-terminus, and either amide or ethyl ester at the C-terminal, using both solution- and solid-phase methods (Figure 79). The two positions of the triazine moiety were substituted by dimethoxy or dipiperidine or dimorpholine groups. The s-triazine peptide conjugates consisted of the following amino acid sequence for dipeptides in solution-phase (Phe-Val): tripeptides (Gly-Phe-Leu) and tetrapeptides (Gly-Gly-Phe-Leu) in solid-phase. The antileishmanial activity of the synthesised compounds exhibited promising activity compared to standard miltefosine and amphotericin B. Amongst the derivatives, dimethoxy triazine conjugates exhibited greater activity than the dimorpholino and dipiperidino analogues. The conjugates with two Val amides and dimethoxy groups on the triazine moiety showed superior antileishmanial activity with an IC_50_ value of 1.4 ± 0.04 µM which was about five times than that of the standard (IC_50_ = 7.8 ± 0.34 µM) and the compound with adjacent Ala to the s-triazine showed the least activity (IC_50_ = 28.4 ± 0.22 µM). On the other hand, all the conjugates exhibited lower antipromastigote activity than the standard amphotericin B. The cytotoxicity of the synthesised conjugates was also evaluated in a VERO cell line using the Mosmann method and was found to possess the least acute toxicity [167].

**Figure 80:**
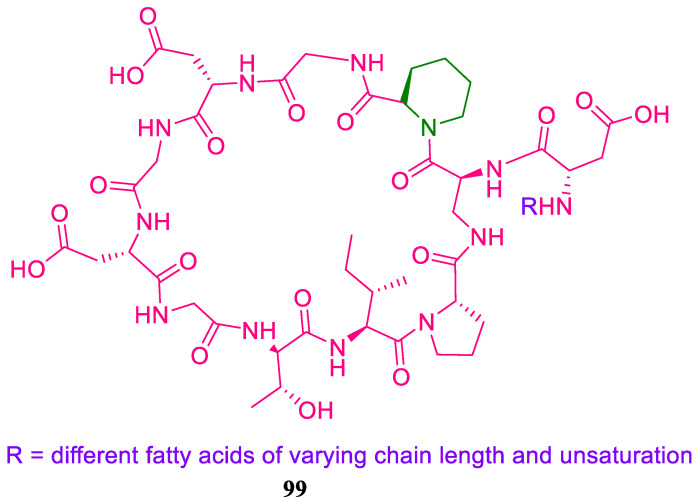
Glycinocin analogues.

Corcilius L et al. designed and synthesised a small library of glycinocin analogues of calcium-dependent antibiotics (**99**) that varied in the composition of the fatty acyl side chain (Figure 80). The cyclic peptide fragment was formed by solid-phase synthesis with a range of fatty acyl acids and then attached to glycinocin. The synthesised compounds were evaluated against both Gram-negative (*P. aeruginosa*) and Gram-positive bacteria (*B. subtilis*, *methicillin susceptible S. aureus* and *E. faecium*) using rifampicin, vancomycin, daptomycin and gentamicin as the standards. The length of the fatty acid played an important role in the biological activity and hybridisation at the α, β position and branching within the fatty acyl chain also affected the activity in large. As the fatty acyl side chain and unsaturation increased, activity also increased. The activity of the synthesised analogues was compared to the parent compound that showed similar calcium-dependent activity against Gram-positive bacteria and no activity against *P. aeruginosa*. Any of the compounds did not possess antimicrobial activity against Gram-positive bacteria or Gram-negative *P. aeruginosa* in the absence of Ca^2+^. Several conjugates showed prominent activity in the presence of 50 mg/L Ca^2+^ and a further increase in the Ca^2+^ concentration led to an increase in the potency of the conjugates [168].

**Figure 81:**
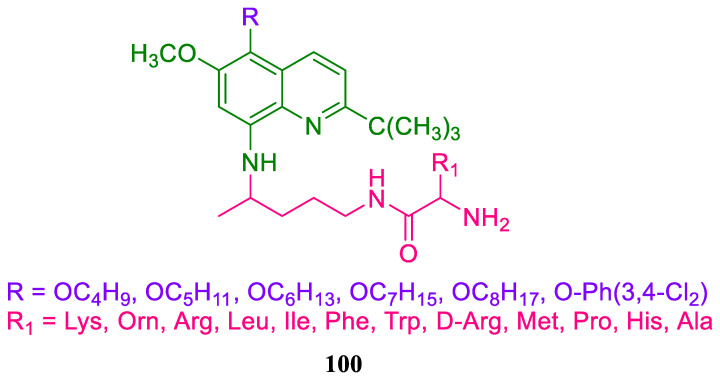
8-quinolinamines conjugated to amino acids.

Jain M et al. have synthesised a novel class of 8-quinolinamines (**100**) having 2-*tert*-butyl, 4-methyl and 5-alkoxy moieties in their quinoline skeleton and their amino acid derivatives (Figure 81). The reaction of acrolein with 4-alkoxy-6-methoxy-2-nitroanilines by Skraup synthesis using *o*-phosphoric acid and arsenic (V) oxide at 100 °C, followed by alkylation of trimethylacetic acid using silver-catalysed radical oxidative decarboxylation reaction in the presence of ammonium persulphate in 10% H_2_SO_4_ and CH_3_CN at 80 °C, yielded 2-*tert*-butyl-5-alkoxy/aryloxy-6-methoxy-8-nitroquinolines. The nitroquinolines were finally converted to 2-*tert*-butyl-4,5-disubstituted-N^8^-(4-amino-1-methylbutyl)-6-methoxy-8-quinolinamines. The synthesised conjugates were evaluated for antimalarial, antileishmanial and antimicrobial activities. The in vitro antimalarial activity was performed using an assay of plasmodial lactate dehydrogenase (LDH) activity against two different strains of *P. falciparum*, such as CQR (W2) and CQS (D6). The results revealed that among the synthesised products, conjugate N^4^-(2-*tert*-butyl-6-methoxy-5-(pentyloxy)quinolin-8-yl)-pentane-1,4-diamine bearing -H and -OC_5_H_11_ as R_1_ and R emerged as a highly efficient antimalarial agent with IC_50_ values of 22 ng/mL for W2 strain and 20 ng/mL for D6 strain, whereas the standard CQ showed IC_50_ values of 9ng/mL (W2 strain) and 11ng/mL (D6 strain). The enhanced antimalarial activity was attributed to the presence of alkoxy and 2-*tert*-butyl moieties, whereas the activity was diminished by the incorporation of methyl moiety at C-4 position of certain compounds. All the amino acid-conjugated products showed declined activity except for Arg and Lys conjugates, which exhibited good antimalarial activity but with slightly lesser activity than non-conjugated counterparts. Furthermore, the conjugates which exhibited enhanced antimalarial activity were subjected to blood-schizontocidal antimalarial activity against *P. berghei* (sensitive strain) and *P. yoelli nigeriensis* (high virulent multi-drug-resistant strain) using Swiss mice. The results revealed that compounds bearing 2-*tert*-butyl group and pentaloxy group at C-5 position of quinoline ring contributed to optimum activity, whereas the inclusion of -CH_3_ at C-4 position led to the declined activity. The authors also concluded that conjugation of Lys (a basic amino acid) increased the antimalarial activity. The cytotoxicity of the synthesised conjugates was examined by neutral red assay against a panel of four cancer cell lines, such as SK-OV-3, BT-549, KB and SK-MEL, and two non-cancer mammalian cells, such as LLC-PK1 and VERO, and reported that none of the conjugates showed cytotoxicity. The in vitro antileishmanial activity was performed using alamar blue assay against the culture of *Leishmania donovani* promastigotes and referring pentamidine as standard. The results showed that all the conjugates showed enhanced antileishmanial activities with IC_50_ values stretching between 0.84 and 5.9 µg/mL. It was noted that the title compounds synthesised by the conjugation of hydrophobic amino acids and bearing -CH_3_ and -OC_7_H_15_ inhibited the highest leishmania infection, but with the very least antimalarial activity, hence, proving it to be a highly selective antileishmanial agent. The antimicrobial studies were performed on various fungal and bacterial strains using amphotericin (antifungal) and ciprofloxacin (antibacterial) as reference standards. The antifungal activity was performed on five fungal strains such as *A. fumigatus*, *C. neoformans*, *C. krusei*, *C. glabrata* and *C. albicans*. It was observed that the conjugates having C-5-heptyloxy moiety with or without a C-4 -CH_3_ group at 8-quinolinamine skeleton showed the highest antifungal activity, whereas attachment of amino acids to the side chain of quinoline yielded the inactive conjugates. The antibacterial activity was performed on three different strains, such as *M. intracellulare*, methicillin-resistant *S. aureus* (MRSA) and *S. aureus*. It was reported that both amino acid conjugates and 8-quinolinamines retained the antibacterial activity and the selection of amino acids did not hold much impact on the activity, i.e., both hydrophilic and hydrophobic conjugates showed activity as compared to that of antimalarial and antileishmanial activities [169].

**Figure 82:**
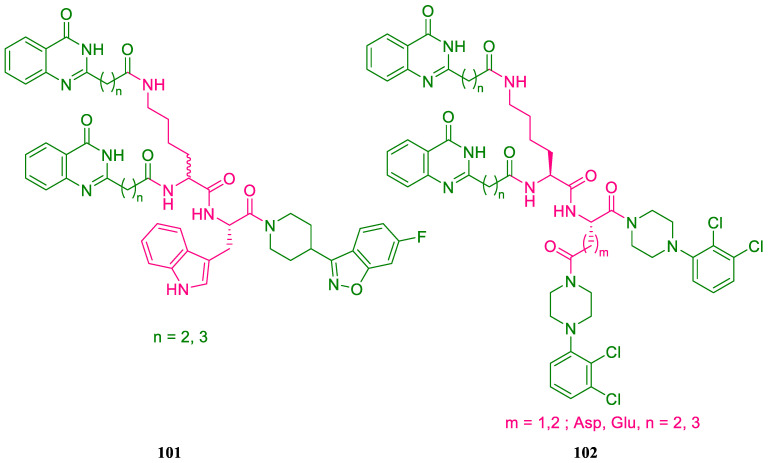
Amino acids conjugated to piperazine, benzisoxazole and quinazolinone derivatives.

Kumara H K et al. have synthesised dipeptides (**101**,**102**), i.e., KE, KD, KW, kE, kD and kW by coupling Boc-Lys(Z)-OH with HCl.H-Xaa-OPg (Xaa = Glu [OMe], Asp [OBzl] and Trp; Pg = OMe or OBzl) in the presence of NMM as base and IBCF/HOBt as coupling agent, followed by the saponification of the former product, which yielded peptides having free C-terminus end. The Cbz protecting group was deprotected by employing hydrogenolysis using 10% Pd/C, whereas Boc group was deprotected using TFA and reacted the ^Ɛ^C and ^α^C amino groups of Lys with quinazolinones, finally furnishing tetramers having various carbonyl-containing heterocyclic derivatives of piperazine, benzisoxazole and quinazolinone (Figure 82). The title compounds were evaluated for antimicrobial and anti-inflammatory activities. The antimicrobial activity was performed using agar-well diffusion and microdilution methods on different microbes, such as bacterial (Gram-negative (*E. coli*) and Gram-positive (*S. aureus*)) and fungal strains (*A. niger* and *F. moniliforme*) which, revealed that benzisoxazole conjugates showed enhanced activity with zone of inhibition and IC_50_ values of 25 ± 0.35 mm and 45 ± 0.90 µg/mL, respectively, over piperazine counterparts due to the presence of EWG (-F) and high lipophilic character of benzisoxazole. The increase in the length of alkyl chain led to the decreased activity, whereas the reverse trend of activity was observed, i.e., butyl group is preferred than propyl in the case of anti-inflammatory activity when performed using human erythrocyte suspension method. The molecular docking studies were performed on the active site of *E. coli* (PDB ID; 1KZN) protein which revealed that multimers exhibited good receptor-binding affinity with the docking score ranging between −8.059 and −4.792. The docking analysis also exhibited various interactions, such as π-cation interaction, π-π stacking interactions and hydrogen bond interaction, with various amino acid residues of the enzyme [170].

**Figure 83:**
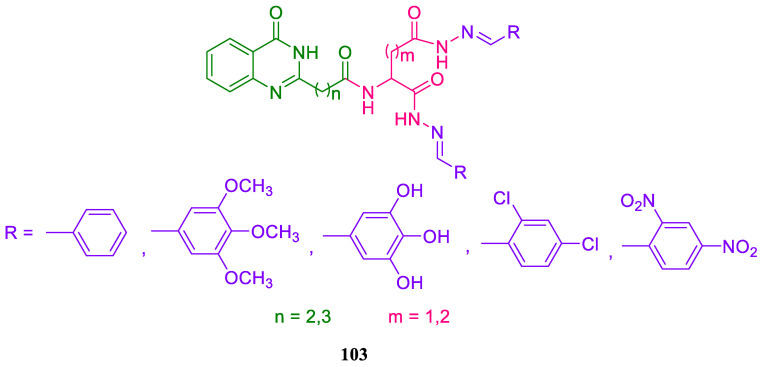
Amino acids (Glu and Asp) linked *bis*-hydrazones of quinazolinones.

Kumara H K et al. developed a new class of anionic amino acids (Glu and Asp) linked *bis*-hydrazones of quinazolinones (**103**) by the conjugation of heterocycle with *p*.TsOH.NH_2_-Asp(OBzl) and HCl.NH_2_-Glu(OCH_3_)-OCH_3_, followed by hydrazinolysis, and finally yielding *bis*-hydrazones of quinazolinone-conjugates (Figure 83). The final molecules were screened for antimicrobial, antioxidant and anti-inflammatory activities. The antimicrobial studies performed against various bacterial strains including Gram-positive (*S. aureus*), Gram-negative (*E. coli*) and fungal strains (*A. niger* and *F. moniliforme*) exhibited enhanced activity when the compounds have EDGs (-OCH_3_ and -OH) compared to standard streptomycin (antibacterial) and bavistin (antifungal). The in vitro anti-inflammatory activity was performed by human erythrocyte suspension method and it was found that molecules with EWGs, such as -NO_2_ and -Cl, exhibited better anti-inflammatory activity than the standard ibuprofen and indomethacin. The in vitro antioxidant property was evaluated by ABTS, DMPD and DPPH radical scavenging assays which showed better radical scavenging property with promising IC_50_ values for the molecules having EDGs (-OH and -OCH_3_) on the phenyl ring. In continuation, the authors have conducted molecular docking studies in order to understand the interaction of group(s) with that of the enzyme. The molecular docking was performed using the synthesised ligands on the active site of tyrosine kinase enzyme (PDB ID: 2HCK), which revealed that all the synthesised molecules exhibited better results, and especially molecules having EDG, such as -OH, which resulted in the best docking scores in the hydrogen bond interactions. It was observed from docking studies with cyclooxygease-2 that conjugates having EDGs exhibited electrostatic force of attraction with Asp13, Asp87, Lys118 and Lys188, and hydrogen bond interaction with Cys20, Lys118 and Lys118, and π-π stacking interaction with Tyr35 [171].

**Figure 84:**
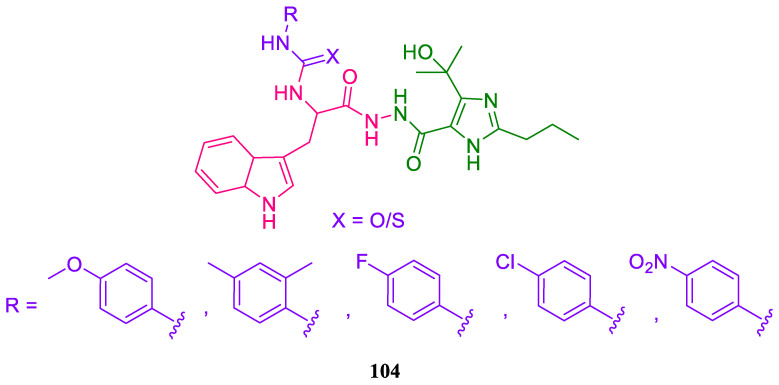
Tryptophan conjugated to imidazolo-derived thioureas/ureas.

Kumara H K et al. synthesised tryptophan conjugated to imidazole-derived thioureas/ureas (**104**) as highly potent therapeutic molecules. Hydrazinolysed version of imidazole derivative was conjugated with Boc-Trp-OH and further converted into various ureas/thioureas (Figure 84). The biological activities, such as antimicrobial, antioxidant and anti-inflammatory, were performed on the target molecules. The in vitro antimicrobial activity was performed against various fungal and bacterial strains and the results were correlated to the standards, including bavistin (antifungal) and streptomycin (antibacterial). The authors have attributed the results for the molecules, which might have better penetration ability, to the presence of a highly polar nature, hydrogen bonding, acid/base behaviour and steric property. The biological activities showed that the final urea/thioureas have presented the highest activity compared to their predecessors. The compounds having EDGs such as -CH_3_ and -OCH_3_ reported moderate activity, whereas the molecules with EWGs such as -NO_2_, -Cl and -F reported superior results compared to the standards used. The authors have also observed that due to the electronegativity of oxygen, urea derivatives showed higher antimicrobial activity than the thiourea derivatives, whereas the opposite trend was observed for antioxidant activity. Therefore, the structure–activity relationship suggested that the trend of activity is dependent on electronegativity, i.e., CH_3_ ≤ OCH_3_ <Cl<F<NO_2_. The antioxidant activity was performed by considering gallic acid and ascorbic acid as the standards. The anti-inflammatory activity was performed by using human erythrocyte suspension and it was compared to ibuprofen and indomethacin standards. The activity profile revealed that the presence of strong EWGs, such as -NO_2_ and -Cl, exhibited promising results (IC_50_ values = 30 ± 0.45 µg/mL) with higher potency than the compounds bearing EDGs (IC_50_ values = 50 ± 1.50 µg/mL). The molecular docking studies were performed on the active site of various enzymes such as tyrosine kinase, GlcN-6-P synthase and cyclooxygenase for antioxidant, antimicrobial and anti-inflammatory activities, respectively. The potentiality of the synthesised conjugates as antimicrobial entities was evaluated based on the docking scores. The molecular docking studies revealed that the molecules having EDGs exhibited the highest docking scores and emerged as potential antioxidant molecules. Most of the conjugates displayed good docking scores and binding interactions, such as hydrogen bonding with Leu273, Asp348, Asp348, Ser345, Ser354 and Ala390, and also π interaction with Gly 295. The highest docking score was observed for 2VF5 protein with the value ranging from −9.517 to −6.095 [172].

**Figure 85:**
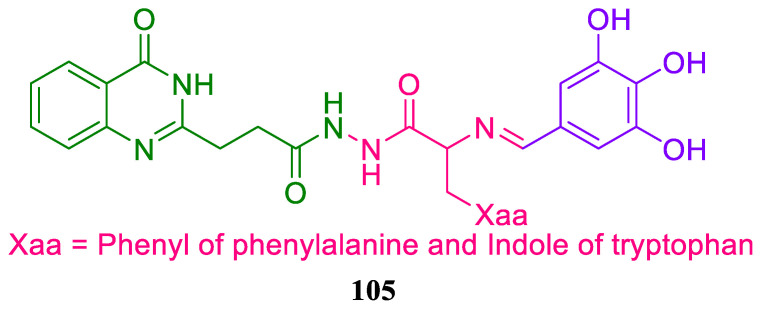
Amino acids-quinazolinone-Schiff bases.

Rakesh K P et al. synthesised a new series of quinazolinone-Schiff bases (**105**) conjugated with various amino acids and evaluated their in vitro anticancer potential against different human cancer cell lines, followed by DNA-binding and molecular docking studies (Figure 85). The anticancer activity revealed that conjugates of Phe and Trp showed the highest antitumor activity. Phe analogues showed IC_50_ values of 65.99 ± 1.51, 31.11 ± 1.19, 30.11 ± 1.08 and 29.11 ± 1.09 µg/mL, while Trp analogues exhibited 72.12 ± 0.06, 30.34 ± 1.08, 29.34 ± 0.98, 29.66 ± 0.67 µg/mL against PBMC, MCF7, MDA-MB-435 and A549 cell lines. The DNA binding studies revealed that Phe and Trp conjugates reported the highest activity with IC_50_ values of 48.33 ± 0.18 and 48.36 ± 1.39 µg/mL. From the SAR studies, the conjugates with amino acids, Phe and Trp and the presence of EDGs, such as -OH and -OCH_3_, presented enhanced anticancer activity with good binding capacity with DNA, whereas the molecules having EWGs, such as -F, -Cl and -NO_2_, reported much less anticancer activity. The molecular docking studies revealed that conjugates of Phe and Trp showed the highest docking scores of −7.948 and −7.400, respectively [173].

**Figure 86:**
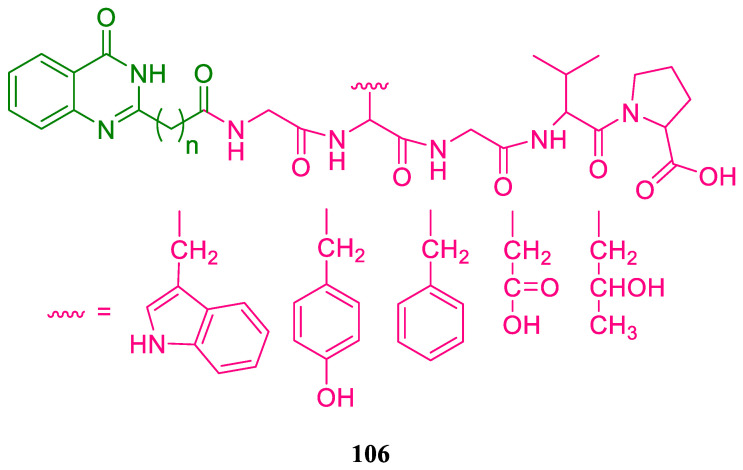
Peptides conjugated to quinazolinones.

Rakesh K P et al. synthesised two novel series of peptides conjugated to quinazolinones (**106**) (QZNs-Gly-X-Gly-Val-Pro-OBzl/OH) by varying different amino acids at the second position of the peptide (i.e., X position) in the GXGVP peptide template (Figure 86). Amino acids used in the X position possessed different charge, polarity and hydrophobicity. The new conjugates were evaluated as potent antioxidant and anti-inflammatory agents. Among the synthesised molecules, those having Asp at the second position of the peptide chain and free carboxyl C-terminus with butyl group in the heterocyclic part was reported to be a good anti-inflammatory agent (IC_50_ = 20 ± 0.65 µg/mL). On the other hand, molecules having Trp residue at the X position of the peptide chain turned out to be highly potential radical scavengers (IC_50_ = 25 ± 1.18 µg/mL). The authors have also suggested that the molecules having charged species reduces inflammation property, whereas molecules with higher hydrophobicity favours the antioxidant property. Furthermore, the lead molecules having butyl group in the heterocycle and with free C-terminus in the peptide moiety reported good anti-inflammation property [174].

**Figure 87:**
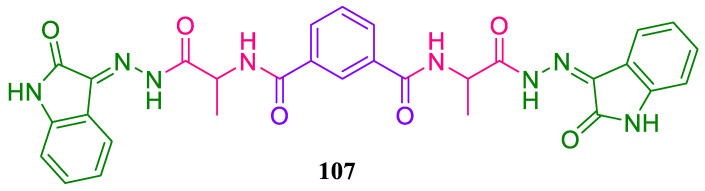
N^1^,N^3^-*bis*-(1-oxopropan-2-yl) isophthalamide-based derivatives.

Kassem A F et al. have synthesised new N^1^,N^3^-*bis*-(1-oxopropan-2-yl) isophthalamide-based derivatives (**107**) using solution-phase method and evaluated for anticancer activity using MTT assay and doxorubicin as reference standard against lung carcinoma (A-549), human colon carcinoma (HCT-116) and human breast carcinoma (MCF-7) cell lines (Figure 87). The structure–activity relationship studies reported that the conjugates synthesised by the cyclisation of 2-oxoindoline group reported a more enhanced anticancer activity, with improved IC_50_ values of 0.040 ± 1.43, 0.014 ± 0.22 and 0.014 ± 0.70 µM against A-549, HCT-116 and MCF-7 cancer cell lines, respectively, than doxorubicin, whereas the reverse trend of action was observed in certain molecules having chlorine atom substituted at p-5 of 2-oxoindoline. The other counterparts synthesised by the cyclisation of 1,3,4-oxodiazole reported high sensitivity towards MCF-7 cell line with 77.5% inhibition rate. The compounds synthesised by the conjugation of 2,3,4,5-tetrahydroxypentyl sugars with isophthalamide reported decreased anticancer activity. Introduction of EDGs (-OCH_3_) or EWGs (-F and -Cl) at p-4 of the phenyl ring in the title compounds revealed noticeable and variable increased cytotoxic activity, whereas decreased cytotoxic activity was observed due to steric hindrance caused by two methoxy groups substituted at p-3,4 of the phenyl ring. The derivatives of 4-oxothiazolidine joined at p-2 of chlorophenyl reported a more enhanced anticancer potency than 4-methoxyphenyl and 4-fluorophenyl counterparts. Furthermore, the newly synthesised compounds with promising anticancer properties were further evaluated for in vitro enzymatic assays in which inhibitory activities of four different kinases was examined, such as c-kit (receptor tyrosine kinase type III), cyclin-dependent kinase-2 (CDK-2), vascular endothelial growth factor receptor-2 (VEGFR-2) and epidermal growth factor receptor, by using multitarget inhibitor. It was reported that the highly potent title compounds synthesised by the cyclisation of 2-oxoindoline moiety reported better inhibitory activity of EGFR kinase than the reference standard, whereas the least inhibitory activity was reported for the other three tested enzymes. The molecular docking studies was performed on the active site of EGFR enzyme in order to study the inhibitory activity and possible binding interactions on the synthesised compounds. It revealed that isophthalamide derivative having 2-oxoindoline group reported to have made two hydrogen bonds with similar amino acid Met769 as erlotinib [175].

**Figure 88:**
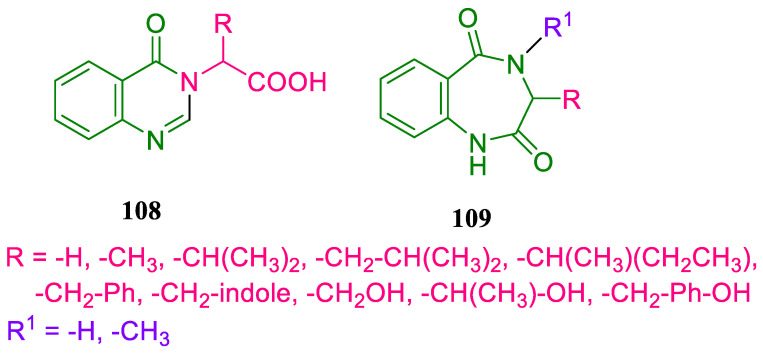
Quinazolinones and 1,4-benzodiazepine-2,5-diones conjugated to amino acids.

Anil S M et al. synthesised quinazolinones (**108**) and 1,4-benzodiazepine-2,5-diones (**109**) from amino acids and tested their antitubercular activity (Figure 88). The quinazolinones and 1,4-benzodiazepine-2,5-diones with different substituents at the C-3 position were synthesised by using isatoic anhydride and L-amino acid methyl ester in the presence of H_2_PtCl_6_ catalyst and were evaluated for antimycobacterial tuberculosis activity. Among the synthesised compounds, those derived from Pro and Trp showed significant activity, which may be due to the presence of active indole or fused pyrrolidine moieties, respectively. The compound 1,4-benzodiazepine-2,5-dione derived from Trp showed superior activity with docking score −10.58 kcal mol^−1^ and has a MIC value of 1.55 µg/mL. The authors concluded that future research on active anti-TB medication candidates might be enabled by these compounds. Molecular docking was performed to enhance the mode of action, interaction and preferred binding sites of the targeted chemicals with the active areas of the enoyl acyl carrier reductase, a bacterial protein (InhA, PDB, ID-2NSD). The synthesised compounds were docked using the GLIDE module of Schrödinger on enoyl acyl carrier reductase (InhA, PDB ID-2NSD) of Mtb. The docking scores of all the synthesised compounds ranged from −6.003 to −11.1. Among the synthesised molecules, conjugates bearing indole moiety exhibited the highest docking score of −11.1 due to π-π staking interaction of the aromatic ring of diazepine-2,5-dione with Tyr158 of the bacterial protein. SAR studies showed that substitution at the C-3 position increased the activity, which may be due to the hydrophobicity of the molecule. These non-peptide, biocompatible, amino acid-derived peptidomimetics open a new door in the design of anti-TB drugs by offering a broad spectrum of potential therapeutic targets and several extremely targeted, non-toxic boost candidates [176].

**Figure 89:**
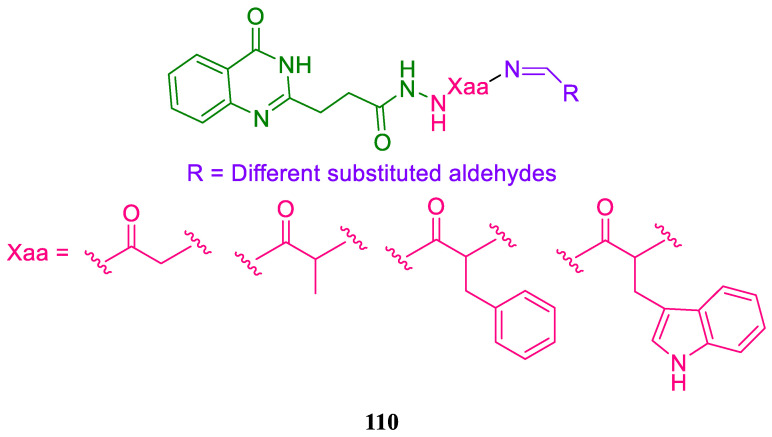
Amino acids conjugated to quinazolinone-Schiff bases.

Rakesh K P et al. synthesised a novel series of amino acid-conjugated quinazoline-Schiff bases (**110**) to treat fungal and bacterial diseases (Figure 89). The synthesised quinazolinone-Schiff’s base conjugates were evaluated for their in vitro antimicrobial activity against various bacterial and fungal strains by using streptomycin and bavistin as reference standards. The bacterial strains comprises both Gram-positive (*S. aureus*) and Gram-negative (*E. coli*), while the fungal strains include *F. oxysporum* and *A. niger*. The molecular docking studies was performed on the active site of GlcN-6-P synthase enzyme. It was observed that the title compounds exhibited good binding interaction with surrounding amino acid moieties. A majority of the conjugates exhibited well-established bonds with more than one amino acids in the receptor active pocket of 2VF5 protein. The potentiality of the conjugates as antimicrobial entities was evaluated based on docking score. The highly potent compounds formed a hydrogen bond interaction with Gln348, Ala602, Ser303, Gly301, Ser347, Ser349, Gln348, Lys603, Ala602 and Glu488, which is vital for a substrate to bind with 2VF5. The docking scores of the title compounds ranged from −4.048 to −10.504. The conjugate possessing two EDGs, viz. hydroxyl groups, exhibited the highest docking score of −10.504. Molecular docking studies, in vitro antimicrobial activity, and preliminary structure–activity relationship (SAR) studies revealed that conjugates containing Phe and Trp with EDGs such as -OCH_3_ and -OH in their phenyl ring exhibited better antibacterial activity than Gly- and Ala-containing conjugates against *E. coli* and *S. aureus*, compared to standard streptomycin. The compounds with EWGs such as -NO_2_, -F and -Cl performed better as antifungal agents [177].

**Figure 90:**
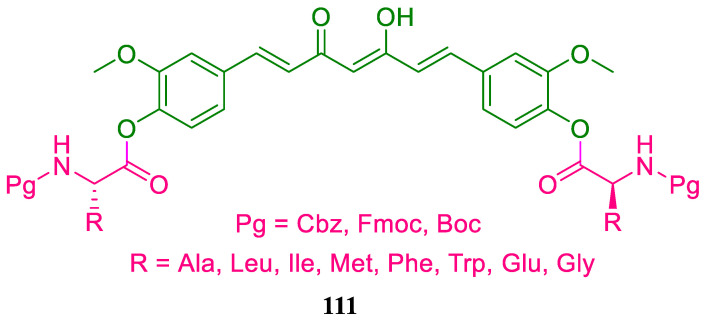
Curcumin *bis*-conjugates (**111**) of different N-protected amino acids.

Panda S S et al. have synthesised a novel series of curcumin *bis*-conjugates (**111**) of different N-protected amino acids in the presence of DMAP and EDAC, and later developed them as highly potent antimicrobial, anti-inflammatory and analgesic agents (Figure 90). The authors used Fmoc and Cbz as amine protecting groups in order to explore the effect of various protecting groups on biological properties. The products obtained by using Fmoc and Cbz were impure and they did not retain the chiral integrity, whereas the products obtained by using Boc-protecting group were highly pure with good chirality retention. The antimicrobial properties of the title compounds along with curcumin were examined against various bacterial (including both Gram-negative (*P. aeruginosa* and *S. typhi*) and Gram-positive (*S. pyogenes* and *S. aureus*)) and fungal strain (*C. albicans*). The results revealed that the synthesised molecules (MIC = 0.113 to 0.166 µM) reported enhanced activity nearly two folds to that of curcumin (MIC = 0.339 µM). The antifungal activity of curcumin (MIC = 0.339 µM) also exhibited a more enhanced activity than the standard, amphotericin (MIC = 0.422 µM). Among the synthesised molecules, only one compound reported to be highly efficient compared to the standard and parent curcumin with a MIC of 0.214 µM. The anti-inflammatory activity was performed using acute carrageenan-induced paw edema technique and indomethacin and ibuprofen were used as standards. The structure–activity relationship studies reported that Fmoc-protected amino acid conjugates exhibited a more enhanced anti-inflammatory activity than Boc- and Cbz-protected amino acid counterparts. Among the various amino acids used for the synthesis, derivatives of Met emerged as potent anti-inflammatory agents compared to the derivatives of alkyl-substituted amino acids at the α-position, such as Val, Ala and Leu. The authors further correlated the results of anti-inflammatory properties with the production of NO by lipopolysaccharide-stimulated peritoneal macrophages, and also the effect of synthesised molecules on splenocytes. The analgesic properties of the synthesised conjugates were examined by using in vivo acetic acid-induced abdominal writhing technique in mice and hot plate test. It was observed that those conjugates which exhibited high potential anti-inflammatory activity were also effective as central and peripheral analgesic agents. Thus, the authors concluded that the conjugation of amino acids with curcumin might enhance either peripheral or central analgesic properties. Furthermore, the title compounds were examined for the ulcerogenic liability and toxicological bio-assay, by which it was concluded that conjugates showed no lesions or erosions and ulcers to the tested animal, and finally confirming it for the safety use with zero toxic and side effects. The computational ADME (absorption, distribution, metabolism and excretion) studies showed that the synthesised conjugates revealed plasma protein binding levels >95% [178].

**Figure 91:**
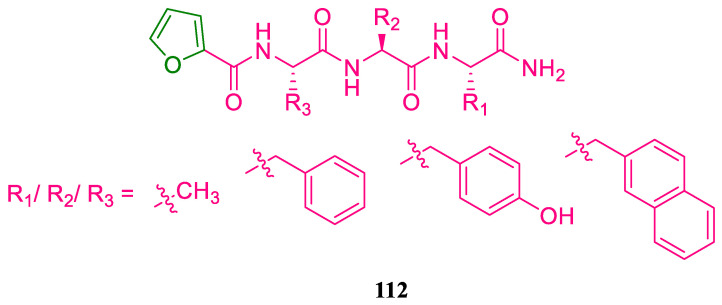
Furan-conjugated tripeptides.

Ali H et al. have synthesised a series of furan-conjugated tripeptides (**112**) by using rink amide resin and developed them as anticancer agents with high specificity only towards the human cervical cancer cells (HeLa cells) and zero proliferation towards other human cancer cells (MDA-MB-231, HUVEC and MCF-7), normal human cells (human umbilical vein endothelial cells), normal human fibroblasts (IMR-90) and tested mice 3T3 cells (normal fibroblasts) (Figure 91). The peptides were constructed from C- to N-terminus on rink amide resin and later capped them by α-furoic acid moiety. From the structure–activity relationship studies it was reported that among the synthesised title compounds, Fur^4^-2-Nal^3^-Ala^2^-Phe^1^-CONH_2_ emerged as a highly potent anticancer agent against HeLa cells (human cervical cancer cells) with a promising IC_50_ value (0.28 ± 0.09 µM). Furthermore, two dendrimeric forms of the highly potent conjugates were synthesised at C-terminus by linking jeffamine linker or Lys residue to each pair of monomeric conjugate. It was evaluated for antiproliferative activity against HeLa cells and was reported to be inactive (IC_50_ > 100 µg/mL), and was finally concluded that in order to be active against HeLa cells, the C-terminus of the conjugate has to be free in the form of amide. From the atomic force microscopy (AFM) studies, it was reported that conjugate Fur^4^-2-Nal^3^-Ala^2^-Phe^1^-CONH_2_ was able to retain membranolytic effect and induced the loss of mitochondrial membrane potential [179].

**Figure 92:**
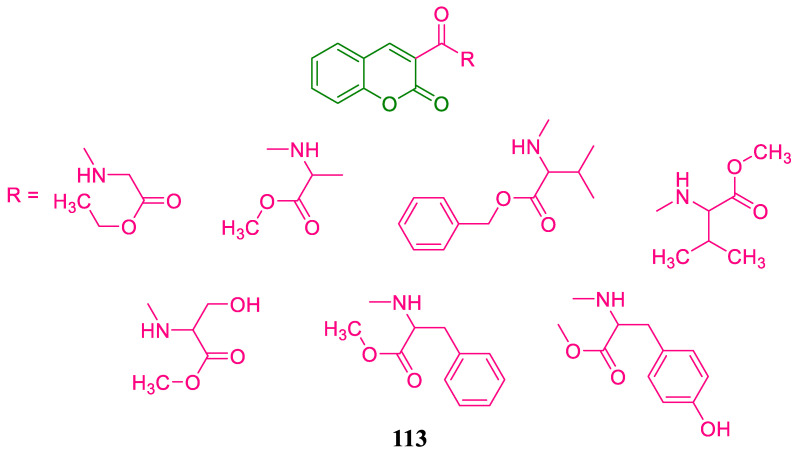
Coumarin-amino acid derivatives.

De Naik M et al. have developed an efficient one-pot synthesis for coumarin-amino acid derivatives (**113**) and evaluated as potential antioxidant and anti-inflammatory agents by coupling various L-configured amino acid methyl esters of Tyr, Phe, Ser, Leu, Val, Ala and Gly (Figure 92). The antioxidant activity involved DPPH free radical and nitric oxide scavenging activities. From both the activities, it was reported that molecules synthesised by coupling with Ser and Tyr emerged as highly potent radical scavengers with a promising IC_50_ value of 28.23 and 31.45 µg/mL when compared to the reference standard (ascorbic acid, IC_50_ = 20.53 µg/mL), which was attributed to the presence of aliphatic hydroxyl group of Ser and proton-donating ability of phenolic -OH of Tyr, whereas Gly-conjugated derivatives reported the very least rate of activity. The anti-inflammatory activity was performed using protein denaturation by both bovine serum albumin and egg albumin. In protein denaturation by bovine serum albumin method, it was reported that conjugates of Phe exhibited highly potent anti-inflammation activity compared to the reference standards, whereas Tyr conjugates exhibited the least activity that could be due to the presence of hydrophilic phenolic moiety. The conjugation of coumarin with aliphatic amino acids containing non-polar alkyl group and polar alcoholic moiety, such as Gly, Ala and Ser, reported moderate to good inhibition activity. In the case of protein denaturation by egg albumin denaturation method, aliphatic amino acid esters of Leu and Ser exhibited enhanced activity. Specifically, Ser conjugates exhibited the highest activity, whereas coumarin-3-carboxylic acid conjugated with aromatic amino acid esters of Phe reported moderate activity. Finally, the authors concluded that Tyr-containing conjugates reported the least inhibition of protein denaturation [180].

**Figure 93:**
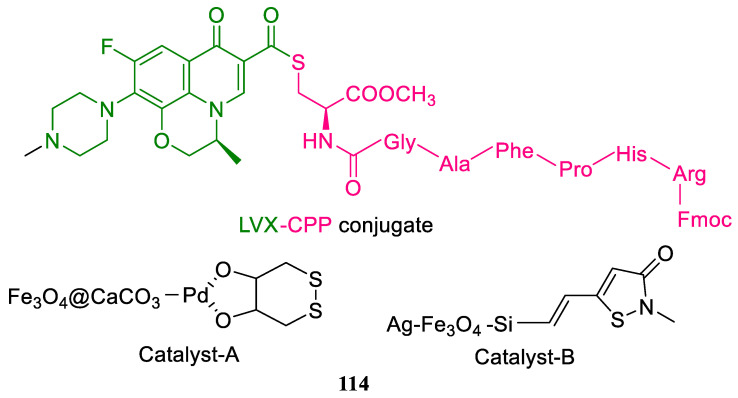
Levofloxacin (LVX) conjugated cell penetrating peptides.

Taheri-Ledari R et al. synthesised a series of levofloxacin (LVX) conjugated to cell penetrating peptides (CPP) consisting Cys-Gly-Ala-Phe-Pro-His-Arg (**114**) by the ultrasonication (ultrasound wave) method and evaluated the antimicrobial activity (Figure 93). In this process, cysteine was used as the linker between CPP and LVX. The CPP chain formation started with Gly-Ome and Fmoc-Ala-OH in the presence of sono/nano catalyst, such as Fe_3_O_4_Pd/CaCO_3_-DTT and Ag-Fe_3_O_4_ and DIPEA by ultrasonication. On the other hand, LVX was treated with Fmoc-protected N-Cys in the presence of DCC/NHS/DIPEA/DMF in N_2_ atmosphere. Further, ultra-sonification with piperidine followed by magnetic catalysis using Fe_3_O_4_Pd/CaCO_3_-DTT and Ag-Fe_3_O_4_ produced CPP-LVX conjugate. It was observed that CPP-LVX conjugates showed nearly twenty times an increase in the antimicrobial activity (20 µg/mL) by optical density and zone of inhibition (1.86 to 2.48 cm) methods. Arg present in the terminus of the peptide chain having guanidine functional group might be involved in the electrostatic interaction between the negatively charged phosphate group in the cell membrane and positively charged amine group in the guanidine structure, that would have played an important role in the CPP’s high activity [181].

**Figure 94:**
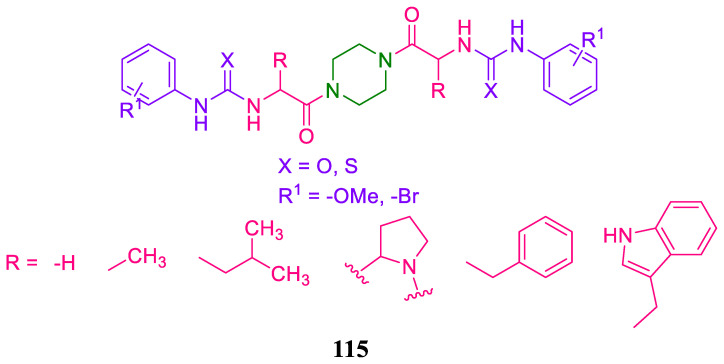
Piperazine-bridged pseudopeptidic thiourea/urea derivatives.

Anil S M et al. have designed and synthesised a novel series of piperazine bridged pseudopeptidic thiourea/urea derivatives (**115**) by utilising the information of molecular docking studies and developed the title compounds as high potential radical scavengers (Figure 94). Initially, Boc-protected amino acids were coupled with piperazine in the presence of HBTU/TEA, which resulted in the intermediates, followed by deprotecting the Boc moiety, resulting in hydrochloride salt, which, on further reaction with isothiocyanates and isocyanates, furnished the desired title compounds. The title compounds along with the intermediates were screened for in vitro antioxidant activity by using DMPD and DPPH radical scavenging assays with gallic acid and ascorbic acid as standards. The results revealed that the intermediates exhibited radical scavenging property at higher concentrations, among which the Trp and Phe intermediates showed an enhanced activity, which may be due to indole side chain and aromatic phenyl ring, respectively. The improved antioxidant property was observed by the conversion of intermediates into multi-thiourea/urea analogues due to the ability of potential radical scavenging -NH-(CO/S)-NH- moieties. The conjugates having electron-donating thiourea fragment in the aromatic side chain exhibited the highest radical scavenging activity. The molecular docking studies of the synthesised conjugates was performed on the active site of tyrosine kinase. The results revealed that the protein analogues are involved in the hydrogen bonding interactions with the ligands, such as Leu273, Ala275, Gln277, Lys295, Phe278, Ser345, Asp348, Asp386, Arg388, Ala390, Asp404, Lys423 and Asn391, and they are also involved in π-cation/π-π stacking interactions with Phe424, Phe278 and Arg388. The docking scores of highly potent conjugates ranged from −5.037 to −9.331. Finally, in the structure–activity relationship studies, it was concluded that thioureido analogues were found to be active and exhibited a more enhanced activity than the ureido counterparts, which could be due to the divalent bio-isosteric effect of sulphur atom [182].

**Figure 95:**
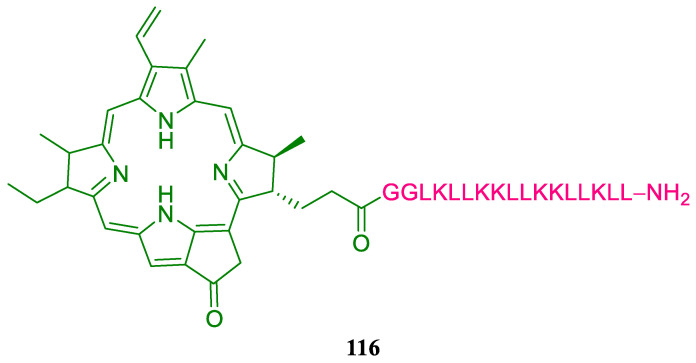
PPA conjugated to antimicrobial peptide.

Song M et al. designed and synthesised pyropheophorbide-a (PPA) conjugated to antimicrobial peptide (**116**) [K6L9] to increase the anticancer activity and decrease the toxicities such as the hemolysis and organ dysfunction of antimicrobial peptide (Figure 95). Antimicrobial peptides (AMPs) are mainly anti-infective agents due to their membrane lytic property and these are considered as good antitumor agents. However, their anticancer applications are mainly hindered by high systemic toxicities and fast renal clearance. This paper is based on a strategy aiming at addressing these two queries by conjugating antimicrobial peptides with porphyrins. PPA is a sensitiser which has the ability to increase the antitumor effectiveness of peptides by using photodynamic effect. The conjugate was evaluated for the anticancer effects in 4T1 cell line. In this study, the authors confirmed that PPA, as a novel albumin-targeting molecule, exhibited higher binding affinity. The authors observed that the PPA-conjugated AMP bound to albumin at the sub-micromolar range was due to an increase in anticancer activity with the IC_50_ value ranging between 0.13 and 6 µM, as well as an exhibited reduction in hemolysis and hepatic injuries, which prolonged in vivo retention time. This study suggested that PPA-conjugation can increase the AMPs’ resistance against renal clearance, thereby increasing the anticancer effects in vivo [183].

**Figure 96:**
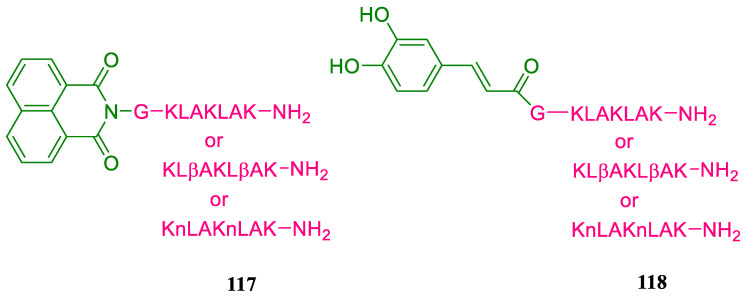
Analogues of (KLAKLAK)_2_-NH_2_ and their derivatives.

Jaber S et al. synthesised new shortened analogues of (KLAKLAK)_2_-NH_2_ and their derivatives containing non-natural amino acids (**117**,**118**). (KLAKLAK)_2_ is a representative of the antimicrobial peptide group which also exhibited good anticancer properties (Figure 96). The general structure of the shortened analogues of (KLAKLAK)_2_ as C-terminal amides was Lys-X-Y-Lys-X-Y-Lys-NH_2_, where Y is Ala or β-Ala (β-A) and X is Leu or nor-Leu (nL). Further, the peptides were conjugated to second pharmacophore with the best proven anticancer properties, i.e., caffeic acid and 1,8-naphthalamide. The antiproliferative activity of the conjugates was evaluated in cell cultures of various human cell lines, viz. mammary gland type A adenocarcinoma ER+, PR+, HER2-(MCF-7), triple-negative breast cancer ER-, PR-, HER2- (MDA-MB-231) and breast, non-tumorigenic epithelial cell line, using the standard MTT-dye reduction assay in which the conjugates showed moderate antiproliferative activity. The antibacterial properties of the newly synthesised conjugates were evaluated against facultative anaerobic Gram-negative microorganism such as *E. coli* at two different concentrations of 10 µM and 20 µM using agar diffusion method. The results revealed that there was a significant selective index for the conjugates with structure KLβAKLβAK-NH_2_. Conjugation of (KLAKLAK)_2_ with 1,8-naphthalimideGly and caffeic acid enhanced the antiproliferative activity and cytotoxicity, but not their selectivity. 1,8-naphthalimideGKnLAKnLAK-NH_2_ and KLAKLAK-NH_2_ were the only two compounds that exhibited moderate activity against *E. coli* at 20 µM. The substitution of natural with unnatural amino acids made the resulting peptides complicated to be identified as substrates by the enzymes responsible for hydrolysis in the human body. This might have led to an enhancement in the half-life as well as the hydrolytic stability of the final compounds [184].

**Figure 97:**
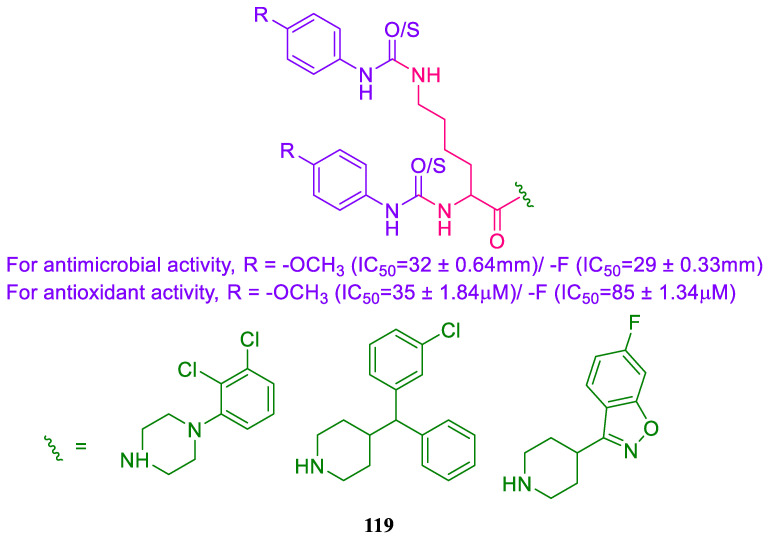
Lys-conjugated heterocycles.

Pavan Kumar H et al. reported the synthesis of Lys-conjugated heterocycles having *bis*-thiourea/urea pendants (**119**) and developed them as potential antimicrobial, antioxidant and anti-inflammatory agents. Boc-Lys (Boc) was used as the starting material and was conjugated with three different heterocycles, viz. (3-(4-piperidyl)-6-fluoro-1,2-benzisoxazole).HCl, 1-(2,3-dichlorophenyl)piperazine and chloro-4-benzhydryl piperidine (Figure 97). Deblocking of Boc groups followed by treatment with different substituted isocyanates/isothiocyanates furnished thiourea/urea derivatives, respectively. The in vitro antimicrobial activity was performed using agar-well diffusion and microdilution methods against various bacterial (both Gram-negative (*E. coli*) and Gram-positive (*S. aureus*)) and fungal strains (*F. verticillioide* and *A. niger*) and the results were compared with the standards, gentamycin (antibacterial) and bavistin (antifungal). The antimicrobial studies revealed that those molecules with EDGs (32 ± 0.64 mm) exhibited excellent antimicrobial activity compared to the standards, whereas the molecules with EWGs (29 ± 0.33 mm) reported the least activity. The antioxidant activity (DPPH, ABTS, DMPD) of all the synthesised molecules revealed that the molecules containing EDGs (35 ± 1.84 µM/mL) and isoxazole heterocycle were better radical scavengers compared to the molecules having EWGs (85 ± 1.34 µM/mL). It was also reported that thiourea conjugates emerged as better radical scavengers over urea counterparts, thus revealing the significance of S over O for the potential radical scavenging property. The anti-inflammatory activity was performed using human erythrocyte suspension and the results revealed that the title compounds having EWGs reported more enhanced anti-inflammatory activity with IC_50_ values of 40 ± 2.34 µg/mL than the standards, ibuprofen and indomethacin. In particular, the compounds possessing isoxazole unit exhibited enhanced activity nearly two times higher than the rest of the synthesised compounds. The molecular docking studies were performed on the active site of 2VF5 protein, which revealed that certain conjugates interacted with proteins by π-π stacking and hydrogen bonding which also possessed good binding potency between enzyme and the ligand. Finally, the authors concluded that the existence of (thiourea/urea)_2_ induced the enhanced activity, specifically the conjugates substituted by 4-F and 4-Ome as they offered the most potent results [185].

**Figure 98:**
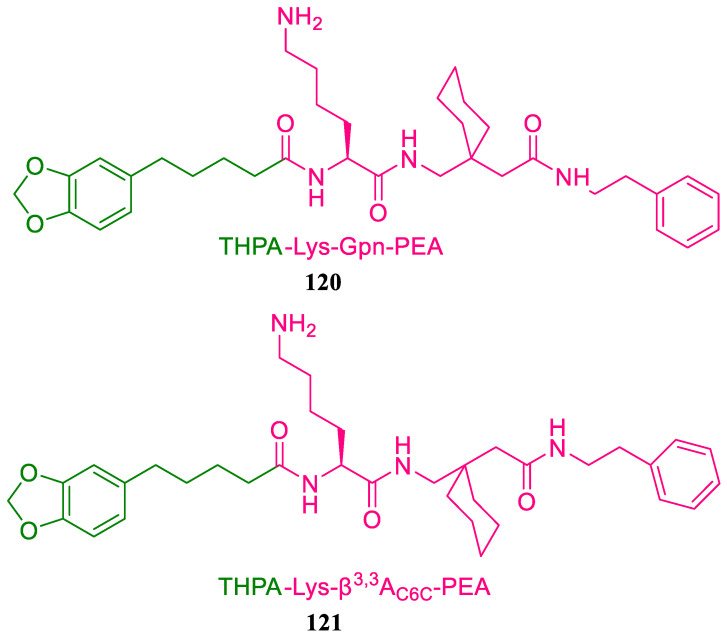
Dipeptides conjugated to THPA.

Ur Rahim J et al. synthesised a series of cationic hybrid dipeptides conjugated to tetrahydropiperic acid (THPA) and tested their antimicrobial activity (Figure 98). The synthesised hybrid dipeptides were H-Lys-Gpn-PEA (**120**) and H-Lys-β^3,3^A_C6C_-PEA (**121**). Hydrolysis of piperine produced piperic acid, followed by catalytic hydrogenation to yield THPA. The peptides were synthesised in solution phase in the presence of EDCI/HOBt/NMM. All the compounds were tested against both Gram-negative *P. aeruginosa*, *E. coli*, *K. pneumoniae* and *S. typhimurium*, and Gram-positive *S. aureus* and *B. subtilis* bacterial strains. Among the synthesised compounds, THPA-Lys-β^3,3^A_C6C_-PEA showed significant activity with a MIC of 1.56 µM. THPA-conjugated peptides showed significant antimicrobial activity, whereas the dipeptides without lipid chain at the N-terminus showed negligible antimicrobial activity. THPA-Lys-β^3,3^A_C6C_-PEA having Lys showed superior antibacterial activity against *S. aureus* and *P. aeruginosa* with MBC and MIC values of 6.25 and 1.56 µM, respectively, along with traces of cytotoxicity and lower hemolytic activity against normal human breast cell lines. The authors performed the fluorescent staining experiment using propidium iodide (PI) and 4,6-diamidino-2-phenylindole (DAPI), as well as the SEM studies, and confirmed the bacterial membrane disruption by the conjugates. Further, the bacteria killing kinetics was evaluated against *S. aureus* and *P. aeruginosa* to understand the mechanism of action [186].

### 2.2. Amino Acids and/or Peptides Conjugated Bioactive Molecules (Other than Heterocycles)

**Figure 99:**
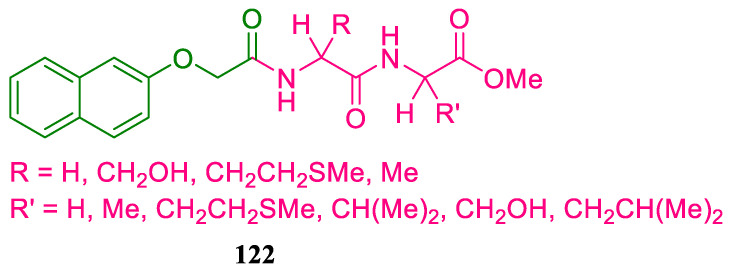
Peptide derivatives conjugated to naphthalene moiety.

Al-Masoudi N A et al. synthesised a novel series of peptide derivatives (**122**) conjugated to naphthalene moiety, glycoside, 7-glycoside and 2-(2-hydroxy-3-(N-benzyl-N-isopropylamino)propoxy)naphthalene having Met, followed by screening for anti-HIV, antitumour and bovine viral diarrhoea virus (BVDV) activities (Figure 99). The structure–activity relationship studies of in vitro anti-HIV assay revealed that the conjugates having highly potent β-adrenergic blocking moiety reported a more enhanced anti-HIV activity than the corresponding derivatives having amino acids only. The in vitro cytotoxicity was examined against a panel of cancer cell lines consisting of CD4 human acute T-lymphoblastic leukaemia, T-leukaemia virus type 1 (HTLV-1), acute B-lymphoblastic leukaemia, splenic B-lymphoblastoid cells, melanoma, lung squamous carcinoma, breast adeno carcinoma, prostate carcinoma, hepatocellular carcinoma, lung fibroblasts and foreskin fibroblasts using MTT assay. From the in vitro cytotoxic activity, it was observed that Gly-Leu analogues reported remarkable activity with CC_50_ value of 16 µM against MT4 cell line and CC_50_ value of 20 µM against CCRF-CEM cell line. Finally, it was concluded that the treatment of leukaemia can be successfully achieved by the alteration of the conjugated functional groups of Gly-Leu peptide derivative, respectively [187].

**Figure 100:**
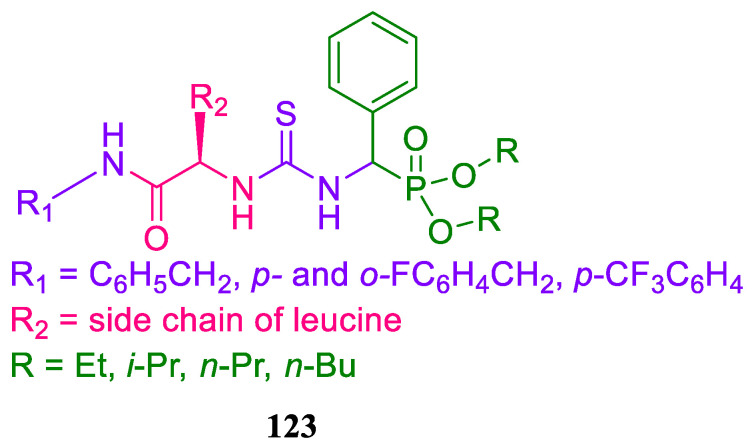
α-amino carboxamide derivatives.

Liu J Z et al. synthesised a series of novel chiral thioureas containing Leu and phosphonate analogues (**123**) in good yields. The treatment of *L*-N-Boc-Leu with a substituted arylamine in the presence of HBTU, followed by deprotection, yielded α-amino carboxamide derivatives (Figure 100). *O*,*O*′-dialkylisothiocyanato (phenyl)methylphosphonates were obtained by a series of reactions starting from benzaldehyde. The coupling of *O*,*O*′-dialkylisothiocyanato (phenyl)methylphosphonates and α-amino carboxamide derivatives produced desired chiral thiourea analogues. The title conjugates were evaluated for their antiviral activity with ningnanmycin as the standard. The report displayed that the title conjugates showed average to good anti-TMV activity. The compounds containing (R_1_ = *p*-F-C_6_H_4_CH_2_, R_2_ = *i*-Bu, R = *i*-Pr) and (R_1_ = *p*-CF_3_-C_6_H_4_, R_2_ = *i*-Bu, R = *i*-Pr) exhibited good biological activity with curative rates of 56.7 and 53.6% against TMV, respectively, compared to their parent analogues. The compounds containing EWGs, such as -F and -CF_3_ at the para position of the aromatic ring, reported better anti-TMV activity with inhibition rates of 48 to 56.7%. Compounds with *L*-Leu showed superior activity than its *D*-enantiomer and racemate. This work indicated that the anti-TMV activity of chiral thiourea was remarkably enhanced by the incorporation of appropriate *L*-configured substituted derivatives [188].

**Figure 101:**
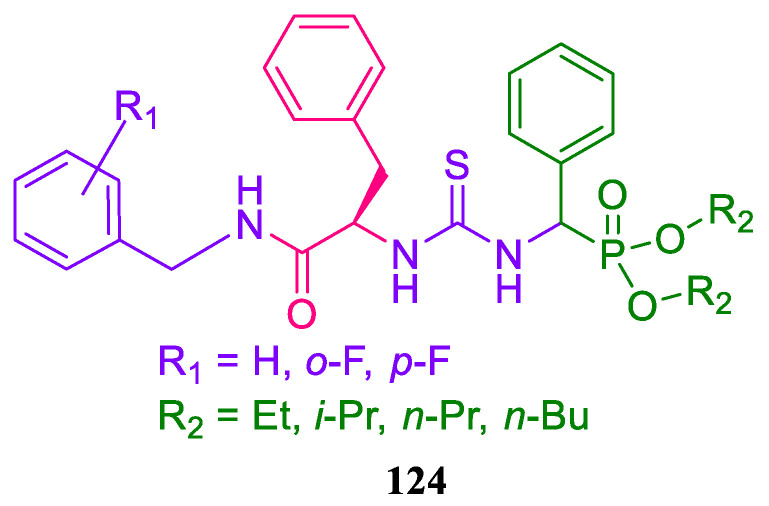
α-aminophosphonate analogues.

Liu J Z et al. synthesised twenty pseudo-peptide thioureas consisting α-aminophosphonate analogues (**124**). The title conjugates were obtained by the coupling of α-amino carboxamide derivatives with *O*,*O*′ dialkylisothiocyanato(phenyl)methylphosphonate and were evaluated for their antitumor activities against human cancer cell lines (Bcap-37, BGC-823 and PC-3), prostate cancer, stomach cancer and breast cancer cell lines (Figure 101). Among the synthesised compounds, *L*-configured conjugates with *p*-F, *i*-Pr and *D*-configured conjugates containing H, Et, *o*-F were found as potent inhibitors with IC_50_ values ranging from 4.5 to 11.3 µM by in vitro MTT assay. Compounds with *D*-diastereomers showed greater activity than *L*-diastereomers. The presence of fluoro at the para position of benzyl ring connected to nitrogen groups increased the anticancer activity of the compounds. The type of amino acids, the configuration of α-amino acids and the alkyl structure of the phosphonates in the title conjugates played an important role in the anti-proliferative activity of the cancer cell lines [189].

**Figure 102:**
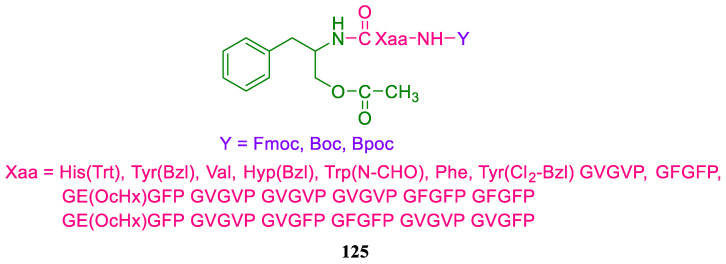
Aurantiamide acetate derivatives with amino acids/peptides.

Suhas R et al. synthesised a novel series of aurantiamide acetate derivatives (**125**) by attaching different amino acids/peptides and varying the N-terminal substituents with different protecting groups in solution-phase method and reported it as a new class of anti-inflammatory and analgesic agents with no ulcerogenic effects (Figure 102). The different N-protecting groups used were Boc, Fmoc and Bpoc (2-(*p*-biphenylyl)-2-propyloxycarbonyl). The anti-inflammatory and analgesic activities of the synthesised conjugates were evaluated by acute carrageenan-induced paw oedema method in rats and tail flick method in mice, respectively. Further, side chain of His, Tyr, Hyp and Trp were protected using Trt (trityl), Bzl/Cl_2_-Bzl, Bzl and CHO, respectively. Pentapeptides and tricosamers were reported to exhibit excellent anti-inflammatory and analgesic activities with the highest potency of 1.47 and 1.24 compared to standard drugs, which exhibited the potency of one for both the activities, viz. phenyl butazone (anti-inflammatory) and ibuprofen (analgesic), with no ulcerogenic liability in Swiss albino mice and Wistar rats. Further, the authors claimed that the presence of bulky groups (such as Fmoc) adjacent to the amino acid side chains is responsible for the enhanced activity [190].

**Figure 103:**
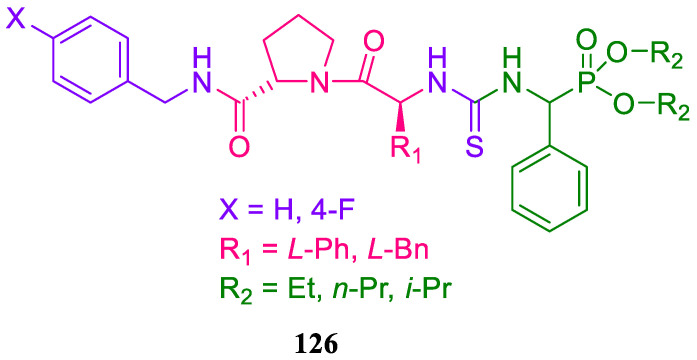
Chiral dipeptide thioureas containing α-amino phosphate analogues.

Liu J et al. synthesised and tested a series of novel chiral dipeptide thioureas containing α-amino phosphate analogues (**126**) as anticancer agents. Substituted benzylamine was coupled with N-Boc-protected Gly and Pro in the presence of HBTU, followed by deprotection using TFA (Figure 103). The dipeptides were formed by coupling with amino acids and converted to thioureas by the nucleophilic addition of α-phosphonate isothiocyanate to *O*,*O*′-dialkylisothiocyanato (phenyl)methylphosphonate. All the conjugates were evaluated for antitumor activity against tumour cell lines (BGC-823 and A-549) using MTT assay. The fluorine-containing compounds exhibited comparable inhibition (20.9 ± 2.8 and 19.2 ± 2.3 µmol/L) with that of *cis*-platin (15.1 ± 2.3 and 17.6 ± 3.1 µmol/L) against BGC-823 and A-549 cell lines. Finally, authors concluded on the subject of structure–activity relationship, reporting: incorporating the rigid amino acid *L*-Pro was more advantageous for anticancer activity. *L*-Phe containing thioureas displayed greater anticancer activity than *L*-phenylglycine containing thioureas. It was found that inserting EWGs in the fourth position of the terminal phenyl group attached to dipeptide thioureas would boost the anticancer efficacy [191].

**Figure 104:**
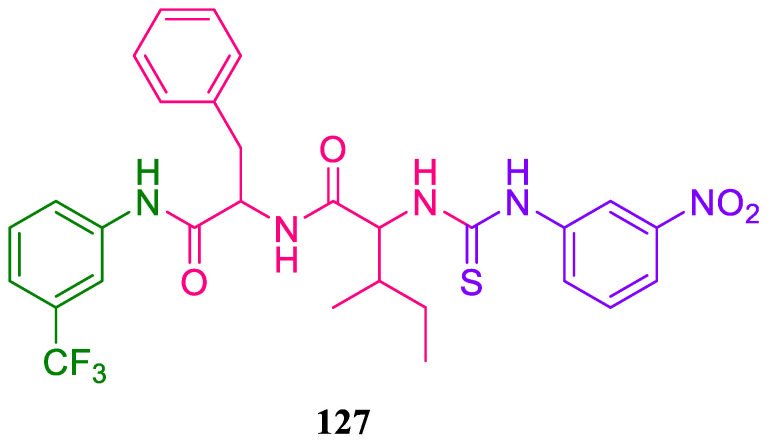
Terminal functionalised dipeptide derivatives.

Qin J M et al. have synthesised terminal functionalised dipeptide derivatives (**127**) having thiourea group as 20S proteasome inhibitors. The reaction goes by the acylation and deprotection of Boc-amide with aniline derivatives, which led to the coupling of amino acids from C- to N-terminus into a peptide chain followed by the deprotection and condensation of intermediate with phenylisothiocyanate in DCM at rt. Finally, the target molecules were achieved by alkylation with methyliodide using K_2_CO_3_ (Figure 104). The lead molecules were further biologically evaluated for the inhibition of 20S proteasome on ChT-L, PGPH and T-LA. The results revealed that the dipeptide derivatives having trifluoromethyl group/fluorine at 3-position in their phenyl ring exhibited a more enhanced ChT-L inhibition nearly four folds than those having methoxy groups. From the structure–activity relationship, it was concluded that the increased activity was observed in the following trend, i.e., bromine < trifluoromethyl < nitro, whereas the introduction of two methoxy groups hindered the activity. The compounds with the best ChT-L inhibitory activity were further evaluated on β_1_ and β_2_ subunits. Furthermore, the synthesised molecules also inhibited PGPH activity but at a lesser extent, whereas there was no inhibition in the case of T-L activity. The cytotoxic activity was performed against NCI-H460 and the results revealed that the molecule having nitro-substituted phenyl ring exhibited enhanced antitumor activity with promising IC_50_ values, i.e., 1.70 ± µM than the standard (doxorubicin). From the molecular docking studies, it was noticed that the compound having nitro-substituted phenyl ring reported the highest docking score of 12.63 when performed on the active site of 20S proteasome. The docking studies exhibited the hydrogen bonding interactions with the Gly92, Gly94 and HOH of the active sites and also showed π-cation/π-π interactions [192].

**Figure 105:**
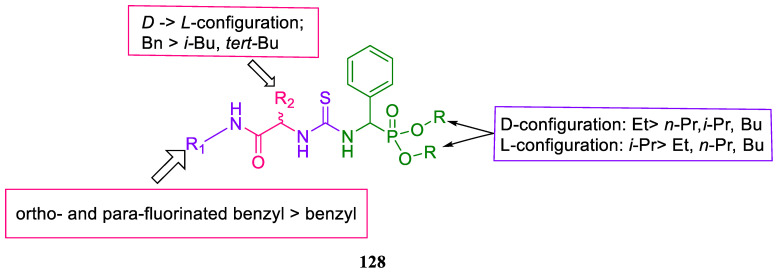
Phosphonate thioureas containing amino acids.

Liao P et al. synthesised a series of novel phosphonate thioureas (**128**) and evaluated their antitumor activity against three human cancer cell lines (Bcap-37, BGC-823 and PC-3) by using MTT colorimetric method with adriamycin (ADM) as reference (Figure 105). Most of the *D*-configured conjugates exhibited better inhibition (greater than three folds) than their *L*-enantiomers. The title compounds containing *D*- or *L*-Phe analogues showed good cellular inhibition as compared with those of *L*-leu or 4-methyl *L*-Leu. The compound (R_1_ = *p*-FBn) showed the best anti-BGC-823 cells activity (89.1% inhibition at 10 μM), whereas the compound (R_1_ = *o*-FBn) exhibited the best inhibitory activity on PC-3 (73.4% inhibition at 10 μM) and Bcap-37 cells (77.2% inhibition at 10 μM). The *D*-configured compounds containing ethyl group of phosphate ester and R_1_ = Bn or *o*-FBn exhibited better activity than the ones consisting bulky moieties, such as *iso*-propyl, propyl and butyl. However, the *L*-configured compounds having *iso*-propyl, R_1_ = *p*-FBn or *o*-FBn presented greater anti-proliferation activity than other conjugates containing propyl, ethyl and butyl groups. The types of amino acids, their configuration and the alkyl structure of phosphonates in the title conjugates appeared to be the key factors in controlling the anti-proliferative activity of cancer cell lines. The compounds containing fluorine groups in the amino acid amide structure showed significant anticancer activity. Authors concluded that the variation of alkyl groups in the novel phosphonate thioureas contribute to their anticancer activity [193].

## 3. Conclusions and Perspective

Nature is believed to offer about 40% of medications in which peptide drugs hold a well-defined space in the therapeutic landscape, among which they can exceed larger biologics and small molecule entities. Peptide-based drug discovery has witnessed a resurgence in interest and scientific momentum in the past two decades, as the healthcare industries have come to recognise the importance of peptide drugs in resolving unmet medical needs, and how this kind of molecules could be a great complement or even a recommended alternative to biologics and small molecule therapeutics. In addition, heterocyclic compounds, too, possess a wide range of applications, primarily in pharmaceuticals. The chief drawbacks of peptides (amino acids) and heterocyclic (small molecule entities) drugs for the clinical usage is attributed to their low bioavailability, poor membrane permeability, minimal metabolic stability in plasma, superior toxicity and dearth specificity. There is a firm intellectual relation between chemical biology and conjugation chemistry. Both research domains integrate the tools and techniques of chemistry to investigate biological frontiers and also to harness biological mechanisms for desired outcomes (e.g., drug delivery). The alliance among heterocycles and amino acids/peptides is best illustrated by the usage of conjugates and conjugation methodologies to address the basic issues regarding biological systems. Yet, perhaps the powerful ‘bond’ among the areas is their dependence upon the atom-by-atom understanding offered by chemistry. Together, these two domains enable unprecedented levels of control to resolve a variety of challenging problems associated with diversified human diseases, especially infections. Encouraging findings from the literature based on ‘conjugation’ have been included in this review, which manifests the progressive growth to tackle deadly diseases. Information provided on amino acids/peptides-small molecule conjugates in this review will definitely open new avenues in drug development to handle the global health crises of infectious diseases.

## Data Availability

Not applicable.

## Abbreviations

Unless otherwise mentioned, amino acids are represented as *L*-isomers and standard three letter code or single letter code has been used; BHA: butylated hydroxyanisole, Boc: *tert*-butyloxycarbonyl, Cbz/Z: benzyloxycarbonyl, CDI: carbonyl diimidazole, DCC: dicyclohexyl carbodiimide, DCM: dichloromethane, DIC: diisopropylcarbodiimide, DIEA/DIPEA: diisopropylethyl amine, DMAP: 4-dimethylaminopyridine, DPPH: 2,2-diphenyl-1-picrylhydrazyl, EDCI: 1-ethyl-3-(3-dimethylaminopropyl)carbodiimide, EDG: electron donating group, EWG: electron withdrawing group, Fmoc: fluorenylmethyloxycarbonyl, GABA: gamma-aminobutyric acid, HOAt: 1-hydroxy-7-azabenzotriazole, HOBt: 1-hydroxybenzotriazole, MTT: 2,5-diphenyl-2H-tetrazolium bromide, NMM: N-methyl morpholine, SPPS: solid-phase peptide synthesis, TBTU: 2-(1H-benzotriazole-1-yl)-1,1,3,3-tetramethyluronium tetrafluoroborate, TEA: triethylamine, TFA: trifluoroacetic acid, THF: tetrahydrofuran.
